# Diazo-Preserving Radical Cascades for the Modular Photoinduced Synthesis of Oxindoles and Pyrazolones

**DOI:** 10.1021/acs.orglett.6c01312

**Published:** 2026-04-27

**Authors:** Pau Sarró, Roser Pleixats, Adelina Vallribera, Carolina Gimbert-Suriñach, Albert Granados

**Affiliations:** Department of Chemistry and Centro de Innovación en Química Avanzada (ORFEO−CINQA), 16719 Universitat Autònoma de Barcelona, Cerdanyola del Vallès, 08193 Barcelona, Spain

## Abstract

We report a metal-free photocatalytic strategy for the modular synthesis of diazo-containing oxindoles and pyrazolones from readily available precursors. Diazomethyl radicals are generated either via a three-component electron donor–acceptor (EDA) complex involving acrylamides or through a photoredox cycle for the activation of hydrazides, enabling C­(sp^2^)–C­(sp^3^) bond formation while preserving the diazo functionality. The method tolerates a broad range of functional groups, substitution patterns, and pharmaceutically relevant motifs. Mechanistic studies, including radical trapping, UV–vis spectroscopy, light on/off experiments, and Stern–Volmer analysis, support a radical chain propagation and distinguish the EDA-driven versus photoredox pathways. The resulting diazo heterocycles undergo downstream transformations to access new chiral centers and functional motifs.

## Graphical abstract

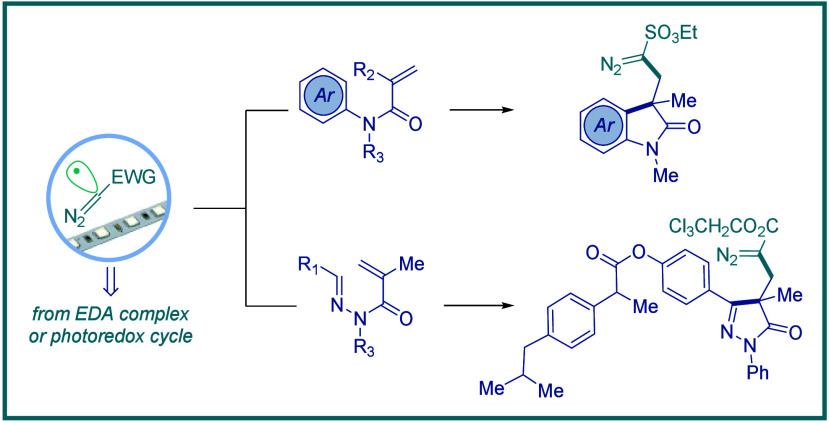

Diazo compounds are popular and versatile intermediates employed in organic chemistry as functionalization sites. Their high intrinsic reactivity and multiple activation modes enable a wide range of transformations, making them powerful platforms for molecular functionalization.^1^ Among these, the generation and transfer of diazomethyl radicals have emerged as an attractive strategy for the installation of diazo groups under catalytic conditions. In 2018, Suero and co-workers^2^ introduced I­(III)-substituted diazo compounds^3^ as efficient precursors for diazomethyl radicals. Under Ru-photoredox catalysis, these reagents generate carbon-centered radicals that enable the direct diazomethylation of electron-rich arenes and heteroarenes. Subsequently, Alcarazo reported a complementary class of precursors based on sulfonium salts in which the sulfonium unit serves as an effective leaving group to facilitate radical formation.^4^ Despite these advances, most reported methods do not preserve the diazo functionality in the final product, and examples of C­(sp^2^)–C­(sp^3^) bond formation remain scarce.^5,6^

An appealing alternative strategy for radical generation involves the formation of photoactive electron donor–acceptor (EDA) complexes. In this activation mode, the association between an electron donor and an electron acceptor forms a ground-state molecular aggregate that can be excited by visible light to produce a radical ion pair. Subsequent irreversible fragmentation generates highly reactive radical species capable of engaging in diverse transformations (Figure 1A).^7^ In this context, sulfonium salts and hypervalent iodine reagents have proven to be particularly effective electron acceptors for the formation of robust EDA complexes.^8^ Of note, the construction of diazoalkylated units via C­(sp^2^)–C­(sp^3^) are essentially unknown through photoinduced EDA-complexes.^9^

**Figure 1:**
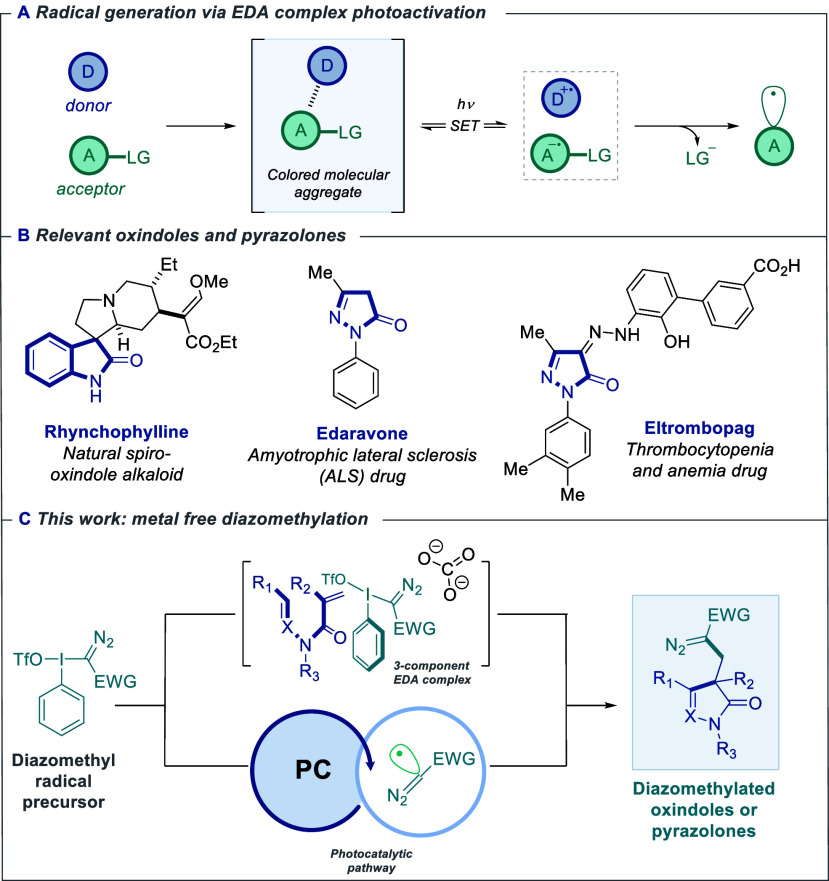
(A) Photoactivation of Electron Donor-Acceptor (EDA) complexes. LG = leaving group. (B) Relevant examples of oxindoles and pyrazolones. (C) This approach.

Oxindoles and pyrazolones represent privileged heterocyclic frameworks that are frequently encountered in medicinal chemistry and play an important role in the discovery of bioactive molecules (Figure 1B).^10^ Diazo-containing derivatives of these scaffolds are particularly attractive because the diazo group provides a versatile handle for further derivatization. However, this functionality is typically confined to the C3 position of oxindoles and remains rarely explored in pyrazolone derivatives.^11^ Consequently, the direct construction of C­(sp^2^)–C­(sp^3^) bonds that simultaneously install a diazo functionality outside the core of oxindole and pyrazolone frameworks remains an unmet synthetic challenge.

Herein, we report mild and operationally simple strategies for the synthesis of structurally complex diazo-functionalized oxindole and pyrazolone derivatives. Mechanistically, the transformation proceeds through two distinct pathways depending on the substrate. Acrylamides form a three-component EDA complex that enables the visible-light-driven generation of diazomethyl radicals from I­(III) precursors, whereas hydrazide substrates react via a photoredox pathway (Figure 1C). These approaches enable the formation of C­(sp^2^)–C­(sp^3^) bonds while installing diazo groups outside the heterocyclic core, providing previously inaccessible entry to diazo-functionalized oxindole and pyrazolone scaffolds.

Optimization of reaction conditions and radical precursor identified linear hypervalent iodine reagents and Na_2_CO_3_ under white LEDs as the optimal conditions for this oxindole synthesis (see Table S1 in the ESI). With suitable conditions established, the substrate scope was evaluated by using various *N*-arylacrylamides (Table 1). Both electron-donating and electron-withdrawing substituents in the aromatic ring were well tolerated, affording the desired oxindoles in generally good yields. Representative examples include alkyl- and methoxy-substituted derivatives (**3c**–**d**) as well as *para*-halogenated substrates (**3e**–**g**). A nitrile-substituted substrate also proved compatible (**3h**), albeit with a slightly diminished efficiency. Notably, substrates bearing multiple halogens or *ortho*-substitution led to reduced yields (**3i**–**k**), highlighting the sensitivity of the reaction to electronic and steric effects. In the case of **3i**, improved outcomes were achieved using 4DPAIPN as a photocatalyst. Variation at the α-carbonyl position and alternative *N*-functionalized acrylamides were also compatible, delivering the corresponding products in good yields (**3l**–**n**). Additionally, a substrate lacking α-alkyl substitution furnished cyclopropane derivative **3o**, demonstrating the versatility of the system. Furthermore, a range of electron-withdrawing groups on the diazo reagent were compatible, providing access to structurally diverse products (**3a**–**b**, **3p**–**t**). Extension to *N*-methacrylohydrazides enabled access to diazo-containing pyrazolones under photocatalytic conditions (see Table S2). Both *N*-aryl and *N*-alkyl substrates were well tolerated, including those bearing electron-donating and electron-withdrawing groups (**3w**–**x**) as well as derivatives of pharmaceutically relevant compounds such as ibuprofen and gemfibrozil (**3y**–**z**). IR bands (ca. 2100 cm^–1^) confirm that the diazo moiety is preserved in all cases (see the ESI).

**Table 1:**
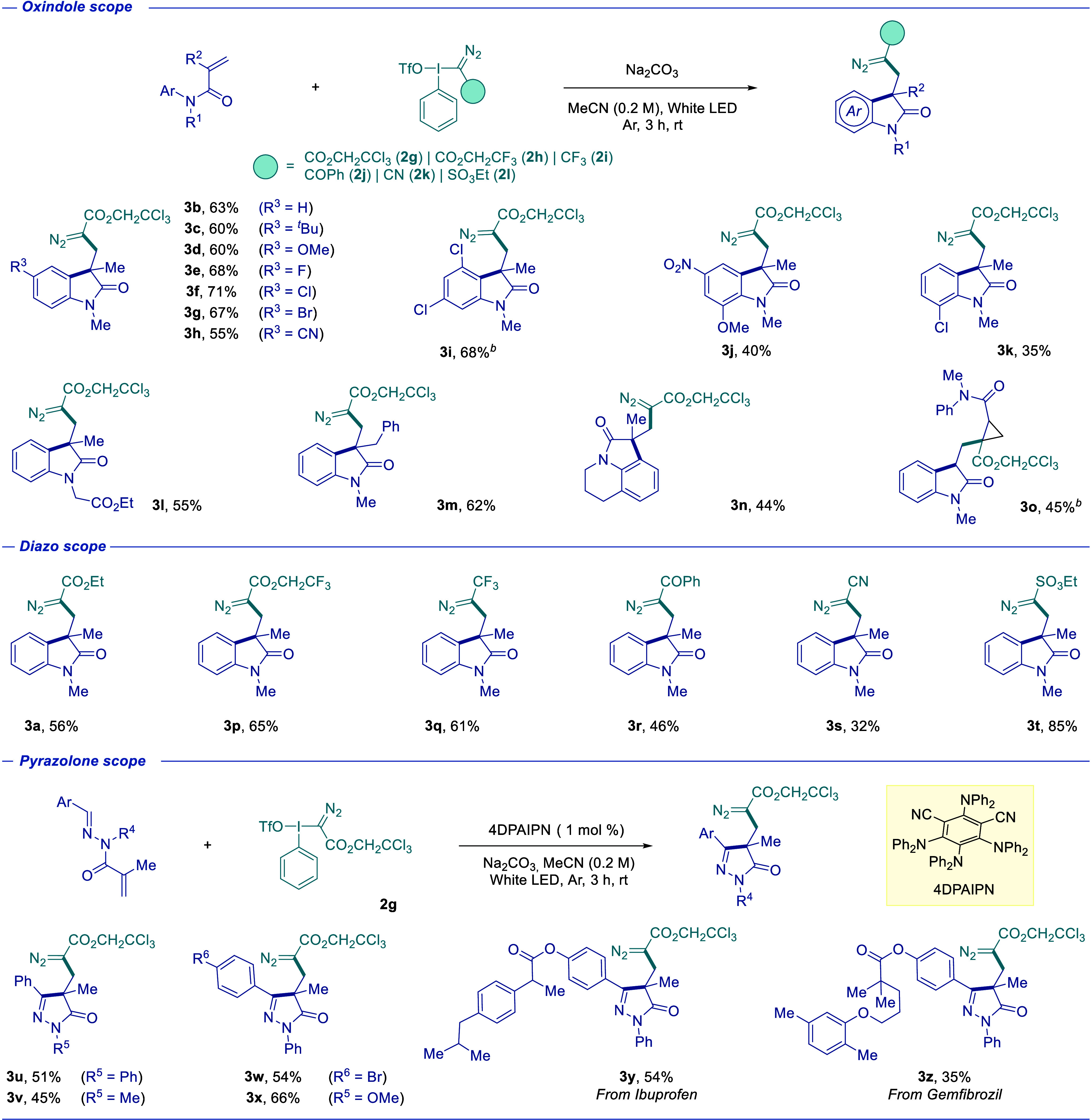
Substrate Scope Study^a^

a Reaction conditions: Radical acceptor (0.6 mmol, 3 equiv), hypervalent iodine reagent (0.2 mmol, 1 equiv), Na_2_CO_3_ (0.2 mmol, 1 equiv) in MeCN (1 mL, *c* = 0.2 M) under white LED irradiation for 3 h at room temperature.

b Using 4DPAIPN (1 mol %) as photocatalyst. Isolated yields.

Mechanistic experiments were conducted to probe the reaction pathway (Figure 2). Under the standard oxindole-forming conditions, the addition of 2,6-di-*tert*-butyl-4-methylphenol (BHT) completely suppressed product formation, consistent with the involvement of radical intermediates. To probe the involvement of an EDA complex, UV/vis absorption studies were performed. Spectroscopic analysis of the individual components and their mixtures in MeCN suggested the formation of a charge-transfer complex among **1a**, hypervalent iodine diazo compound **2g**, and Na_2_CO_3_ (Figure 2A and S9). Whereas acrylamide **1a** showed absorption in the near-UV region and **2g** absorbed at λ < 500 nm, the mixture containing all reaction components exhibited a marked bathochromic shift, consistent with EDA complex formation and its activation under white LED irradiation (orange trace, Figure 2A). Light on/off experiments provided further mechanistic insight. After 30 min of irradiation, the product yield reached 30% and increased to 46% after a subsequent 30 min period in the dark. This result is consistent with photoinitiation followed by an efficient radical chain process (Figure 2B).

**Figure 2:**
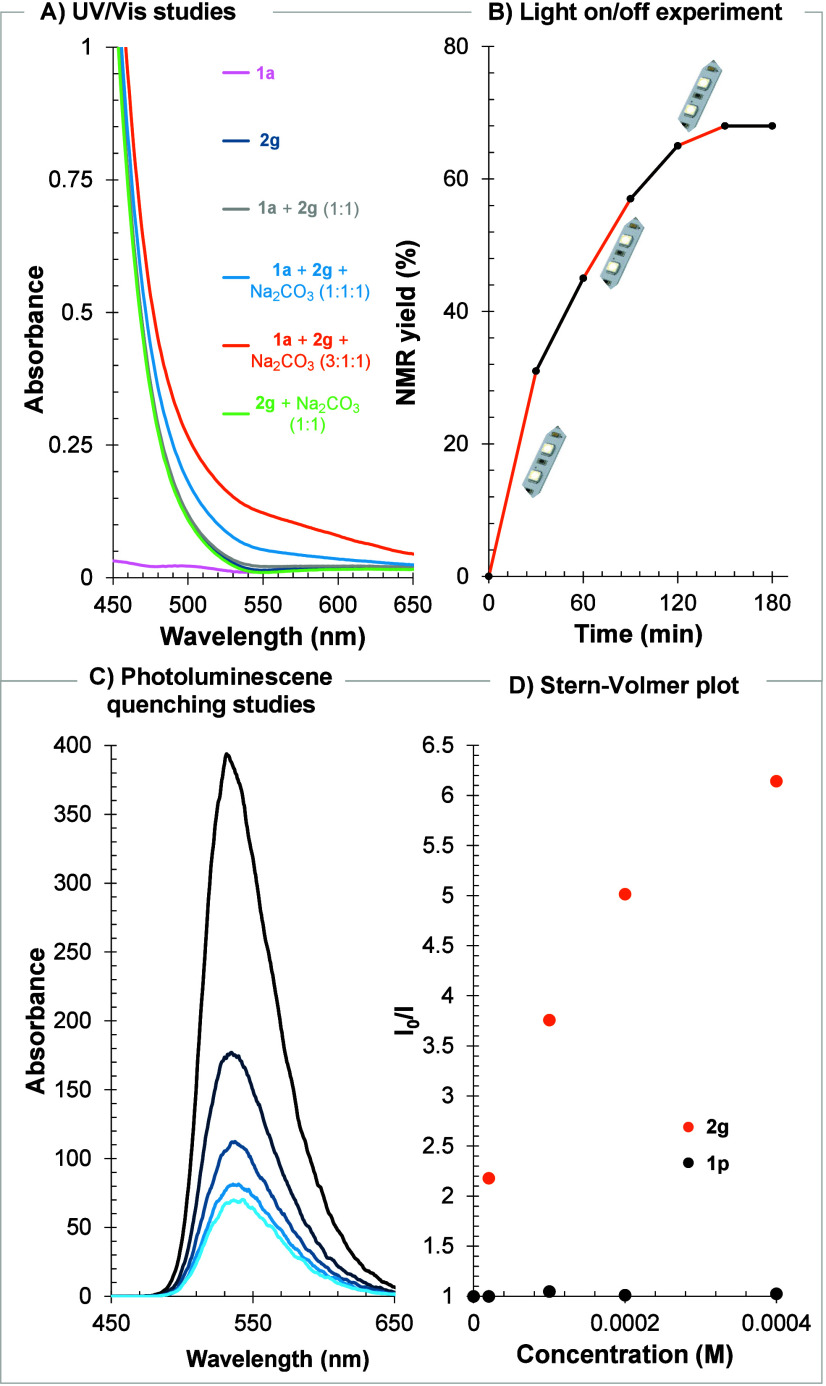
(A) UV/vis studies of **1a** (0.05 M), **2a** (0.05 M) and their mixtures. (B) Light on/off experiment. (C) PL quenching studies. (D) Stern–Volmer plot (see ESI for details).

To probe the photocatalytic pathway using 4DPAIPN, Stern–Volmer luminescence quenching studies were performed. The results (Figure 2C,D and Figures S6–S8 in the ESI) showed that diazo compound **2g** efficiently quenched the excited state of 4DPAIPN (*E*_red_* = −1.66 V vs SCE),^12^ whereas *N*-aryl-*N*-methacrylohydrazide **1p** displayed negligible quenching. These findings identify **2g** as the primary quencher of the photocatalyst and are consistent with an oxidative quenching pathway.

On the basis of these mechanistic experiments, a plausible reaction pathway is proposed for the formation of oxindoles and pyrazolones (Figure 3). For oxindole synthesis, a ground-state EDA complex is proposed to be formed by *N*-arylmethylacrylamide and a hypervalent iodine reagent via π interactions with carbonate acting as an additional electron donor.^8f^ Upon visible-light irradiation, SET within this complex generates diazomethyl radical **C**, which adds to the alkene to give intermediate **D**. Subsequent intramolecular cyclization affords radical intermediate **E**, which is proposed to react with the hypervalent iodine reagent **2** to regenerate radical **C** and form cationic intermediate **F**. Deprotonation of **F** then delivers the oxindole product. These results are consistent with photoinitiation followed by radical chain propagation. In contrast, for pyrazolone synthesis under 4DPAIPN catalysis, excitation of the photocatalyst is followed by SET reduction of diazo precursor **2**, generating diazomethyl radical **C**. Addition of **C** to the electron-deficient alkene gives radical intermediate **G**, which undergoes 5-endo-trig cyclization to form *N*-centered radical **H**. Oxidation of **H**, either by the oxidized photocatalyst (*path A*) or by reagent **2** (*path B*), followed by deprotonation, furnishes the pyrazolone product. In line with the results obtained for the EDA pathway, a radical chain process may also operate under these conditions.

**Figure 3:**
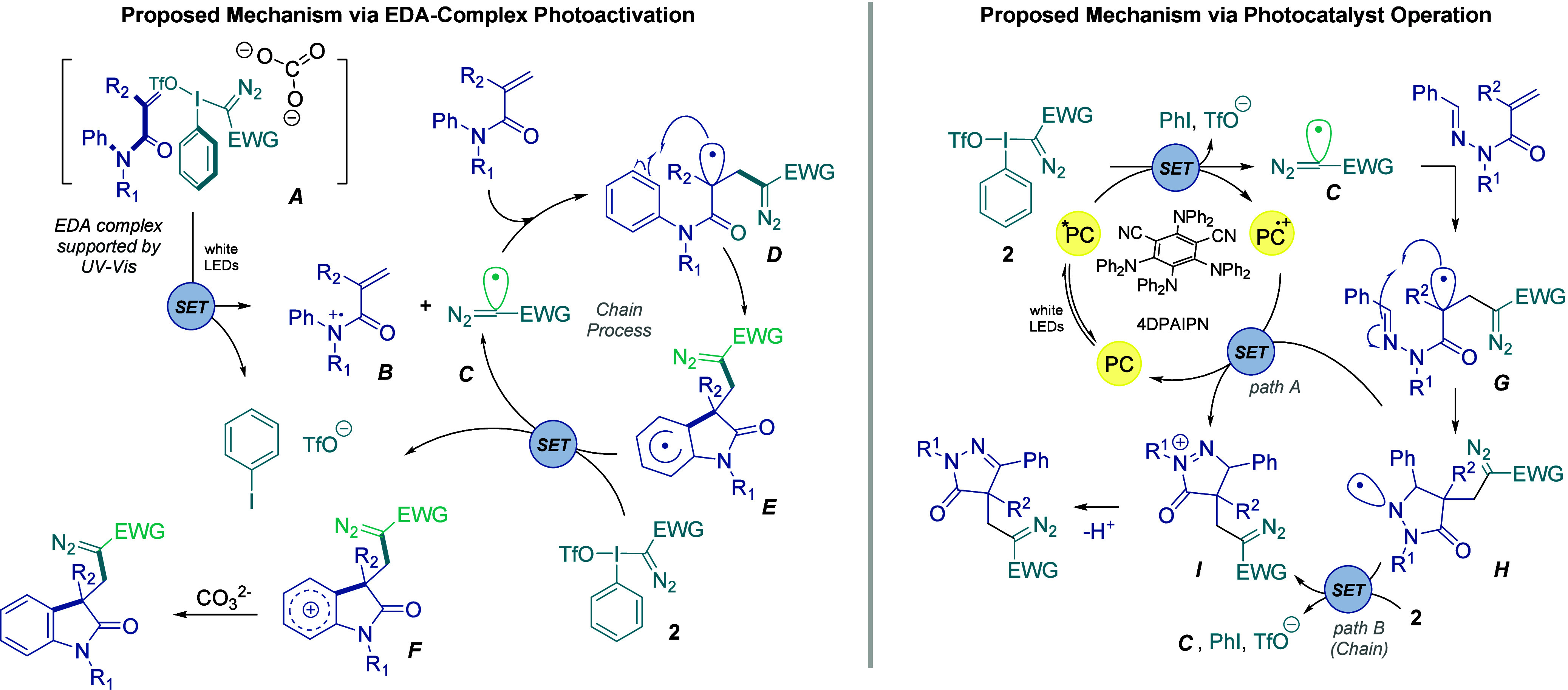
Proposed mechanistic scenarios for the synthesis of diazomethyl-containing oxindoles and pyrazolones.

The synthetic potential of novel diazo-oxindole compound **3f** was explored to access new chiral centers bearing synthetically relevant motifs (Figure 4). First, we accessed **3f** from a 1.0 mmol scale in 64% yield (see ESI for details). Then, aniline **4** and trifluoromethylated derivative **5** were obtained via ruthenium- and copper-catalyzed transformations in 72% and 65% yield, respectively, forming new C–N and C–C bonds. Subsequently, alkene **6** was synthesized by using Cs_2_CO_3_ in acetone under 456 nm irradiation, and a C–O bond was formed (**7**) via copper catalysis in 42% yield.

**Figure 4:**
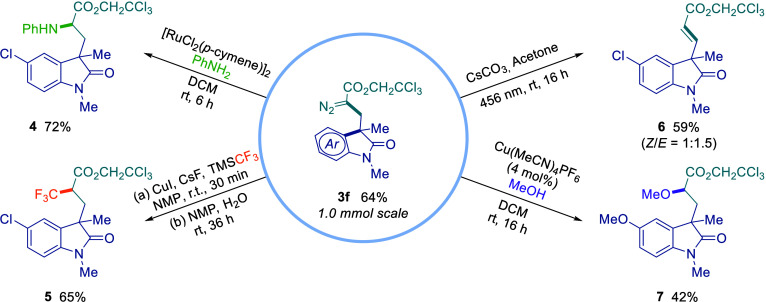
Synthetic transformations of diazo-oxindole **3f** provide access to valuable derivatives (**4**–**7**). Isolated yields.

In summary, we developed a metal-free photocatalytic strategy for the diazomethylation of acrylamide and hydrazide derivatives, providing direct access to diazo-containing oxindoles and pyrazolones under mild conditions. Notably, the diazo group is preserved throughout the cascade, enabling further derivatization. Mechanistic studies support two distinct activation modes: EDA complex-driven radical generation in the absence of a photocatalyst and a 4DPAIPN-mediated photoredox pathway in which the diazo precursor acts as the primary quencher of the excited state. Overall, the results are consistent with radical chain propagation. This work expands the scope of diazomethyl radical chemistry, enabling the formation of a C­(sp^2^)–C­(sp^3^) bond and provides a modular and operationally simple route to diazo-functionalized heterocycles.

## Supplementary Material



## Data Availability

The data underlying this study are available in the published article and its Supporting Information.

## Notes

Experimental details including general experimental information and experimental procedures, characterization data, and NMR data (PDF)

## References

[1] Ford A., Miel H., Ring A., Slattery C. N., Maguire A. N., McKervey M. A. (2015). Modern Organic Synthesis with α-Diazocarbonyl
Compounds. Chem. Rev..

[2] Wang Z., Herraiz A. G., del Hoyo A. M., Suero M. G. (2018). Generating carbyne
equivalents with photoredox catalysis. Nature.

[3] Weiss R., Seubert J., Hampel F. (1994). α-Aryliodonio
Diazo Compounds: SN Reactions at the α-C Atom as a Novel Reaction
Type for Diazo Compounds. Angew. Chem., Int.
Ed. Engl..

[4] Li X., Golz C., Alcarazo M. (2021). α-Diazo Sulfonium Triflates:
Synthesis, Structure, and Application to the Synthesis of 1-(Dialkylamino)-1,2,3-triazoles. Angew. Chem., Int. Ed..

[5] Xu X.-C., Wu D.-N., Liang Y.-X., Yang M., Yuan H.-Y., Zhao Y.-L. (2022). Visible Light-Induced Coupling Cyclization
Reaction of α-Diazosulfonium Triflates with α-Oxocarboxylic
Acids or Alkynes. J. Org. Chem..

[6] He Q., Zhang Q., Rolka A. B., Suero M. G. (2024). Alkoxy Diazomethylation
of Alkenes by Photoredox-Catalyzed Oxidative Radical-Polar Crossover. J. Am. Chem. Soc..

[7] Crisenza G. E. M., Mazzarella D., Melchiorre P. (2020). Synthetic Methods Driven by the Photoactivity of Electron
Donor–Acceptor Complexes. J. Am. Chem.
Soc..

[8] van Dalsen L., Brown R. E., Rossi-Ashton J. A. (2023). Sulfonium Salts as Acceptors in Electron
Donor-Acceptor Complexes. Angew. Chem., Int.
Ed..

[9] Zhang Z.-Q., Li X., Gao S.-F., Cai B.-G., Li Q.-Q., Xuan J. (2025). Annulation of α-Diazo Sulfonium
Salts with α-Vinylanilines via a Dicationic Intermediate toward
Quinolines. Org. Lett..

[10] Lei T., Wang J.-Y., Pei J. (2014). Design, Synthesis,
and Structure–Property Relationships of Isoindigo-Based Conjugated
Polymers. Acc. Chem. Res..

[11] Yu B., Yu D.-Q., Liu H.-M. (2015). Spirooxindoles:
Promising scaffolds for anticancer agents. Eur.
J. Med. Chem..

[12] Gil-Ordóñez M., Gallego-Gamo A., Sarró P., Pleixats R., Gimbert-Suriñach C., Vallribera A., Granados A. (2025). Organophotoredox-Driven Three-Component
Synthesis of β-Trifluoromethyl β-Amino Ketones. J. Org. Chem..

