# Supplementary material for: Diazo-Preserving Radical Cascades for the Modular Photoinduced Synthesis of Oxindoles and Pyrazolones
Source: Org Lett. 2026 Apr 27;28(18):5816–21. doi: 10.1021/acs.orglett.6c01312 (PMC13162318; doi:10.1021/acs.orglett.6c01312)
Supplement: Supplementary file 1 [file ol6c01312_si_001.pdf]

Supporting Information

Diazo-Preserving Radical Cascades for the Modular Photoinduced Synthesis of Oxindoles and Pyrazolones

Pau Sarró, Roser Pleixats, Adelina Vallribera,* Carolina Gimbert-Suriñach*and Albert Granados*

Departament de Química and Centro de Innovación en Química Avanzada (ORFEO-CINQA), Universitat Autònoma de Barcelona, Cerdanyola del Vallès, 08193 Barcelona, Spain

*To whom correspondence should be addressed.

adelina.vallribera@uab.es carolina.gimbert@uab.es albert.granados@uab.es

TABLE OF CONTENT

1. General Considerations S2

2. List of Substrates 1 and Diazo Compounds **2** S3

3. Diazomethylation of Acrylamides and Hydrazides: Reaction Workflow, General Procedures and Compound Characterization S4

4. Large scale synthesis of **3f** S27

5. Mechanistic Investigation S27

6. NMR Spectra S36

1. General Considerations

1.1 General

All chemical transformations requiring inert atmosphere were done using Schlenk line techniques with a 4- or 5-port dual-bank manifold. For white light irradiation, a Wobane 12 W LED strip light was used. Photoinduced reactions were performed using 4 or 8 mL Chemglass vials (15-425 Green Open Top Cap, TFE Septa). For blue light irradiation, a Kessil PR160-purple LED lamp (40 W High Luminous DEX 2100 LED, λmax = 456 nm) was placed 4 cm away from the reaction vials. Reactions were monitored by TLC or NMR. TLC analysis was performed using hexanes or pentane with EtOAc or Et_2O as the eluents and visualized using UV light and Vanillin or $KMnO_4$ solutions. The UV-Vis spectra were recorded in a UV-Vis spectrophotometer Cary 60 at room temperature, with the appropriate solvent. The NMR experiments (^1H, ^{13}C, ^{11}B {^1H decoupled} and ^{19}F {^1H decoupled}) were performed in the *Servei de Ressonància Magnètica Nuclear*, UAB, using NEO 400, NEO 500 or NEO 600 NMR spectrometers. Chemical shifts are referenced to residual, nondeuterated $CHCl_3$ (δ 7.26 in ^1H NMR and 77.3 in ^{13}C NMR). The cyclic voltammetry (CV) experiments were performed with a BioLogic SP-50 Single Channel Potentiostat in a one-compartment three-electrode setup using a glassy carbon disk as the working electrode (ø = 3 mm), platinum wire as the auxiliary electrode, and SCE or $AgNO_3$/Ag (0.01 M $AgNO_3$, 0.1 M [$^nBu_4N$]PF_6 ($TBAPF_6$), MeCN) as reference electrodes. CV were performed at room temperature using the appropriate solvent, degassing with argon for 60 s and using $TBAPF_6$ as supporting electrolyte (0.1 M). All the experiments were referred to ferrocene as an internal standard. Polishing of the working electrode has been done using an alumina polishing pad with a solution of 0.05 µm alumina in water (purchased from BAS INC.). Fluorescence measurements were obtained using septa-capped UV-Quartz cuvettes (10 mm path length) from Hellma Analytics and were recorded in a PerkinElmer LS 55 Fluorescence Spectrometer attached to a PTP 1 Peltier Temperature Programmer maintaining the temperature at 25 °C. The HRMS (ESI+) and elemental analyses were done by the *Parque Científico Tecnológico*, UBU. HRMS was done using a Bruker microTOF-QII mass spectrometer (fly time analyzer) through positive electrospray ionization. IR spectra were recorded on a Bruker Alpha II ATR-IR using either neat oil or solid products. Melting points (°C) were measured in an MPM-H2 Kleinfeld. The melting point values are uncorrected. **Caution:** Diazo compounds are potentially hazardous and may decompose explosively. Reactions should be conducted on small scale with appropriate safety precautions. Diazo-containing products should be stored at low temperature and protected from light.

1.2 Chemicals

Deuterated NMR solvents were purchased from Euroisotop. Dry solvents were obtained from Aldrich and used as received. Bulk DCM, EtOAc, pentane and hexane were purchased from VWR. Chemicals were purchased from Chemosapiens and used as received unless specified.

2. List of Substrates 1 and Diazo Compounds 2

Acrylamides **1a, 1c-g, 1j, 1l-n** were synthesized following the reported protocol from Jiao,[1] **1b, 1h**, **1k** from Zhu,[2] **1o, 1q-t** from Yu[3] and **1p** from Murarka.[4] **1i** was synthesized following *General Procedure A*.

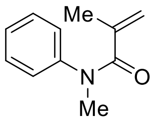

1a

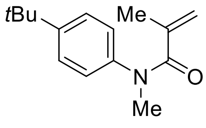

1b

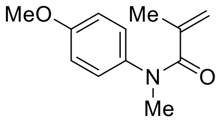

1c

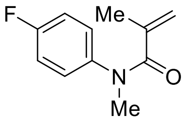

1d

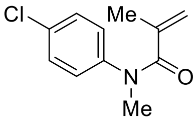

1e

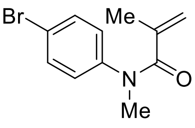

1f

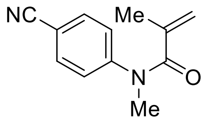

1g

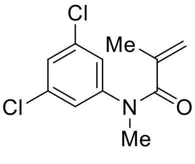

1h

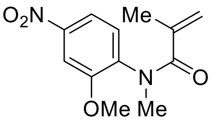

1i

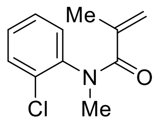

1j

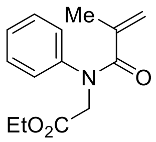

1k

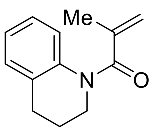

1l

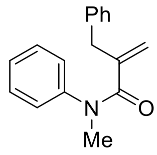

1m

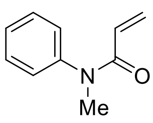

1n

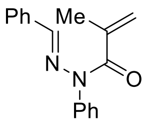

1o

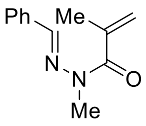

1p

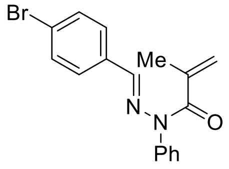

1q

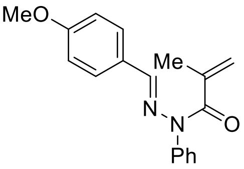

1r

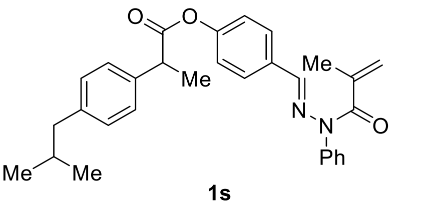

1s

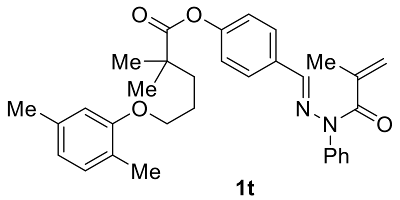

1t

Hypervalent iodine compounds **2a**, **2b**, **2g**, **2i**, **2j** and **2l** were prepared as previously reported by Suero.[5] **2c** was prepared as previously reported by Weiss.[6] Sulfonium salts **2d** – **2f** were prepared as previously reported by Alcarazo.[7] **2h** and **2k** were prepared as previously reported by Glorius.[8] All diazo compounds were kept at -30 ºC.

---

[1] Guo, Y.; Ma, S.; Shi, L.; Liu, L.; Lei, X.; Jiao, P. *Org. Chem. Front.* **2023**, *10*, 2257-2262.
[2] Yuan, W.; Qu, A.; Li, Y.; Li, H.; Chen, K.; Zhu, Y. *Adv. Synth. Catal.* **2022**, *364*, 3932-3940.
[3] Yu, S.; Cheng, Y.; Pan, C.; Yu, J.-T. *Chem. Commun.* **2025**, *61*, 1196-1199.
[4] Panda, S. P.; Dash, R.; Hota, S. K.; Murarka, S. *Org. Lett.* **2024**, *26*, 3667-3672.
[5] (a) Wang, Z., Herraiz, A. G.; del Hoyo, A. M.; Suero, M. G. *Nature* **2018**, *554*, 86-91. (b) Jiang, L.; Wang, Z.; Armstrong, M.; Suero, M. G. *Angew. Chem. Int. Ed.* **2021**, *60*, 6177-6184.
[6] Weiss, R.; Seubert, J.; Hampel, F. *Angew. Chem. Int. Ed.* **1994**, *33*, 1952-1953.
[7] Li, X.; Golz, C.; Alcarazo, M. *Angew. Chem. Int. Ed.* **2021**, *60*, 6943-6948.
[8] Wu, F.-P.; Chintawar, C. C.; Lalisse, R.; Mukherjee, P.; Dutta, S.; Tyler, J.; Daniliuc, C. G.; Gutierrez, O.; Glorius, F. *Nat. Catal.* **2024**, *7*, 242-251.

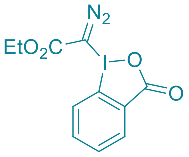

2a

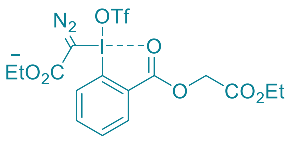

2b

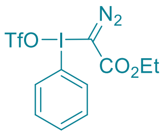

2c

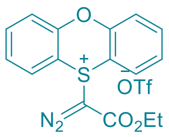

2d

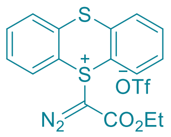

2e

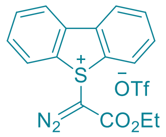

2f

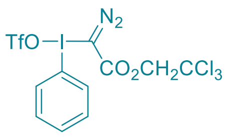

2g

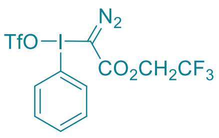

2h

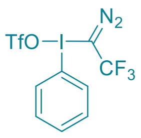

2i

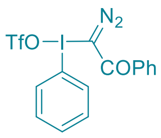

2j

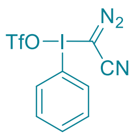

2k

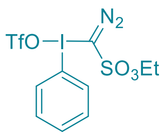

2l

3. Diazomethylation of Acrylamides and Hydrazides: Reaction Workflow, General Procedures and Compound Characterization

3.1. Reaction Workflow

All photoinduced reactions were done using a Wobane 12 W LED strip light. The LED was placed 4 cm away from the reaction vial within a ventilated fume hood and using a fan to maintain the temperature approximately at 25ºC.

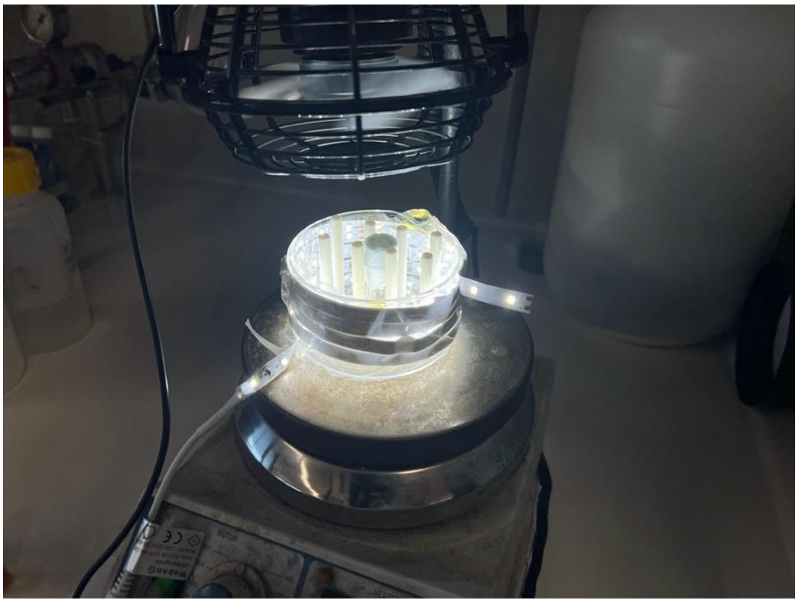

Figure S1. Reaction setup for the photoinduced diazomethylation of alkenes

3.2. General Procedure A: Synthesis of Acrylamides

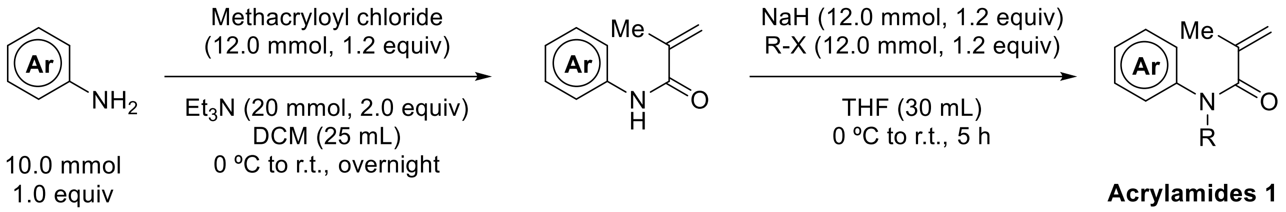

Acrylamides **1** were synthesized following a modified 2-step-protocol from the literature.[9]

Step 1: To a solution of the corresponding aniline (10.0 mmol, 1.0 equiv) in DCM (25 mL) was added Et_3N (2.8 mL, 20.0 mmol, 2.0 equiv) at 0 °C. Next, methacryloyl chloride (1.2 mL, 12.0 mmol, 1.2 equiv) was added dropwise, and the resulting solution was stirred at room temperature overnight. The reaction mixture was poured into H_2O, and the aqueous layer was extracted with DCM (3×10 mL). The combined organic layers were dried over Na_2SO_4, filtered, and concentrated under reduced pressure. The crude product was used in the next step without further purification.

Step 2: To a solution of the corresponding amide (5.0 mmol, 1.0 equiv) in THF (15 mL) was added portionwise NaH (0.24 g, 6.0 mmol, 1.2 equiv, 60 % in mineral oil) at 0 °C. After stirring for 15 min at the same temperature, the corresponding electrophile (MeI or R–X, 6.0 mmol, 1.2 equiv) was added. Then, the mixture was warmed to rt and stirred for an additional 5 h. After reaction completion, the mixture was quenched by H_2O (10 mL), and extracted with EtOAc (3×10 mL). The combined organic layers were dried (Na_2SO_4), filtered, and concentrated under reduced pressure. The crude product was purified by flash column chromatography using hexanes/EtOAc mixtures.

3.3. General Procedure B: Photoinduced Diazomethylation of Alkenes

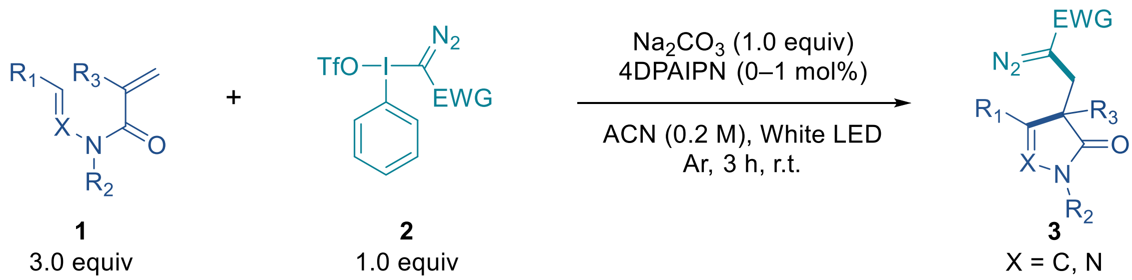

[9] Majhi, J.; Granados, A.; Matsuo, B.; Ciccone, V.; Dhungana, R. K.; Sharique, M.; Molander, G. A. *Chem. Sci.* **2023**, *14*, 897-902.

To a 4 mL Chemglass vial equipped with a magnetic stir bar, radical acceptor **1** (0.60 mmol, 3.0 equiv), diazo reagent **2** (0.20 mmol, 1.0 equiv), Na_2CO_3 (20.0 mg, 0.20 mmol, 1.0 equiv) and 4DPAIPN when necessary (1.6 mg, 1 mol %) were added. Subsequently, the vial was subjected to 3 cycles of vacuum/argon, followed by the addition of dry MeCN (1.0 mL, 0.2 M). The resulting reaction mixture was first degassed with Ar for 30 s and then irradiated for 3 h with a strip of white LED as described in the "*Workflow*" section. The temperature of the reaction was maintained at approximately 25 °C with the use of a fan. Upon completion, the solvent was removed under reduced pressure and the crude mixture was subjected to flash column purification on silica gel using mixtures of hexanes or pentane with EtOAc, Et_2O or DCM to yield the desired product. Some products required the use of a preparative TLC to purify them completely due to their similar polarity to the radical acceptors that are in excess.

3.4. Oxindole Synthesis – Optimization

Table S1 *Optimization of the reaction for the synthesis of oxindoles*

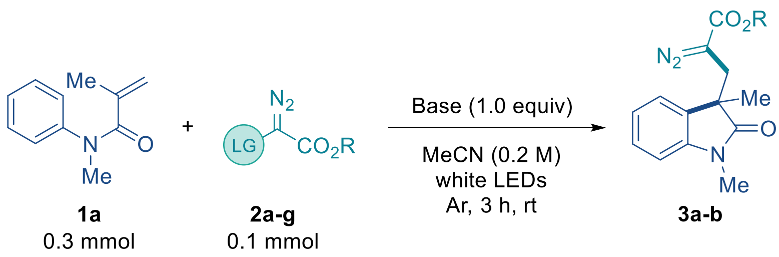

Entry	Diazo source	Base	Yield (%)[a]
1	**2a**	Na_2CO_3	0
2	**2b**	Na_2CO_3	37
3	**2c**	Na_2CO_3	60 (56)[c]
4	**2g**	Na_2CO_3	63 (58)[c]
5	**2d**	Na_2CO_3	4
6	**2e**	Na_2CO_3	3
7	**2f**	Na_2CO_3	0
8	**2c**	DIPEA	0
9	**2c**	DABCO	0
10	**2c**	$NaHCO_3$	0
11	**2c**	K_3PO_4	43
12	**2c**	none	0
13[c]	**2c**	Na_2CO_3	0

[a]NMR yield determined by using 1,3,5-trimethoxybenzene as internal standard. [b]Isolated yield in parenthesis. [c]No light.

To a 4 mL vial equipped with a magnetic stir bar, radical acceptor **1a** (52.6 mg, 0.30 mmol, 3.0 equiv), diazo reagent **2a-g** (0.10 mmol, 1.0 equiv) and the chosen base (0.10 mmol, 1.0 equiv) were added. Subsequently, the vial was subjected to 3 cycles of vacuum/argon, followed by the addition of dry ACN (0.5 mL, 0.2 M). The resulting reaction mixture was first degassed with Ar for 30 s and then irradiated for 3 h with a strip of white LED as described in the "*Workflow*" section. The temperature of the reaction was maintained at

approximately 25 °C with the use of a fan. Upon completion, the solvent was removed under reduced pressure and the crude mixture was analysed by ^{1}H-NMR, obtaining the NMR yield by using 1,3,5-trimethoxybenzene as internal standard. If necessary, the crude mixture was subjected to flash column purification on silica gel using mixtures of hexanes or pentane with EtOAc, Et_2O or DCM to yield the desired product. Some products required the use of a preparative TLC to purify them completely due to their similar polarity to the radical acceptors that are in excess.

3.5. Pyrazolone Synthesis – Optimization

***Table S2** Photocatalysts screening for the synthesis of pyrazolones*

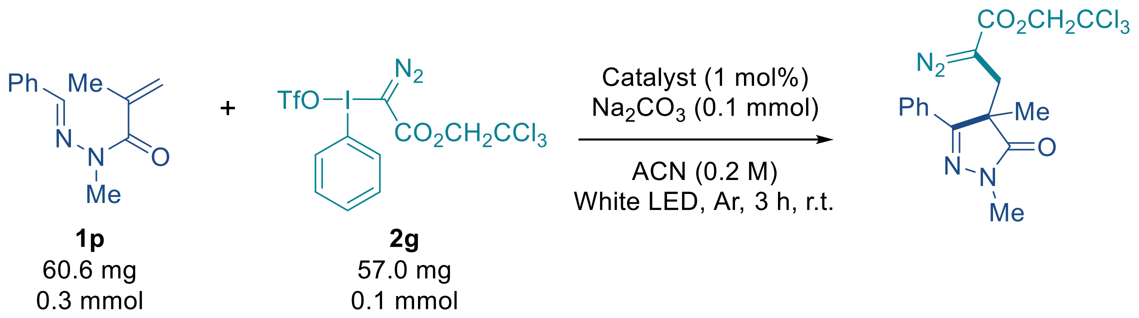

Entry	Light	Catalyst	Diazo source	Yield (%)^{a}
1	White LED	-	**2g**	28
2	White LED	$Ru(bpy)_3PF_6$	**2g**	44
3	White LED	$Ir(ppy)_3$	**2g**	34
4	White LED	4CzIPN	**2g**	29
5	White LED	4DPAIPN	**2g**	47 (42)

^{a}NMR yield; isolated yield in parenthesis.

To a 4 mL vial equipped with a magnetic stir bar, radical acceptor **1p** (60.6 mg, 0.30 mmol, 3.0 equiv), diazo reagent **2g** (57.0 mg, 0.10 mmol, 1.0 equiv), Na_2CO_3 (10.0 mg, 0.10 mmol, 1.0 equiv) and the chosen photocatalyst (1 mol %) were added. Subsequently, the vial was subjected to 3 cycles of vacuum/argon, followed by the addition of dry ACN (0.5 mL, 0.2 M). The resulting reaction mixture was first degassed with Ar for 30 s and then irradiated for 3 h with a strip of white LED as described in the "*Workflow*" section. The temperature of the reaction was maintained at approximately 25 °C with the use of a fan. Upon completion, the solvent was removed under reduced pressure and the crude mixture was analysed by ^{1}H-NMR, obtaining the NMR yield by using 1,3,5-trimethoxybenzene as internal standard. If necessary, the crude mixture was

subjected to flash column purification on silica gel using mixtures of hexanes or pentane with EtOAc, Et_2O or DCM to yield the desired product. Some products required the use of a preparative TLC to purify them completely due to their similar polarity to the radical acceptors that are in excess.

3.6. Compound Characterization Data

***N-(2-methoxy-4-nitrophenyl)-N-methylmethacrylamide* (1i)**

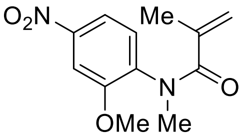

Prepared according to the *General Procedure A* from the corresponding 2-methoxy-4-nitroaniline (1.68 g, 10.0 mmol, 1.0 equiv). After purification by flash column chromatography (silica gel, hexane/AcOEt 4:1 – 3:1), the title compound **1i** was obtained as a yellow solid (2.05 g, 8.2 mmol, 82 %). **R_f** = 0.12 (silica gel, hexane/AcOEt, 4:1 (v/v)). **^{1}H NMR** (500 MHz, $CDCl_3$), δ (ppm) = 7.85 (d, *J* = 7.2 Hz, 1H), 7.77 (s, 1H), 7.27 (d, *J* = 7.2 Hz, 1H), 4.99 (s, 1H), 4.88 (s, 1H), 3.94 (s, 3H), 3.26 (s, 3H), 1.84 (s, 3H). **^{13}C{^{1}H} NMR** (126 MHz, $CDCl_3$), δ (ppm) = 172.4, 154.9, 147.5, 140.2, 139.9, 128.6, 118.7, 116.5, 107.2, 56.3, 36.7, 19.9. **FT-IR** (cm^{-1}, neat, ATR), ṽ = 1653, 1626, 1518, 1342, 1186, 1116, 1026, 1001, 892, 734, 555. **HRMS (ESI)** calcd for $C_{12}H_{15}N_2O_4$ $[M+H]^+$: 251.1026, found 251.1030.

***Ethyl 2-diazo-3-(1,3-dimethyl-2-oxoindolin-3-yl)propanoate* (3a)**

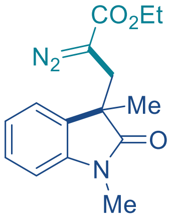

Prepared according to the *General Procedure* from the corresponding *N*-methyl-*N*-phenylmethacrylamide **1a** (105.1 mg, 0.60 mmol, 3.0 equiv), **2c** (93.2 mg, 0.20 mmol, 1.0 equiv) and Na_2CO_3 (20.0 mg, 0.2 mmol, 1.0 equiv). After purification by flash column chromatography (hexane/AcOEt 19:1 – 9:1), the title compound **3a** was obtained as a yellow oil (32.1 mg, 0.11 mmol, 56 %). **R_f** = 0.32 (silica gel, hexane/AcOEt, 4:1 (v/v)). **^{1}H NMR** (600 MHz, $CDCl_3$), δ (ppm) = 7.28 (dd, *J* = 7.7, 1.2 Hz, 1H), 7.24 (d, *J* = 7.4 Hz, 1H), 7.05 (td, *J* = 7.5, 1.0 Hz, 1H), 6.84 (d, *J* = 7.8 Hz, 1H), 4.07 (q, *J* = 7.1 Hz, 2H), 3.20 (s, 3H), 2.86 (d, *J* = 15.1 Hz, 1H), 2.75 (d, *J* = 15.2 Hz, 1H), 1.43 (s, 3H), 1.15 (t, *J* = 7.1 Hz, 3H). **^{13}C{^{1}H} NMR** (151 MHz, $CDCl_3$), δ (ppm) = 179.5, 173.5, 143.3, 132.5, 128.5, 123.6, 122.7, 108.2, 61.0, 48.4, 31.5, 26.4, 22.5, 14.5 ($C=N_2$ was not observed). **FT-IR** (cm^{-1}, neat, ATR), ṽ = 2086, 1709, 1613, 1497, 1470, 1371, 1336, 1155, 1121, 757. **HRMS (ESI)** calcd for $C_{15}H_{17}N_3O_3Na$ $[M+Na]^+$: 310.1162, found 310.1164.

2,2,2-trichloroethyl 2-diazo-3-(1,3-dimethyl-2-oxoindolin-3-yl)propanoate **(3b)**

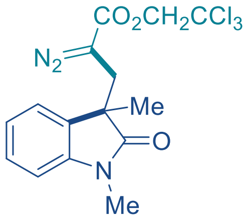

Prepared according to the *General Procedure B* from the corresponding *N*-methyl-*N*-phenylmethacrylamide **1a** (105.1 mg, 0.60 mmol, 3.0 equiv), **2g** (113.9 mg, 0.20 mmol, 1.0 equiv) and Na_2CO_3 (20.0 mg, 0.2 mmol, 1.0 equiv). After purification by flash column chromatography (silica gel, hexane/Et_2O 4:1), the title compound **3b** was obtained as a yellow oil (49.2 mg, 0.12 mmol, 63 %). $\mathbf{R_f}$ = 0.35 (silica gel, hexane/Et_2O, 4:1 (v/v)). **^{1}H NMR** (600 MHz, $CDCl_3$), δ (ppm) = 7.26 (d, *J* = 7.8 Hz, 2H), 7.05 (t, *J* = 7.5 Hz, 1H), 6.84 (d, *J* = 7.7 Hz, 1H), 4.75 (d, *J* = 11.9 Hz, 1H), 4.58 (d, *J* = 12.1 Hz, 1H), 3.21 (s, 3H), 2.93 (d, *J* = 15.2 Hz, 1H), 2.80 (d, *J* = 15.2 Hz, 1H), 1.45 (s, 3H). **^{13}C{^{1}H} NMR** (151 MHz, $CDCl_3$), δ (ppm) = 179.4, 143.4, 132.2, 128.6, 123.6, 122.9, 108.3, 95.3, 73.6, 48.3, 31.4, 26.4, 22.7 (C=N_2 and C=O from the ester were not observed). **FT-IR** (cm^{-1}, neat, ATR), ṽ = 2097, 1704, 1613, 1493, 1471, 1451, 1375, 1336, 1298, 1264, 1147, 1116, 1058, 1029, 830, 791, 752, 732, 716, 574. **HRMS (ESI)** calcd for $C_{15}H_{14}Cl_3N_3O_3Na$ $[M+Na]^+$: 411.9993, found 411.9996.

2,2,2-trichloroethyl 2-diazo-3-(5-(tert-butyl)-1,3-dimethyl-2-oxoindolin-3-yl)propanoate **(3c)**

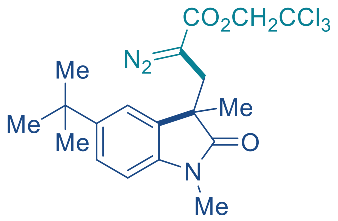

Prepared according to the *General Procedure* from the corresponding *N*-(4-*tert*-butylphenyl)-*N*-methylmethacrylamide **1b** (138.8 mg, 0.60 mmol, 3.0 equiv), **2g** (113.9 mg, 0.20 mmol, 1.0 equiv) and Na_2CO_3 (20.0 mg, 0.2 mmol, 1.0 equiv). After purification by flash column chromatography (silica gel, hexane/AcOEt 5:1) followed by preparative TLC (silica gel, cyclohexane/AcOEt 5:1), the title compound **3c** was obtained as a yellow oil (53.4 mg, 0.12 mmol, 60 %). $\mathbf{R_f}$ = 0.58 (silica gel, hexane/AcOEt, 3:2 (v/v)). **^{1}H NMR** (600 MHz, $CDCl_3$), δ (ppm) = 7.32 – 7.25 (m, 2H), 6.76 (d, *J* = 8.1 Hz, 1H), 4.74 (d, *J* = 12.0 Hz, 1H), 4.58 (d, *J* = 12.1 Hz, 1H), 3.19 (s, 3H), 2.91 (d, *J* = 15.2 Hz, 1H), 2.79 (d, *J* = 15.2 Hz, 1H), 1.45 (s, 3H), 1.31 (s, 9H). **^{13}C{^{1}H} NMR** (126 MHz, $CDCl_3$), δ (ppm) = 179.5, 173.9, 146.1, 140.9, 127.1, 126.0, 125.1, 107.7, 95.3, 73.6, 34.7, 31.7, 31.5, 29.8, 26.4, 22.6 (C=N_2 was not observed). **FT-IR** (cm^{-1}, neat, ATR), ṽ = 2964, 2100, 1708, 1499, 1375, 1265, 1126, 1112, 1057, 822, 733, 704, 576. **HRMS (ESI)** calcd for $C_{19}H_{22}Cl_3N_3O_3Na$ $[M+Na]^+$: 468.0619, found 468.0622.

***2,2,2-trichloroethyl 2-diazo-3-(5-methoxy-1,3-dimethyl-2-oxoindolin-3-yl)propanoate* (3d)**

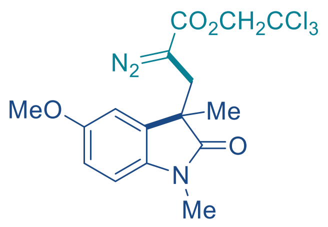

Prepared according to the *General Procedure B* from the corresponding *N*-(4-methoxyphenyl)-*N*-methylmethacrylamide **1c** (123.2 mg, 0.60 mmol, 3.0 equiv), **2g** (113.9 mg, 0.20 mmol, 1.0 equiv) and Na_2CO_3 (20.0 mg, 0.2 mmol, 1.0 equiv). After purification by flash column chromatography (silica gel, hexane/AcOEt 4:1 – 3:1), the title compound **3d** was obtained as a yellow oil (50.4 mg, 0.12 mmol, 60 %). $\mathbf{R_f}$ = 0.44 (silica gel, hexane/AcOEt, 3:2 (v/v)). **^{1}H NMR** (600 MHz, $CDCl_3$), δ (ppm) = 6.87 (d, J = 2.5 Hz, 1H), 6.78 (dd, J = 8.5, 2.5 Hz, 1H), 6.73 (d, J = 8.4 Hz, 1H), 4.78 (d, J = 12.0 Hz, 1H), 4.58 (d, J = 11.9 Hz, 1H), 3.78 (s, 3H), 3.18 (s, 3H), 2.92 (d, J = 15.2 Hz, 1H), 2.77 (d, J = 15.2 Hz, 1H), 1.43 (s, 3H). **^{13}C{^{1}H} NMR** (151 MHz, $CDCl_3$), δ (ppm) = 179.0, 156.3, 136.8, 133.5, 113.1, 111.0, 108.6, 73.5, 56.0, 48.7, 31.3, 26.5, 22.7 (CCl_3, C=N_2 and ester C=O were not observed). **FT-IR** (cm^{-1}, neat, ATR), ṽ = 2096, 1698, 1600, 1497, 1470, 1434, 1375, 1336, 1288, 1228, 1108, 1038, 909, 828, 794, 727, 574, 556. **HRMS (ESI)** calcd for $C_{16}H_{16}Cl_3N_3O_4Na$ $[M+Na]^+$: 442.0099, found 442.0101.

***2,2,2-trichloroethyl 2-diazo-3-(5-fluoro-1,3-dimethyl-2-oxoindolin-3-yl)propanoate* (3e)**

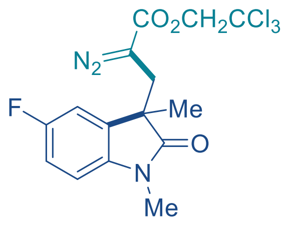

Prepared according to the *General Procedure* from the corresponding *N*-(4-fluoroyphenyl)-*N*-methylmethacrylamide **1d** (115.9 mg, 0.60 mmol, 3.0 equiv), **2g** (113.9 mg, 0.20 mmol, 1.0 equiv) and Na_2CO_3 (20.0 mg, 0.2 mmol, 1.0 equiv). After purification by flash column chromatography (silica gel, cyclohexane/AcOEt 4:1), the title compound **3e** was obtained as a yellow oil (55.9 mg, 0.14 mmol, 68 %). $\mathbf{R_f}$ = 0.57 (silica gel, cyclohexane/AcOEt, 3:2 (v/v)). **^{1}H NMR** (500 MHz, $CDCl_3$), δ (ppm) = 7.02 (dd, J = 7.8, 2.6 Hz, 1H), 6.97 (td, J = 8.8, 2.6 Hz, 1H), 6.76 (dd, J = 8.5, 4.1 Hz, 1H), 4.77 (d, J = 12.0 Hz, 1H), 4.60 (d, J = 11.6 Hz, 1H), 3.20 (s, 3H), 2.94 (d, J = 15.3 Hz, 1H), 2.79 (d, J = 15.1 Hz, 1H), 1.44 (s, 3H). **^{13}C{^{1}H} NMR** (126 MHz, $CDCl_3$), δ (ppm) = 179.0, 160.5, 158.5 (d, J = 241.3 Hz), 139.3, 133.9 (d, J = 7.9 Hz), 114.8 (d, J = 23.4 Hz), 112.0 (d, J = 24.8 Hz), 95.3, 73.6, 48.8, 31.3, 26.6, 22.7 (C=N_2 and ester C=O were not observed). **^{19}F{^{1}H} NMR** (377 MHz, $CDCl_3$), δ (ppm) = -200.0. **FT-IR** (cm^{-1}, neat, ATR), ṽ = 2097, 1702, 1609, 1493,

1469, 1443, 1375, 1335, 1269, 1223, 1142, 1115, 1053, 809, 715, 560. **HRMS (ESI)** calcd for $C_{15}H_{13}Cl_3FN_3O_3Na$ $[M+Na]^+$: 429.9899, found 429.9902.

2,2,2-trichloroethyl 2-diazo-3-(5-chloro-1,3-dimethyl-2-oxoindolin-3-yl)propanoate **(3f)**

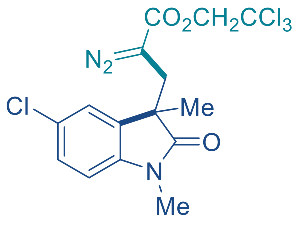

Prepared according to the *General Procedure* from the corresponding *N*-(4-chloroyphenyl)-*N*-methylmethacrylamide **1e** (123.2 mg, 0.60 mmol, 3.0 equiv), **2g** (113.9 mg, 0.20 mmol, 1.0 equiv) and Na_2CO_3 (20.0 mg, 0.2 mmol, 1.0 equiv). After purification by flash column chromatography (silica gel, hexane/AcOEt 4:1), the title compound **3f** was obtained as a yellow oil (60.7 mg, 0.14 mmol, 71 %). $\mathbf{R_f}$ = 0.58 (silica gel, hexane/AcOEt, 3:2 (v/v)). **^{1}H NMR** (600 MHz, $CDCl_3$), δ (ppm) = 7.30 – 7.19 (m, 2H), 6.78 (d, *J* = 8.9 Hz, 1H), 4.81 (d, *J* = 12.1 Hz, 1H), 4.64 (br s, 1H), 3.22 (s, 3H), 2.95 (d, *J* = 15.2 Hz, 1H), 2.81 (d, *J* = 15.7 Hz, 1H), 1.46 (s, 3H). **^{13}C{^{1}H} NMR** (151 MHz, $CDCl_3$), δ (ppm) = 178.9, 165.0, 141.9, 133.9, 128.5, 128.3, 124.3, 109.2, 95.3, 73.6, 48.6, 31.3, 26.6, 22.6 ($C=N_2$ was not observed). ^{1}H-^{1}H COSY, ^{1}H-^{13}C HSQC and ^{1}H-^{13}C HMBC experiments were performed. **FT-IR** (cm^{-1}, neat, ATR), ṽ = 2096, 1704, 1610, 1489, 1375, 1335, 1149, 1124, 1055, 824, 717, 575, 546. **HRMS (ESI)** calcd for $C_{15}H_{13}Cl_4N_3O_3Na$ $[M+Na]^+$: 445.9603, found 445.9605.

2,2,2-trichloroethyl 2-diazo-3-(5-bromo-1,3-dimethyl-2-oxoindolin-3-yl)propanoate **(3g)**

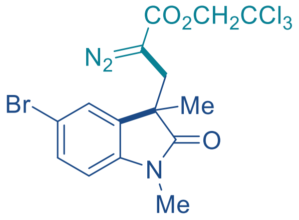

Prepared according to the *General Procedure* from the corresponding *N*-(4-bromophenyl)-*N*-methylmethacrylamide **1f** (152.5 mg, 0.60 mmol, 3.0 equiv), **2g** (113.9 mg, 0.20 mmol, 1.0 equiv) and Na_2CO_3 (20.0 mg, 0.2 mmol, 1.0 equiv). After purification by flash column chromatography (silica gel, hexane/AcOEt 5:1), the title compound **3g** was obtained as a yellow oil (62.6 mg, 0.13 mmol, 67 %). $\mathbf{R_f}$ = 0.26 (silica gel, hexane/AcOEt, 4:1 (v/v)). **^{1}H NMR** (500 MHz, $CDCl_3$), δ (ppm) = 7.39 (d, *J* = 8.1 Hz, 1H), 7.37 (d, *J* = 2.0 Hz, 1H), 6.72 (d, *J* = 8.4 Hz, 1H), 4.80 (d, *J* = 12.1 Hz, 1H), 4.60 (br s, 1H), 3.19 (s, 3H), 2.92 (d, *J* = 14.9 Hz, 1H), 2.78 (d, *J* = 14.7 Hz, 1H), 1.44 (s, 3H). **^{13}C{^{1}H} NMR** (151 MHz, $CDCl_3$), δ (ppm) = 178.8, 165.0, 142.4, 134.3, 131.5, 127.0, 115.5, 109.8, 95.3, 73.6, 48.6, 31.3, 26.6, 22.6 ($C=N_2$ was not observed). **FT-IR** (cm^{-1},

neat, ATR), ṽ = 2924, 2097, 1712, 1650, 1625, 1487, 1451, 1423, 1362, 1114, 1069, 1010, 916, 831, 811, 716, 574. **HRMS (ESI)** calcd for $C_{15}H_{13}BrCl_3N_3O_3Na$ $[M+Na]^+$: 489.9098, found 489.9099.

2,2,2-trichloroethyl 2-diazo-3-(5-cyano-1,3-dimethyl-2-oxoindolin-3-yl)propanoate **(3h)**

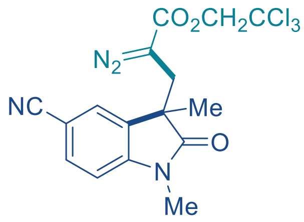

Prepared according to the *General Procedure* from the corresponding *N*-(4-cyanophenyl)-*N*-methylmethacrylamide **1g** (120.1 mg, 0.60 mmol, 3.0 equiv), **2g** (113.9 mg, 0.20 mmol, 1.0 equiv) and Na_2CO_3 (20.0 mg, 0.2 mmol, 1.0 equiv). After purification by flash column chromatography (silica gel, hexane/AcOEt 5:1), followed by preparative TLC (silica gel, cyclohexane/AcOEt 5:1), the title compound **3h** was obtained as a yellow oil (45.7 mg, 0.11 mmol, 55 %). $\mathbf{R_f}$ = 0.22 (silica gel, hexane/AcOEt, 4:1 (v/v)). ^{1}**H NMR** (500 MHz, $CDCl_3$), δ (ppm) = 7.66 – 7.58 (m, 1H), 7.52 (d, *J* = 1.8 Hz, 1H), 6.91 (d, *J* = 8.2 Hz, 1H), 4.76 (d, *J* = 11.9 Hz, 1H), 4.61 (d, *J* = 11.9 Hz, 1H), 3.25 (s, 3H), 2.95 (d, *J* = 15.3 Hz, 1H), 2.83 (d, *J* = 15.2 Hz, 1H), 1.47 (s, 3H). ^{13}**C{^{1}H} NMR** (126 MHz, $CDCl_3$), δ (ppm) = 179.1, 165.6, 147.2, 134.0, 133.3, 127.2, 119.1, 108.8, 106.2, 95.2, 73.7, 48.3, 29.8, 26.7, 22.6 ($C=N_2$ was not observed). **FT-IR** (cm^{-1}, neat, ATR), ṽ = 2101, 1715, 1633, 1500, 1424, 1362, 1111, 1012, 812, 722, 574. **HRMS (ESI)** calcd for $C_{16}H_{13}Cl_3N_4O_3Na$ $[M+Na]^+$: 436.9945, found 436.9946.

2,2,2-trichloroethyl 2-diazo-3-(4,6-dichloro-1,3-dimethyl-2-oxoindolin-3-yl)propanoate **(3i)**

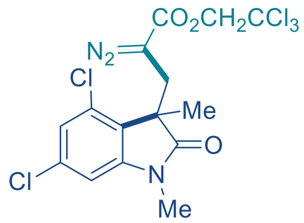

Prepared according to the *General Procedure* from the corresponding *N*-(3,5-dichlorophenyl)-*N*-methylmethacrylamide **1h** (146.4 mg, 0.60 mmol, 3.0 equiv), **2g** (113.9 mg, 0.20 mmol, 1.0 equiv) and Na_2CO_3 (20.0 mg, 0.2 mmol, 1.0 equiv) and 4DPAIPN (1.6 mg, 0.002 mmol, 0.01 equiv). After purification by flash column chromatography (silica gel, hexane/AcOEt 5:1), followed by preparative TLC (silica gel, DCM 100%), the title compound **3i** was obtained as a yellow oil (63.6 mg, 0.13 mmol, 68 %). $\mathbf{R_f}$ = 0.69 (silica gel, hexane/AcOEt, 3:2 (v/v)). ^{1}**H NMR** (600 MHz, $CDCl_3$), δ (ppm) = 7.02 (d, *J* = 1.8 Hz, 1H), 6.74 (d, *J* = 1.8 Hz, 1H), 4.75 (br s, 1H), 4.60 (d, *J* = 12.0 Hz, 1H), 3.18 (s, 3H), 3.15 (d, *J* = 16.1 Hz, 1H), 3.05 (d, *J* = 15.6 Hz, 1H), 1.59 (s, 3H). ^{13}**C{^{1}H} NMR** (151 MHz, $CDCl_3$), δ (ppm) = 178.6, 146.1, 135.3, 131.9, 126.8, 123.4,

107.7, 95.4, 73.5, 49.5, 29.2, 26.8, 20.3 (C=N_2 and ester C=O were not observed). **FT-IR** (cm^{-1}, neat, ATR), ṽ = 2104, 1724, 1605, 1581, 1437, 1376, 1349, 1142, 1113, 1056, 834. **HRMS (ESI)** calcd for $C_{15}H_{12}Cl_5NO_3$ $[M+Na–N_2]^+$: 429.9333, found 429.9340.

***2,2,2-trichloroethyl 2-diazo-3-(7-methoxy-1,3-dimethyl-5-nitro-2-oxoindolin-3-yl)propanoate* (3j)**

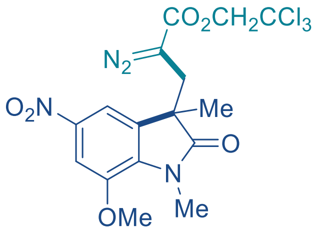

Prepared according to the *General Procedure* from the corresponding *N*-(2-methoxy-4-nitrophenyl)-*N*-methylmethacrylamide **1i** (150.1 mg, 0.60 mmol, 3.0 equiv), **2g** (113.9 mg, 0.20 mmol, 1.0 equiv) and Na_2CO_3 (20.0 mg, 0.2 mmol, 1.0 equiv) and 4DPAIPN (1.6 mg, 0.002 mmol, 0.01 equiv). After purification by flash column chromatography (silica gel, hexane/AcOEt 4:1), the title compound **3j** was obtained as a yellow oil (36.7 mg, 0.08 mmol, 40 %). R_f = 0.50 (silica gel, hexane/AcOEt, 3:2 (v/v)). **^{1}H NMR** (400 MHz, $CDCl_3$), δ (ppm) = 7.83 (d, *J* = 2.0 Hz, 1H), 7.76 (d, *J* = 2.1 Hz, 1H), 4.75 (d, *J* = 13.0 Hz, 1H), 4.60 (br s, 1H), 3.96 (s, 3H), 3.52 (s, 3H), 2.95 (d, *J* = 15.3 Hz, 1H), 2.83 (d, *J* = 15.3 Hz, 1H), 1.47 (s, 3H). **^{13}C{^{1}H} NMR** (101 MHz, $CDCl_3$), δ (ppm) = 179.7, 164.6, 144.8, 143.6, 137.4, 133.7, 112.6, 108.3, 95.1, 73.6, 56.4, 48.4, 31.6, 29.8, 22.5 (C=N_2 was not observed). **FT-IR** (cm^{-1}, neat, ATR), ṽ = 2926, 2104, 1723, 1527, 1333, 1264, 733, 706. **HRMS (ESI)** calcd for $C_{16}H_{15}Cl_3N_4O_6Na$ $[M+Na]^+$: 486.9949, found 486.9957.

***2,2,2-trichloroethyl 2-diazo-3-(7-chloro-1,3-dimethyl-2-oxoindolin-3-yl)propanoate* (3k)**

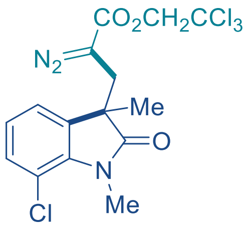

Prepared according to the *General Procedure* from the corresponding *N*-(2-chlorophenyl)-*N*-methylmethacrylamide **1j** (125.8 mg, 0.60 mmol, 3.0 equiv), **2g** (113.9 mg, 0.20 mmol, 1.0 equiv) and Na_2CO_3 (20.0 mg, 0.2 mmol, 1.0 equiv). After purification by flash column chromatography (silica gel, hexane/AcOEt 5:1), followed by preparative TLC (silica gel, cyclohexane/AcOEt 4:1), the title compound **3k** was obtained as a yellow oil (28.4 mg, 0.06 mmol, 35 %). R_f = 0.25 (silica gel, cyclohexane/AcOEt, 4:1 (v/v)). **^{1}H NMR** (500 MHz, $CDCl_3$), δ (ppm) = 6.38 (dd, *J* = 9.6, 6.2 Hz, 1H), 6.32 (d, *J* = 6.1 Hz, 1H), 6.11 (d, *J* = 9.6 Hz, 1H), 4.95 (d, *J* = 11.8 Hz, 1H), 4.88 (d, J = 11.8 Hz, 1H), 2.98 (s, 3H), 2.53 (d, *J* = 12.8 Hz, 1H), 2.06 (d, *J* =

12.9 Hz, 1H), 1.37 (s, 3H). **^{13}C{^{1}H} NMR** (151 MHz, $CDCl_3$), δ (ppm) = 176.6, 162.5, 130.8, 129.5, 129.0, 123.3, 118.3, 103.7, 89.5, 74.6, 46.5, 29.9, 27.0, 16.2 (C=N_2 was not observed). **FT-IR** (cm^{-1}, neat, ATR), ṽ = 2101, 1716, 1604, 1570, 1373, 1144, 1110, 1056, 832, 754. **HRMS (ESI)** calcd for $C_{15}H_{13}Cl_4N_3O_3Na$ $[M+Na]^+$: 445.9603, found 445.9603.

2,2,2-trichloroethyl 2-diazo-3-(1-(2-ethoxy-oxoethyl)-3-methyl-2-oxoindolin-3-yl)propanoate **(3l)**

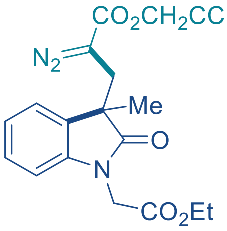

Prepared according to the *General Procedure* from the corresponding ethyl *N*-methacryloyl-*N*-phenylglycinate **1k** (148.4 mg, 0.60 mmol, 3.0 equiv), **2g** (113.9 mg, 0.20 mmol, 1.0 equiv) and Na_2CO_3 (20.0 mg, 0.2 mmol, 1.0 equiv). After purification by flash column chromatography (silica gel, DCM 100%) followed by preparative TLC (silica gel, hexane/AcOEt 4:1), the title compound **3l** was obtained as a yellow oil (50.8 mg, 0.11 mmol, 55 %). **R_f** = 0.33 (silica gel, DCM 100 %). **^{1}H NMR** (600 MHz, $CDCl_3$), δ (ppm) = 7.29 (d, *J* = 7.4 Hz, 1H), 7.24 (t, *J* = 7.7 Hz, 1H), 7.07 (t, *J* = 7.5 Hz, 1H), 6.72 (d, *J* = 7.8, 1H), 4.78 (d, *J* = 12.0 Hz, 1H), 4.59 (d, *J* = 12.0 Hz, 1H), 4.53 (d, *J* = 17.5 Hz, 1H), 4.37 (d, *J* = 17.5 Hz, 1H), 4.22 – 4.17 (m, 2H), 2.96 (d, *J* = 15.3 Hz, 1H), 2.85 (d, *J* = 14.7 Hz, 1H), 1.48 (s, 3H), 1.25 (t, *J* = 7.1 Hz, 3H). **^{13}C{^{1}H} NMR** (151 MHz, $CDCl_3$), δ (ppm) = 179.5, 169.9, 167.4, 142.1, 132.0, 128.6, 123.9, 123.2, 108.3, 95.3, 73.6, 68.3, 61.9, 48.4, 41.5, 31.5, 14.3 (C=N_2 was not observed). **FT-IR** (cm^{-1}, neat, ATR), ṽ = 2102, 1718, 1616, 1493, 1467, 1377, 1343, 1206, 1123, 720. **HRMS (ESI)** calcd for $C_{18}H_{18}Cl_3N_3O_5Na$ $[M+Na]^+$: 484.0204, found 484.0196.

2,2,2-trichloroethyl 3-(3-benzyl-1-methyl-2-oxoindolin-3-yl)-2-diazopropanoate **(3m)**

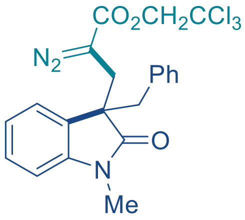

Prepared according to the *General Procedure* from the corresponding 2-benzyl-*N*-methyl-*N*-phenylacrylamide **1m** (150.8 mg, 0.60 mmol, 3.0 equiv), **2g** (113.9 mg, 0.20 mmol, 1.0 equiv) and Na_2CO_3 (20.0 mg, 0.2 mmol, 1.0 equiv). After purification by flash column chromatography (silica gel, cyclohexane/AcOEt 19:1) followed by preparative TLC (silica gel, cyclohexane/AcOEt 4:1), the title compound **3m** was obtained as a pale-yellow oil (57.6 mg, 0.12 mmol, 62 %). **R_f** = 0.28 (silica gel, cyclohexane/AcOEt, 4:1 (v/v)). **^{1}H NMR** (400 MHz, $CDCl_3$), δ (ppm) = 7.26 – 7.24 (m, 1H), 7.17 (t, *J* = 7.8 Hz, 1H), 7.10 – 6.99 (m, 4H), 6.82 (d, *J* = 6.8 Hz, 2H),

6.58 (d, *J* = 7.7 Hz, 1H), 4.76 (d, *J* = 12.0 Hz, 1H), 4.57 (d, *J* = 12.0 Hz, 1H), 3.54 – 3.20 (m, 2H), 3.15 – 3.00 (m, 2H), 2.96 (s, 3H). **^{13}C{^{1}H} NMR** (151 MHz, $CDCl_3$), δ (ppm) = 177.8, 165.0, 143.8, 135.1, 129.8 (2C), 129.5, 128.6, 127.6 (2C), 126.7, 124.5, 122.3, 107.9, 95.2, 73.5, 54.3, 43.0, 29.9, 26.0 (C=N_2 was not observed). **FT-IR** (cm^{-1}, neat, ATR), ṽ = 2100, 1707, 1613, 1502, 1376, 1130, 1116, 717, 700. **HRMS (ESI)** calcd for $C_{21}H_{18}Cl_3N_3O_3Na$ $[M+Na]^+$: 488.0306, found 488.0315.

***2,2,2-trichloroethyl 2-diazo-3-(1-methyl-2-oxo-1,2,5,6-tetrahydro-4-H-pyrrolo[3,2,1-ij]quinolin-1-yl)propanoate* (3n)**

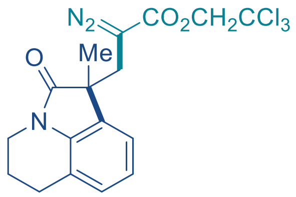

Prepared according to the *General Procedure* from the corresponding 1-(3,4-dihydroquinolin-1(2*H*)-yl)-2-methylprop-2-en-1-one **1l** (120.8 mg, 0.60 mmol, 3.0 equiv), **2g** (113.9 mg, 0.20 mmol, 1.0 equiv) and Na_2CO_3 (20.0 mg, 0.2 mmol, 1.0 equiv). After purification by flash column chromatography (silica gel, hexane/AcOEt 3:1 – 3:2) followed by preparative TLC (silica gel, hexane/AcOEt 4:1), the title compound **3n** was obtained as a yellow oil (36.7 mg, 0.09 mmol, 44 %). **R$_f$** = 0.38 (silica gel, hexane/AcOEt, 3:2 (v/v)). **^{1}H NMR** (600 MHz, $CDCl_3$), δ (ppm) = 7.08 (d, *J* = 7.3 Hz, 1H), 7.00 (d, *J* = 7.7 Hz, 1H), 6.93 (t, *J* = 7.5 Hz, 1H), 4.78 (d, *J* = 12.0 Hz, 1H), 4.56 (d, *J* = 11.9 Hz, 1H), 3.75 – 3.60 (m, 2H), 2.91 (d, *J* = 15.2 Hz, 1H), 2.83 – 2.71 (m, 3H), 2.05 – 1.95 (m, 2H), 1.45 (s, 3H). **^{13}C{^{1}H} NMR** (151 MHz, $CDCl_3$), δ (ppm) = 178.1, 161.6, 139.1, 130.8, 127.4, 122.3, 121.5, 120.4, 73.5, 49.7, 39.1, 31.2, 24.7, 22.3, 21.3 (C=N_2 and CCl_3 were not observed). ^{1}H-^{1}H COSY, ^{1}H-^{13}C HSQC and ^{1}H-^{13}C HMBC experiment were performed. **FT-IR** (cm^{-1}, neat, ATR), ṽ = 2927, 2094, 1701, 1627, 1481, 1449, 1355, 1338, 1265, 1241, 1120, 1055, 825, 790, 730, 714, 571. **HRMS (ESI)** calcd for $C_{17}H_{16}Cl_3N_3O_3Na$ $[M+Na]^+$: 438.0149, found 438.0158.

***2,2,2-trichloroethyl 2-(methyl(phenyl)carbamoyl)-1-((1-methyl-2-oxoindolin-3-yl)methyl)cyclopropane-1-carboxylate* (3o)**

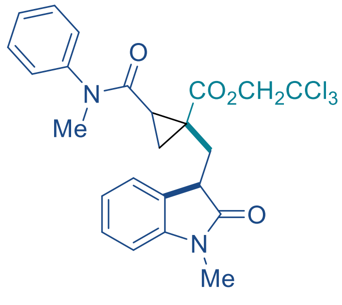

Prepared according to the *General Procedure* from the corresponding *N*-methyl-*N*-phenylacrylamide **1n** (96.8 mg, 0.60 mmol, 1.5 equiv), **2g** (113.9 mg, 0.20 mmol, 1.0 equiv), Na_2CO_3 (20.0 mg, 0.2 mmol, 1.0 equiv) and 4DPAIPN (1.6 mg, 0.002 mmol, 0.01 equiv). After purification by flash column chromatography (silica gel, cyclohexane/AcOEt 3:1), the title compound **3o** was obtained as a colorless oil (45.3 mg, 0.09 mmol, 45 %). **R_f** = 0.12 (silica gel, cyclohexane/AcOEt, 4:1 (v/v)). **^{1}H NMR** (400 MHz, $CDCl_3$), δ (ppm) = 7.44 (t, *J* = 7.6 Hz, 2H), 7.42 – 7.35 (m, 2H), 7.34 – 7.27 (m, 1H), 7.17 (d, *J* = 7.5 Hz, 2H), 7.10 (t, *J* = 7.6 Hz, 1H), 7.00 (d, *J* = 8.1 Hz, 1H), 4.63 (d, *J* = 11.9 Hz, 1H), 4.51 (d, *J* = 11.9 Hz, 1H), 3.38 (s, 3H), 3.20 (s, 3H), 3.13 (dd, *J* = 10.6, 7.7 Hz, 1H), 2.95 – 2.80 (m, 2H), 2.74 (dd, *J* = 13.4, 9.4 Hz, 1H), 2.20 – 2.08 (m, 2H). **^{13}C{^{1}H} NMR** (151 MHz, $CDCl_3$), δ (ppm) = 173.3, 172.0, 168.4, 143.7, 138.7, 130.2 (2C), 129.3, 129.0, 128.3, 127.5 (2C), 123.6, 123.5, 115.1, 94.6, 74.3, 54.7, 48.9, 41.2, 40.2, 38.0, 35.9, 29.8. **FT-IR** (cm^{-1}, neat, ATR), ṽ = 2927, 1741, 1657, 1596, 1496, 1470, 1420, 1369, 1269, 1216, 1137, 755, 703, 572. **HRMS (ESI)** calcd for $C_{24}H_{24}Cl_3N_2O_4$ $[M+H]^+$: 509.0796, found 509.0798.

2,2,2-trifluoroethyl 2-diazo-3-(1,3-dimethyl-2-oxoindolin-3-yl)propanoate **(3p)**

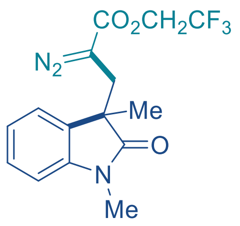

Prepared according to the *General Procedure* from the corresponding *N*-methyl-*N*-phenylmethacrylamide **1a** (105.1 mg, 0.60 mmol, 3.0 equiv), **2h** (104.0 mg, 0.20 mmol, 1.0 equiv) and Na_2CO_3 (20.0 mg, 0.2 mmol, 1.0 equiv). After purification by flash column chromatography (hexane/AcOEt 4:1), the title compound **3p** was obtained as a yellow oil (44.1 mg, 0.13 mmol, 65 %). **R_f** = 0.10 (silica gel, hexane/AcOEt, 4:1 (v/v)). **^{1}H NMR** (500 MHz, CD_3CN), δ (ppm) = 7.32 – 7.29 (m, 2H), 7.05 (t, *J* = 7.5 Hz, 1H), 6.94 (d, *J* = 7.8 Hz, 1H), 4.52 (q, *J* = 8.6 Hz, 2H), 3.13 (s, 3H), 2.81 (d, *J* = 15.3 Hz, 1H), 2.77 (d, *J* = 15.2 Hz, 1H), 1.36 (s, 3H). **^{13}C{^{1}H} NMR** (126 MHz, CD_3CN), δ (ppm) = 178.8, 143.7, 132.3, 128.5, 123.3, 122.3, 108.3, 59.9 (q, *J* = 36.3 Hz), 47.8, 30.9, 25.6, 21.5 ($C=N_2$, C=O and CF_3 were not observed). **^{19}F{^{1}H} NMR** (377 MHz, CD_3CN), δ (ppm) = -75.0. **FT-IR** (cm^{-1}, neat, ATR), ṽ = 2100, 1710, 1651, 1613, 1595, 1493, 1419, 1345, 1275, 1150, 1116, 972, 914, 753, 699, 658, 556, 486. **HRMS (ESI)** calcd for $C_{15}H_{14}F_3N_3O_3Na$ $[M+Na]^+$: 364.0879, found 364.0883.

3-(2-diazo-3,3,3-trifluoropropyl)-1,3-dimethylindolin-2-one **(3q)**

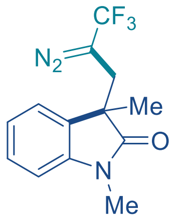

Prepared according to the *General Procedure* from the corresponding *N*-methyl-*N*-phenylmethacrylamide **1a** (105.1 mg, 0.60 mmol, 3.0 equiv), **2i** (92.4 mg, 0.20 mmol, 1.0 equiv) and Na_2CO_3 (20.0 mg, 0.2 mmol, 1.0 equiv). After purification by flash column chromatography (hexane/AcOEt 9:1), the title compound **3q** was obtained as a yellow oil (34.5 mg, 0.12 mmol, 61%). **R_f** = 0.21 (silica gel, hexane/AcOEt, 4:1 (v/v)). **^{1}H NMR** (600 MHz, $CDCl_3$), δ (ppm) = 7.32 (td, *J* = 7.7, 1.2 Hz, 1H), 7.19 (d, *J* = 7.3 Hz, 1H), 7.08 (t, *J* = 7.5 Hz, 1H), 6.88 (d, *J* = 7.8 Hz, 1H), 3.22 (s, 3H), 2.77 (d, *J* = 15.6 Hz, 1H), 2.63 (d, *J* = 15.6 Hz, 1H), 1.44 (s, 3H). **^{13}C{^{1}H} NMR** (151 MHz, $CDCl_3$), δ (ppm) = 179.0, 143.6, 131.9, 128.9, 126.6 (q, *J* = 268.4 Hz), 123.1, 122.9, 108.5, 48.3, 31.0, 26.4, 22.9 (C=N_2 was not observed). **^{19}F{^{1}H} NMR** (282 MHz, $CDCl_3$), δ (ppm) = -58.0. **FT-IR** (cm^{-1}, neat, ATR), ṽ = 2089, 1710, 1613, 1493, 1471, 1453, 1350, 1303, 1154, 1095, 752, 699, 470. **HRMS (ESI)** calcd for $C_{13}H_{12}F_3N_3ONa$ $[M+Na]^+$: 306.0825, found 306.0827.

3-(2-diazo-3-oxo-3-phenylpropyl)-1,3-dimethylindolin-2-one **(3r)**

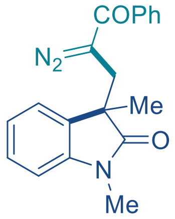

Prepared according to the *General Procedure* from the corresponding *N*-methyl-*N*-phenylmethacrylamide **1a** (105.1 mg, 0.60 mmol, 3.0 equiv), **2j** (100.0 mg, 0.20 mmol, 1.0 equiv) and Na_2CO_3 (20.0 mg, 0.2 mmol, 1.0 equiv). After purification by flash column chromatography (hexane/AcOEt 4:1 – 3:1), the title compound **3r** was obtained as a yellow oil (29.3 mg, 0.09 mmol, 46%). **R_f** = 0.12 (silica gel, hexane/AcOEt, 4:1 (v/v)). **^{1}H NMR** (500 MHz, $CDCl_3$), δ (ppm) = 7.46 – 7.40 (m, 1H), 7.40 – 7.32 (m, 2H), 7.32 – 7.20 (m, 4H), 7.12 – 7.02 (m, 1H), 6.94 – 6.84 (m, 1H), 3.23 (s, 3H), 3.11 (d, *J* = 14.8 Hz, 1H), 3.01 (d, *J* = 14.9 Hz, 1H), 1.50 (s, 3H). **^{13}C{^{1}H} NMR** (126 MHz, $CDCl_3$), δ (ppm) = 189.3, 179.6, 143.2, 137.4, 132.5, 131.4, 128.6 (2C), 128.6, 127.0 (2C), 123.7, 123.0, 108.1, 48.3, 31.4, 26.4, 22.7 (C=N_2 was not observed). **FT-IR** (cm^{-1}, neat, ATR), ṽ = 2074, 1708, 1613, 1493, 1470, 1448, 1377, 1340, 1261, 1125, 1097, 1027, 909, 701, 647, 541, 476. **HRMS (ESI)** calcd for $C_{19}H_{17}N_3O_2Na$ $[M+Na]^+$: 342.1213, found 342.1215.

2-diazo-3-(1,3-dimethy-2-oxolindolin-3-yl)propanenitrile **(3s)**

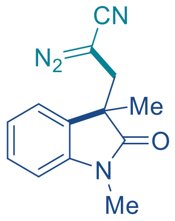

Prepared according to the *General Procedure* from the corresponding *N*-methyl-*N*-phenylmethacrylamide **1a** (105.1 mg, 0.60 mmol, 3.0 equiv), **2k** (83.8 mg, 0.20 mmol, 1.0 equiv) and Na_2CO_3 (20.0 mg, 0.2 mmol, 1.0 equiv). After purification by flash column chromatography (hexane/AcOEt 4:1 – 3:1), the title compound **3s** was obtained as an orange oil (15.4 mg, 0.06 mmol, 32%). $\mathbf{R_f}$ = 0.10 (silica gel, hexane/AcOEt, 4:1 (v/v)). **^{1}H NMR** (500 MHz, $CDCl_3$), δ (ppm) = 7.36 (t, J = 7.8 Hz, 1H), 7.23 (d, J = 7.5 Hz, 1H), 7.12 (t, J = 7.6 Hz, 1H), 6.92 (d, J = 7.8 Hz, 1H), 3.26 (s, 3H), 2.75 (d, J = 15.2 Hz, 1H), 2.61 (d, J = 15.3 Hz, 1H), 1.46 (s, 3H). **^{13}C{^{1}H} NMR** (126 MHz, $CDCl_3$), δ (ppm) = 178.7, 143.6, 131.3, 129.3, 123.3, 123.1, 113.3, 108.8, 48.4, 34.0, 26.6, 22.5 (C=N_2 was not observed). **FT-IR** (cm^{-1}, neat, ATR), ṽ = 2080, 1710, 1650, 1613, 1493, 1470, 1452, 1422, 1378, 1350, 1256, 1125, 1097, 1061, 1028, 914, 754, 699, 580, 542, 512, 487. **HRMS (ESI)** calcd for $C_{13}H_{12}N_4ONa$ $[M+Na]^+$: 263.0903, found 263.0905.

***Ethyl 1-diazo-2-(1,3-dimethyl-2-oxoindolin-3-yl)ethane-1-sulfonate* (3t)**

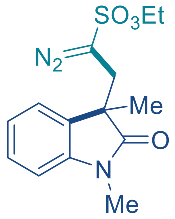

Prepared according to the *General Procedure* from the corresponding *N*-methyl-*N*-phenylmethacrylamide **1a** (105.1 mg, 0.60 mmol, 3.0 equiv), **2l** (100.4 mg, 0.20 mmol, 1.0 equiv) and Na_2CO_3 (20.0 mg, 0.2 mmol, 1.0 equiv). After purification by flash column chromatography (hexane/AcOEt 4:1 – 3:1), the title compound **3t** was obtained as an orange oil (55.2 mg, 0.17 mmol, 85 %). $\mathbf{R_f}$ = 0.53 (silica gel, hexane/AcOEt, 3:2 (v/v)). **^{1}H NMR** (500 MHz, $CDCl_3$), δ (ppm) = 7.33 (t, J = 7.7 Hz, 1H), 7.25 (d, J = 7.5 Hz, 1H), 7.11 (t, J = 7.5 Hz, 1H), 6.88 (d, J = 7.8 Hz, 1H), 3.98 (dq, J = 9.2, 7.1 Hz, 1H), 3.84 (dq, J = 9.3, 7.1 Hz, 1H), 3.22 (s, 3H), 2.99 (d, J = 15.6 Hz, 1H), 2.79 (d, J = 15.6 Hz, 1H), 1.46 (s, 3H), 1.27 (t, J = 7.2 Hz, 3H). **^{13}C{^{1}H} NMR** (126 MHz, $CDCl_3$), δ (ppm) = 178.8, 143.5, 131.8, 129.0, 123.5, 123.1, 108.5, 67.0, 47.9, 32.3, 26.5, 23.3, 14.8 (C=N_2 was not observed). **FT-IR** (cm^{-1}, neat, ATR), ṽ = 2971, 2929, 2087, 1708, 1612,1493, 1471, 1452, 1422, 1355, 1253, 1173, 1158, 1124, 1102, 1078, 1003, 917, 795, 754, 698, 637, 610, 578, 564, 487, 458. **HRMS (ESI)** calcd for $C_{14}H_{17}N_3O_4SNa$ $[M+Na]^+$: 346.0837, found 346.0840.

***2,2,2-trichloroethyl 2-diazo-3-(4-methyl-5-oxo-1,3-diphenyl-4,5-dihydro-1H-pyrazol-4-yl)propanoate* (3u)**

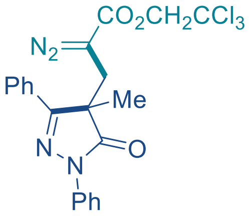

Prepared according to the *General Procedure* from the corresponding *N'*-benzylidene-*N*-phenylmethacrylohydrazide **1o** (158.4 mg, 0.60 mmol, 3.0 equiv), **2g** (113.9 mg, 0.20 mmol, 1.0 equiv), Na_2CO_3 (20.0 mg, 0.2 mmol, 1.0 equiv) and 4DPAIPN (1.6 mg, 0.002 mmol, 0.01 equiv). After purification by flash column chromatography (silica gel, hexane/AcOEt 5:1), the title compound **3u** was obtained as a yellow oil (48.8 mg, 0.10 mmol, 51%). **R_f** = 0.31 (silica gel, hexane/AcOEt, 4:1 (v/v)). **^{1}H NMR** (500 MHz, $CDCl_3$), δ (ppm) = 8.00 (d, J = 8.1 Hz, 2H), 7.97 – 7.90 (m, 2H), 7.51 – 7.40 (m, 5H), 7.27 – 7.13 (m, 1H), 4.67 (br s, 2H), 3.19 (d, J = 15.5 Hz, 1H), 3.04 (d, J = 15.6 Hz, 1H), 1.72 (s, 3H). **^{13}C{^{1}H} NMR** (126 MHz, $CDCl_3$), δ (ppm) = 175.7, 164.8, 160.3, 137.9, 130.8, 130.5, 129.1 (2C), 129.1 (2C), 126.7 (2C), 125.7, 119.2 (2C), 95.2, 73.8, 53.9, 30.6, 21.3 (C=N_2 was not observed). **FT-IR** (cm^{-1}, neat, ATR), ṽ = 2102, 1712, 1596, 1492, 1451, 1377, 1329, 1297, 1152, 1127, 756, 730, 692, 574. **HRMS (ESI)** calcd for $C_{21}H_{17}Cl_3N_4O_3Na$ $[M+Na]^+$: 501.0258, found 501.0261.

2,2,2-trichloroethyl 2-diazo-3-(1,4-dimethyl-5-oxo-3-phenyl-4,5-dihydro-1H-pyrazol-4-yl)propanoate **(3v)**

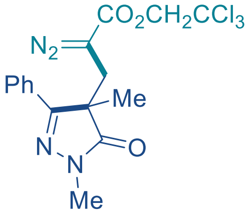

Prepared according to the *General Procedure* from the corresponding *N'*-benzylidene-*N*-methylmethacrylohydrazide **1p** (121.4 mg, 0.60 mmol, 3.0 equiv), **2g** (113.9 mg, 0.20 mmol, 1.0 equiv), Na_2CO_3 (20.0 mg, 0.2 mmol, 1.0 equiv) and 4DPAIPN (1.6 mg, 0.002 mmol, 0.01 equiv). After purification by flash column chromatography (silica gel, hexane/AcOEt 5:1), the title compound **3v** was obtained as a yellow oil (37.6 mg, 0.09 mmol, 45 %), followed by preparative TLC (silica gel, cyclohexane/AcOEt/DCM 8:1:1). **R_f** = 0.35 (silica gel, cyclohexane/AcOEt, 4:1 (v/v)). **^{1}H NMR** (600 MHz, $CDCl_3$), δ (ppm) = 7.89 – 7.74 (m, 2H), 7.46 – 7.38 (m, 3H), 5.19 – 4.44 (m, 2H), 3.42 (s, 3H), 3.07 (d, J = 15.5 Hz, 1H), 2.92 (d, J = 15.5 Hz, 1H), 1.60 (s, 3H). **^{13}C{^{1}H} NMR** (126 MHz, $CDCl_3$), δ (ppm) = 177.1, 164.8, 160.1, 130.6, 130.5, 129.0 (2C), 126.4 (2C), 95.3, 73.8, 52.1, 31.7, 30.0, 21.0 (C=N_2 was not observed). **FT-IR** (cm^{-1}, neat, ATR), ṽ = 2925, 2099, 1703, 1376, 1332, 1294, 1244, 1136, 1104, 1057, 1041, 771, 731, 717, 693, 573. **HRMS (ESI)** calcd for $C_{16}H_{15}Cl_3N_4O_3Na$ $[M+Na]^+$: 439.0102, found 439.0105.

***2,2,2-trichloroethyl 3-(3-(4-bromophenyl)-4-methyl-5-oxo-1-phenyl-4,5-dihydro-1H-pyrazol-4-yl)-2-diazopropanoate* (3w)**

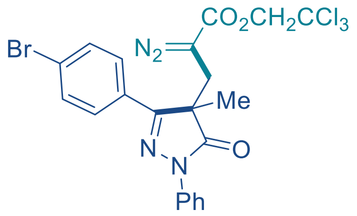

Prepared according to the *General Procedure* from the corresponding *N'*-(4-bromobenzylidene)-*N*-phenylmethacrylohydrazide **1q** (206.0 mg, 0.60 mmol, 3.0 equiv), **2g** (113.9 mg, 0.20 mmol, 1.0 equiv), Na_2CO_3 (20.0 mg, 0.2 mmol, 1.0 equiv) and 4DPAIPN (1.6 mg, 0.002 mmol, 0.01 equiv). After purification by flash column chromatography (silica gel, hexane/AcOEt 5:1), the title compound **3w** was obtained as a yellow oil (60.4 mg, 0.11 mmol, 54%). $\mathbf{R_f}$ = 0.35 (silica gel, hexane/AcOEt, 4:1 (v/v)). **^{1}H NMR** (300 MHz, $CDCl_3$), δ (ppm) = 8.00 (dd, *J* = 8.6, 1.2 Hz, 2H), 7.84 (d, *J* = 8.7 Hz, 2H), 7.69 – 7.58 (m, 2H), 7.47 (dd, *J* = 8.7, 7.3 Hz, 2H), 7.31 – 7.26 (m, 1H), 4.71 (br s, 2H), 3.19 (d, *J* = 15.5 Hz, 1H), 3.04 (d, *J* = 15.5 Hz, 1H), 1.71 (s, 3H). **^{13}C{^{1}H} NMR** (126 MHz, $CDCl_3$), δ (ppm) = 175.6, 164.7, 159.5, 137.8, 132.3 (2C), 129.3, 129.2 (2C), 128.2 (2C), 125.9, 125.3, 119.3 (2C), 95.2, 73.8, 53.9, 30.7, 21.3 ($C=N_2$ was not observed). **FT-IR** (cm^{-1}, neat, ATR), ṽ = 2107, 1670, 1601, 1489, 1387, 1329, 1179, 1167, 1069, 1008, 909, 820, 728, 694, 590, 515. **HRMS (ESI)** calcd for $C_{21}H_{16}BrCl_3N_4O_3Na$ $[M+Na]^+$: 580.9340, found 580.9347.

***2,2,2-trichloroethyl 3-(3-(4-methoxyphenyl)-4-methyl-5-oxo-1-phenyl-4,5-dihydro-1H-pyrazol-4-yl)-2-diazopropanoate* (3x)**

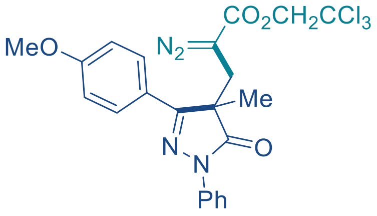

Prepared according to the *General Procedure* from the corresponding *N'*-(4-methoxybenzylidene)-*N*-phenylmethacrylohydrazide **1r** (176.6 mg, 0.60 mmol, 3.0 equiv), **2g** (113.9 mg, 0.20 mmol, 1.0 equiv), Na_2CO_3 (20.0 mg, 0.2 mmol, 1.0 equiv) and 4DPAIPN (1.6 mg, 0.002 mmol, 0.01 equiv). After purification by flash column chromatography (silica gel, hexane/AcOEt 5:1), the title compound **3x** was obtained as a yellow oil (67.7 mg, 0.13 mmol, 66 %). $\mathbf{R_f}$ = 0.20 (silica gel, hexane/AcOEt, 4:1 (v/v)). **^{1}H NMR** (400 MHz,

$CDCl_3$), δ (ppm) = 8.00 (d, *J* = 7.4 Hz, 2H), 7.90 (d, *J* = 8.9 Hz, 2H), 7.44 (t, *J* = 8.4 Hz, 2H), 7.23 (t, *J* = 7.4 Hz, 1H), 6.98 (d, *J* = 8.9 Hz, 2H), 4.69 (br s, 2H), 3.87 (s, 3H), 3.18 (d, *J* = 15.5 Hz, 1H), 3.01 (d, *J* = 15.5 Hz, 1H), 1.69 (s, 3H). **$^{13}C\{^1H\}$ NMR** (126 MHz, $CDCl_3$), δ (ppm) = 175.6, 161.6 (2C), 160.1, 138.0, 129.1 (2C), 128.4 (2C), 125.6, 123.1, 119.2 (2C), 114.5 (2C), 95.2, 73.8, 55.6, 53.9, 30.6, 21.4 ($C=N_2$ was not observed). **FT-IR** (cm^{-1}, neat, ATR), ṽ = 2103, 1704, 1607, 1498, 1376, 1328, 1310, 1298, 1253, 1174, 1151, 1128, 1108, 1031, 907, 833, 755, 726, 690, 580, 508. **HRMS (ESI)** calcd for $C_{22}H_{19}Cl_3N_4O_4Na$ $[M+Na]^+$: 531.0364, found 531.0366.

***2,2,2-trichloroethyl 3-(3-(4-((2-(4-isobutylphenyl)propanoyl)oxy)phenyl)-4-methyl-5-oxo-1-phenyl-4,5-dihydro-1H-pyrazol-4-yl)-2-diazopropanoate* (3y)**

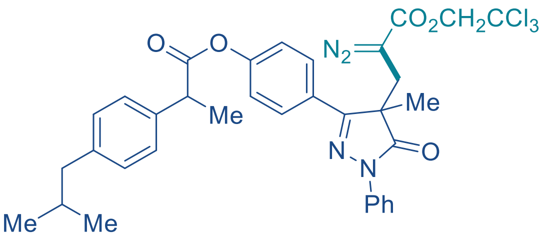

Prepared according to the *General Procedure* from the corresponding 4-((2-methacryloyl-2-phenylhydrazineylidene)methyl)phenyl 2-(4-isobutylphenyl)propanoate **1s** (281.2 mg, 0.60 mmol, 3.0 equiv), **2g** (113.9 mg, 0.20 mmol, 1.0 equiv), Na_2CO_3 (20.0 mg, 0.2 mmol, 1.0 equiv) and 4DPAIPN (1.6 mg, 0.002 mmol, 0.01 equiv). After purification by flash column chromatography (silica gel, hexane/AcOEt 5:1), the title compound **3y** was obtained as a yellow oil (73.8 mg, 0.11 mmol, 54 %). **R_f** = 0.25 (silica gel, hexane/AcOEt, 4:1 (v/v)). **^1H NMR** (500 MHz, $CDCl_3$), δ (ppm) = 7.97 (d, *J* = 7.8 Hz, 2H), 7.93 (d, *J* = 8.7 Hz, 2H), 7.44 (dd, *J* = 8.9, 7.8 Hz, 2H), 7.31 (d, *J* = 8.1 Hz, 2H), 7.27 – 7.21 (m, 1H), 7.16 (d, *J* = 8.0 Hz, 2H), 7.12 (d, *J* = 8.5 Hz, 2H), 4.69 (br s, 2H), 3.96 (q, *J* = 7.1 Hz, 1H), 3.16 (d, *J* = 15.5 Hz, 1H), 3.00 (d, *J* = 15.5 Hz, 1H), 2.49 (d, *J* = 7.2 Hz, 2H), 1.97 – 1.86 (m, 1H), 1.68 (s, 3H), 1.62 (d, *J* = 7.2 Hz, 3H), 0.92 (d, *J* = 6.6 Hz, 6H). **$^{13}C\{^1H\}$ NMR** (126 MHz, $CDCl_3$), δ (ppm) = 175.6, 173.0, 159.6, 152.7, 141.1, 137.8, 137.1, 129.7 (2C), 129.1 (2C), 127.9 (2C), 127.3 (2C), 125.8, 122.3, 122.2 (2C), 119.7, 119.2 (2C), 95.2, 73.8, 67.2, 53.8, 45.4, 45.2, 30.3, 22.5 (2C), 21.2, 18.6 ($C=N_2$ was not observed). **FT-IR** (cm^{-1}, neat, ATR), ṽ = 2954, 2106, 1756, 1672, 1331, 1201, 1180, 1161, 1132, 1087, 1067, 908, 894, 728, 694, 529. **HRMS (ESI)** calcd for $C_{34}H_{33}Cl_3N_4O_5Na$ $[M+Na]^+$: 705.1409, found 705.1409.

***4-(4-(2-diazo-3-oxo-3-(2,2,2-trichloroethoxy)propyl)-4-methyl-5-oxo-1-phenyl-4,5-dihydro-1H-3-yl)phenyl 5-(2,5-dimthylphenoxy)-2,2-dimethylpentanoate* (3z)**

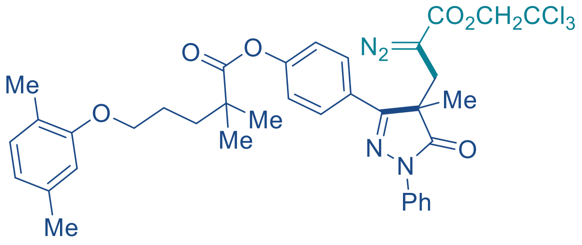

Prepared according to the *General Procedure* from the corresponding 4-((2-methacryloyl-2-phenylhydrazineylidene)methyl)phenyl 5-(2,5-dimethylphenoxy)-2,2-dimethylpentanoate **1t** (307.6 mg, 0.60 mmol, 3.0 equiv), **2g** (113.9 mg, 0.20 mmol, 1.0 equiv), Na_2CO_3 (20.0 mg, 0.2 mmol, 1.0 equiv) and 4DPAIPN (1.6 mg, 0.002 mmol, 0.01 equiv). After purification by flash column chromatography (silica gel, hexane/AcOEt 5:1), the title compound **3z** was obtained as a yellow oil (51.0 mg, 0.07 mmol, 35%). $\mathbf{R_f}$ = 0.25 (silica gel, hexane/AcOEt, 4:1 (v/v)). **^{1}H NMR** (500 MHz, $CDCl_3$), δ (ppm) = 7.99 (d, *J* = 7.4 Hz, 2H), 7.96 (d, *J* = 8.6 Hz, 2H), 7.45 (dd, *J* = 8.6, 7.3 Hz, 2H), 7.26 – 7.23 (m, 1H), 7.15 (d, *J* = 8.3 Hz, 2H), 7.01 (d, *J* = 7.5 Hz, 1H), 6.68 (d, *J* = 7.4 Hz, 1H), 6.64 (s, 1H), 4.68 (br s, 2H), 4.00 (t, *J* = 5.5 Hz, 2H), 3.18 (d, *J* = 15.5 Hz, 1H), 3.02 (d, *J* = 15.5 Hz, 1H), 2.31 (s, 3H), 2.19 (s, 3H), 1.91 – 1.88 (m, 4H), 1.69 (s, 3H), 1.39 (s, 6H). **^{13}C{^{1}H} NMR** (126 MHz, $CDCl_3$), δ (ppm) = 176.1, 175.6, 171.7, 157.0, 152.9, 137.9, 136.7, 130.5, 129.3, 129.1 (2C), 128.0 (2C), 127.9, 125.8, 123.7, 122.3 (2C), 121.0, 119.2 (2C), 112.1, 95.2, 73.8, 67.9, 53.9, 42.7, 37.3, 25.4, 25.3, 25.2, 21.6, 21.2, 18.9, 16.0 (C=N_2 was not observed). **FT-IR** (cm^{-1}, neat, ATR), ṽ = 2929, 2106, 1711, 1598, 1505, 1377, 1261, 1205, 1155, 1128, 1102, 1062, 1045, 1003, 908, 802, 754, 729, 692. **HRMS (ESI)** calcd for $C_{36}H_{37}Cl_3N_4O_6Na$ $[M+Na]^+$: 749.1671, found 749.1673.

2,2,2-trichloroethyl 3-((5-chloro-1,3-dimethyl-2-oxoindolin-3-yl)-2-(phenylamino)propanoate **(4)**

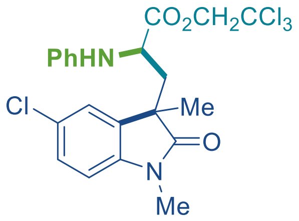

Adapted from a reported procedure from the literature.[10] To an 8 mL vial equipped with a magnetic stir bar, product **3f** (42.5 mg, 0.1 mmol, 1.0 equiv), aniline (18.6 mg, 0.2 mmol, 1.0 equiv) and $[RuCl_2(p\text{-cymene})]_2$ (1.0 mg, 1 mol %) were added. Subsequently, the vial was subjected to 3 cycles of vacuum/argon, followed by the addition of dry DCM (1.0 mL, 0.1 M) and the reaction mixture was stirred at room temperature overnight. After, it was concentrated under vacuum. After purification by flash column chromatography (hexane/DCM 1:1 to DCM 100 %), the title compound **4** was obtained as a colorless oil (35.2 mg, 0.07 mmol, 72 %, *dr* = 1:1). $\mathbf{R_f}$ = 0.63 (silica gel, DCM 100 %). **^{1}H NMR** (600 MHz, $CDCl_3$), δ (ppm) = 7.25 – 7.18 (m, 2H), 7.10 –

[10] Deng, Q. H.; Xu, H. W.; Yuen, A. W. H.; Xu, Z. J.; Che, C. M. *Org. Lett.* **2008**, *10*, 1529-1532.

7.01 (m, 2H), 6.71 – 6.60 (m, 2H), 6.34 – 6.21 (m, 2H), 4.77 – 4.61 (m, 2H), 4.20 (td, *J* = 8.8, 5.3 Hz, 1H), 3.56 (d, *J* = 9.3 Hz, 1H), 2.92 (s, 3H), 2.64 (dd, *J* = 14.4, 5.5 Hz, 1H), 2.37 (dd, *J* = 14.4, 8.9 Hz, 1H), 1.41 (s, 3H). **^{13}C{^{1}H} NMR** (151 MHz, $CDCl_3$), δ (ppm) = 178.9, 171.7, 145.6, 141.9, 136.0, 129.2, 128.1, 128.1, 122.5, 118.8, 113.3, 109.6, 94.6, 74.4, 54.3, 47.0, 39.7, 26.3, 25.5. **FT-IR** (cm^{-1}, neat, ATR), ṽ = 1754, 1704, 1602, 1490, 1429, 1345, 1271, 1130, 1026, 907, 809, 725, 691, 648, 575, 546, 475. **HRMS (ESI)** calcd for $C_{21}H_{21}Cl_4N_2O_3$ [M+H]$^+$: 491.0274, found 491.0281.

2,2,2-trichloroethyl 2-((5-chloro-1,3-dimethyl-2-oxoindolin-3-yl)methyl)-3,3,3-trifluoropropanoate **(5)**

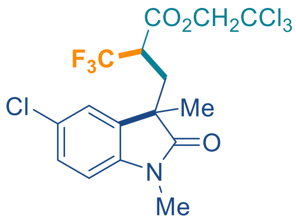

Adapted from a reported procedure from the literature.[11] To an 8 mL vial equipped with a magnetic stir bar, CuI (56.0 mg, 0.3 mmol, 3.0 equiv) and CsF (50 mg, 0.33 mmol, 3.3 equiv) were added. Subsequently, the vial was subjected to 3 cycles of vacuum/argon, followed by the addition of $TMSCF_3$ (48 µL, 0.33 mmol, 3.3 equiv) and NMP (1.0 mL). The resulting mixture was left stirring at room temperature for 30 minutes. Then, product **3f** (42.5 mg, 0.1 mmol, 1.0 equiv), in NMP (0.5 mL) was added, followed by H_2O (79 µL, 4.4 mmol, 4.4 equiv) and the reaction mixture was stirred at room temperature overnight. After this time, HCl (1 mL, 1M) was added and the mixture was extracted with DCM (3x5 mL). The combined organic layers were washed with H_2O, brine, dried over Na_2SO_4 and concentrated under vacuum. After purification by flash column chromatography (hexane/AcOEt 4:1), the title compound **5** was obtained as a colorless oil (30.2 mg, 0.07 mmol, 65 %, *dr* = 1.3:1). **R_f** = 0.25 (silica gel, hexane/AcOEt 4:1). **^{1}H NMR** (600 MHz, $CDCl_3$), δ (ppm) = 7.35 – 7.26 (m, 1H), 7.22 – 7.12 (m, 1H), 6.84 – 6.73 (m, 1H), 4.79 – 4.67 (m, 1H), 4.51 – 4.29 (m, 1H), 4.16 – 4.04 (m, 1H), 3.22 (s, 1.3*3H), 3.17 (s, 1.6*3H), 3.05 – 2.84 (m, 1H), 2.76 – 2.41 (m, 1H), 1.40 (s, 1.7*3H), 1.36 (1.3*3H). **^{13}C{^{1}H} NMR** (151 MHz, $CDCl_3$), δ (ppm) = 178.4, 178.3, 169.9, 169.4, 142.2, 141.9, 133.9, 132.6, 128.9, 128.7, 124.6, 124.5, 123.6, 123.2, 109.6, 109.6, 94.6, 94.4, 74.9, 74.8, 49.8, 49.1, 44.1, 43.7, 26.7, 26.5, 24.3, 23.6, 11.9, 11.3. **^{19}F NMR** (282 MHz, $CDCl_3$), δ (ppm) = -68.4 (1.4*3F), -68.6 (1.6*3F). **FT-IR** (cm^{-1}, neat, ATR), ṽ = 1713, 1610, 1489, 1346, 1243, 1150, 907, 809, 727, 648, 571, 547, 465. **HRMS (ESI)** calcd for $C_{16}H_{15}Cl_4F_3NO_3$ [M+H]$^+$: 467.9725, found 467.9728.

[11] Hu, M.; Ni, C.; Hu, J. *J. Am. Chem. Soc.* **2012**, *134*, 15257-15260.

2,2,2-trichloroethyl E-3-(5-chloro-1,3-dimethyl-2-oxoindolin-3-yl)acrylate and 2,2,2-trichloroethyl Z-3-(5-chloro-1,3-dimethyl-2-oxoindolin-3-yl)acrylate **(6)**

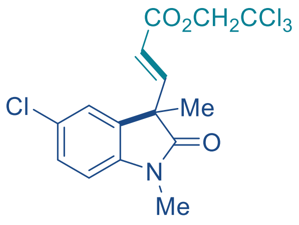

Adapted from a protocol from the literature.[12] To an 8 mL vial equipped with a magnetic stir bar, product **3f** (42.5 mg, 0.1 mmol, 1.0 equiv) and Cs_2CO_3 (32.6 mg, 0.1 mmol, 1.0 equiv) were added. Subsequently, the vial was subjected to 3 cycles of vacuum/argon, followed by the addition of dry Acetone (1.0 mL, 0.1 M) and the reaction mixture was irradiated under 40 W, 456 nm Kessil lamp at room temperature overnight. Then, the reaction mixture was washed with water, extracted with DCM (3x5 mL) and dried over anhydrous Na_2SO_4. After, it was concentrated under vacuum followed by purification by flash column chromatography (hexane/AcOEt 4:1), obtaining the title compound **6** as a colorless oil (22.9 mg, 0.06 mmol, 59%, *E*/*Z* = 1.5:1). $\mathbf{R_f}$ = 0.15 (silica gel, hexane/AcOEt 4:1).

***E* isomer:**

^{1}H NMR (600 MHz, $CDCl_3$), δ (ppm) = 7.32 (dd, *J* = 8.3, 1.9 Hz, 1H), 7.21 (d, *J* = 1.9 Hz, 1H), 7.09 (d, *J* = 15.8 Hz, 1H), 6.83 (d, *J* = 8.3 Hz, 1H), 6.02 (d, *J* = 15.9 Hz, 1H), 4.81 (d, *J* = 12.0 Hz, 1H), 4.76 (d, *J* = 12.0 Hz, 1H), 3.23 (s, 3H), 1.59 (s, 3H). **^{13}C{^{1}H} NMR** (126 MHz, $CDCl_3$), δ (ppm) = 176.3, 164.3, 148.8, 141.6, 132.8, 129.0, 128.6, 124.5, 120.6, 109.8, 94.9, 74.3, 51.0, 28.8, 22.6. **FT-IR** (cm^{-1}, neat, ATR), ṽ = 1716, 1642, 1608, 1490, 1452, 1410, 1373, 1342, 1267, 1213, 1152, 1118, 1087, 1050, 1030, 964, 807, 768, 726, 647, 572, 548, 446. **HRMS (ESI)** calcd for $C_{15}H_{14}Cl_4NO_3$ $[M+H]^+$: 397.9694, found 397.9697.

***Z* isomer:**

^{1}H NMR (600 MHz, $CDCl_3$), δ (ppm) = 7.23 (dd, *J* = 8.3, 2.1 Hz, 1H), 7.04 (d, *J* = 2.1 Hz, 1H), 6.78 (d, *J* = 8.3 Hz, 1H), 6.47 (d, *J* = 11.7, 1H), 6.10 (d, *J* = 11.7 Hz, 1H), 4.57 (d, *J* = 12.0 Hz, 1H), 4.46 (d, *J* = 12.0 Hz, 1H), 3.24 (s, 3H), 1.61 (s, 3H). **^{13}C{^{1}H} NMR** (151 MHz, $CDCl_3$), δ (ppm) = 178.1, 162.5, 149.1, 142.9, 135.2, 128.0, 127.7, 122.8, 121.8, 109.0, 94.7, 73.9, 49.5, 28.1, 26.6. **FT-IR** (cm^{-1}, neat, ATR), ṽ = 1717, 1648, 1609,

[12] Xia, D.; Li, T.; Ke, X.-Y.; Wang, J.; Luan, X.; Ni, S.-F.; Zhang, Y.; Zhang, W.-D. *J. Org. Chem.* **2024**, *89*, 6180-6192.

1490, 1373, 1344, 1295, 1151, 1027, 986, 813, 720, 572, 547. **HRMS (ESI)** calcd for $C_{15}H_{14}Cl_4NO_3$ $[M+H]^+$: 397.9694, found 397.9696.

The same product was obtained when an intramolecular C–H activation was tried using $Rh_2(esp)_2$ (1 mol %) as catalyst through a Bamford-Stevens reaction (14.9 mg, 0.05 mmol, 48 %, *Z*/*E* > 20:1).

***2,2,2-trichloroethyl 2-methoxy-3-(5-methoxy-1,3-dimethyl-2-oxoindolin-3-yl)propanoate* (7)**

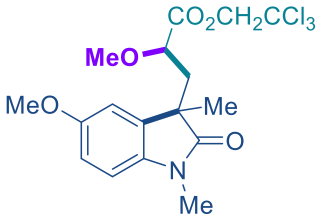

Adapted from a protocol from the literature.[13] To an 8 mL vial equipped with a magnetic stir bar, product **3f** (42.1 mg, 0.1 mmol, 1.0 equiv) and $Cu(MeCN)_4PF_6$ (1.5 mg, 4 mol %) were added. Subsequently, the vial was subjected to 3 cycles of vacuum/argon, followed by the addition of dry DCM (0.5 mL, 0.2 M) and MeOH (21 µL, 0.5 mmol, 5.0 equiv). The resulting reaction mixture was degassed with Ar for 30 s and the it was stirred at room temperature overnight. After, it was concentrated under vacuum followed by purification by flash column chromatography (hexane/AcOEt 4:1 – 3:1), obtaining the title compound **7** as a pale yellow oil (17.8 mg, 0.04 mmol, 42%). $\mathbf{R_f}$ = 0.45 (silica gel, hexane/AcOEt 3:2). ^{1}**H NMR** (600 MHz, $CDCl_3$), δ (ppm) = 6.82 – 6.77 (m, 2H), 6.75 (d, *J* = 8.3 Hz, 1H), 4.73 (d, *J* = 11.9 Hz, 1H), 4.65 (d, *J* = 11.9 Hz, 1H), 3.80 (s, 3H), 3.73 (dd, *J* = 7.3, 4.7 Hz, 1H), 3.20 (s, 3H), 3.11 (s, 3H), 2.50 (dd, *J* = 14.5, 4.7 Hz, 1H), 2.29 (dd, *J* = 14.5, 7.3 Hz, 1H), 1.39 (s, 3H). ^{13}**C{^{1}H} NMR** (151 MHz, $CDCl_3$), δ (ppm) = 179.5, 170.6, 156.1, 136.9, 134.8, 112.0, 111.1, 108.3, 94.8, 78.0, 74.2, 58.5, 55.9, 46.9, 40.1, 26.5, 25.2. **FT-IR** (cm^{-1}, neat, ATR), ṽ = 2927, 1764, 1701, 1600, 1498, 1470, 1358, 1289, 1212, 1036, 909, 802, 726, 647, 573, 464. **HRMS (ESI)** calcd for $C_{17}H_{20}Cl_3NO_5Na$ $[M+Na]^+$: 446.0305, found 446.0307.

List of unsuccessful substrates

[13] He, Q.; Zhang, Q.; Rolka, A. B.; Suero, M. G. *J. Am. Chem. Soc.* **2024**, *146*, 12294-12299.

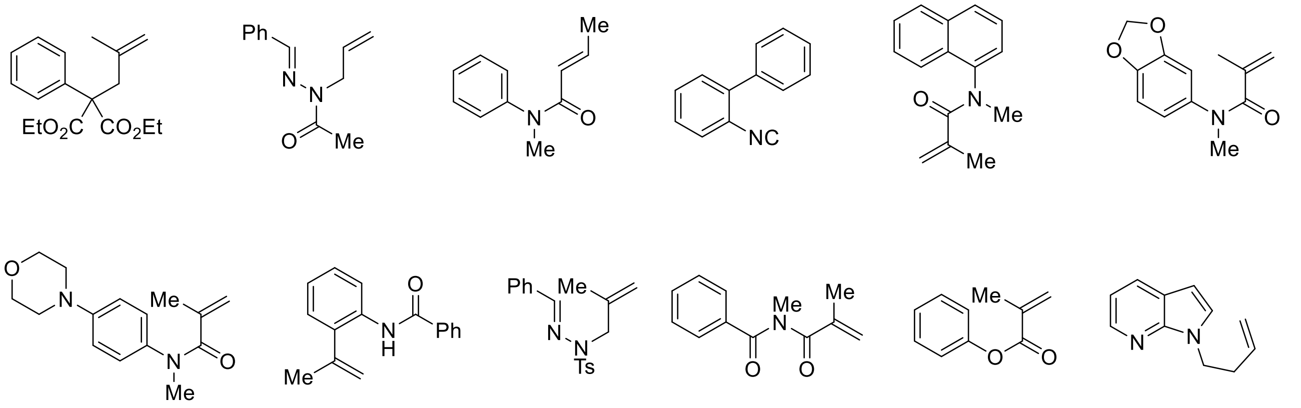

4. Large scale synthesis of 3f

To an 8 mL Chemglass vial equipped with a magnetic stir bar, *N*-(4-chlorophenyl)-*N*-methylacrylamide **1e** (629.0 mg, 3.0 mmol, 3.0 equiv), diazo reagent **2g** (569.5 mg, 1.0 mmol, 1.0 equiv) and Na_2CO_3 (106.0 mg, 1.0 mmol, 1.0 equiv) were added. Subsequently, the vial was subjected to 3 cycles of vacuum/argon, followed by the addition of dry MeCN (5.0 mL, 0.2 M). The resulting reaction mixture was first degassed with Ar for 30 s and then irradiated for 3 h with a strip of white LED as described in the "*Workflow*" section. The temperature of the reaction was maintained at approximately 25 °C with the use of a fan. Upon completion, the solvent was removed under reduced pressure. After purification by flash column chromatography (hexane/AcOEt 4:1), the title compound **3f** was obtained as a yellow oil (276.3 mg, 0.64 mmol, 64 %). $\mathbf{R_f}$ = 0.58 (silica gel, hexane/AcOEt, 3:2 (v/v)). **^{1}H NMR** (600 MHz, $CDCl_3$), δ (ppm) = 7.30 – 7.19 (m, 2H), 6.78 (d, J = 8.9 Hz, 1H), 4.81 (d, J = 12.1 Hz, 1H), 4.64 (br s, 1H), 3.22 (s, 3H), 2.95 (d, J = 15.2 Hz, 1H), 2.81 (d, J = 15.7 Hz, 1H), 1.46 (s, 3H). **^{13}C{^{1}H} NMR** (151 MHz, $CDCl_3$), δ (ppm) = 178.9, 165.0, 141.9, 133.9, 128.5, 128.3, 124.3, 109.2, 95.3, 73.6, 48.6, 31.3, 26.6, 22.6 ($C=N_2$ was not observed). ^{1}H-^{1}H COSY, ^{1}H-^{13}C HSQC and ^{1}H-^{13}C HMBC experiments were performed. **FT-IR** (cm^{-1}, neat, ATR), ṽ = 2096, 1704, 1610, 1489, 1375, 1335, 1149, 1124, 1055, 824, 717, 575, 546. **HRMS (ESI)** calcd for $C_{15}H_{13}Cl_4N_3O_3Na$ $[M+Na]^+$: 445.9603, found 445.9605.

5. Mechanistic Investigation

5.1. BHT experiment

To a 4 mL Chemglass vial equipped with a magnetic stir bar, acrylamide **1a** (52.6 mg, 0.30 mmol, 3.0 equiv), diazo reagent **2g** (57.0 mg, 0.10 mmol, 1.0 equiv), Na_2CO_3 (10.0 mg, 0.20 mmol, 1.0 equiv) and BHT (110.2 mg, 0.5 mmol, 5 equiv) were added. Subsequently, the vial was subjected to 3 cycles of vacuum/argon, followed by the addition of dry MeCN (0.5 mL, 0.2 M). The resulting reaction mixture was first degassed with Ar for 30 s and then irradiated for 3 h with a strip of white LED as described in the "*Workflow*" section. The temperature of the reaction was maintained at approximately 25 °C with the use of a fan. Upon completion, the solvent was removed under reduced pressure and the crude mixture was analysed by ^{1}H-NMR.

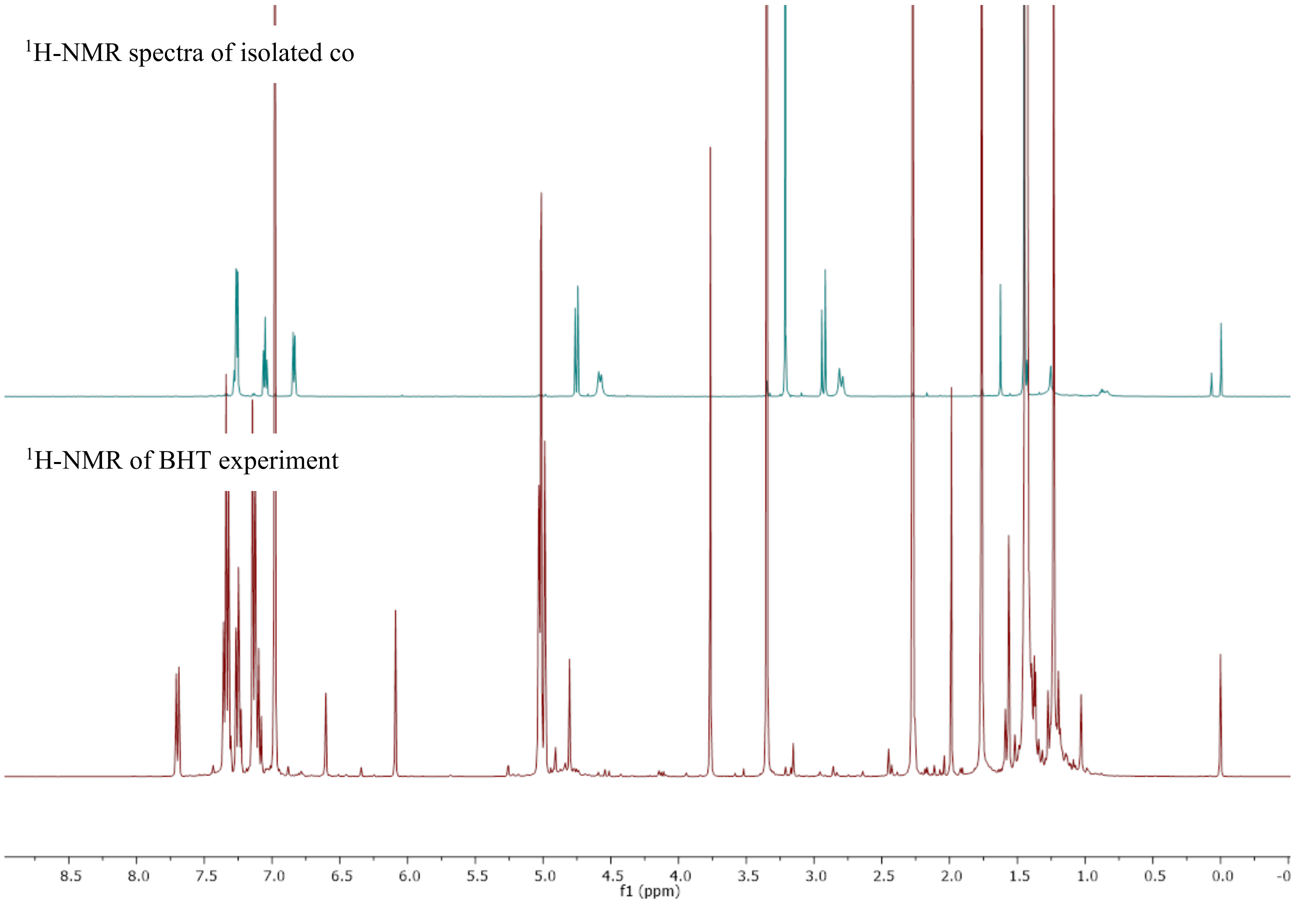

Figure S2 *Comparison between ^{1}H NMR (300 MHz, $CDCl_3$) of the BHT experiment reaction using 1,3,5-trimethoxybenzene as internal standard and the pure spectra of* ***3b***.

5.2. *Cyclic voltammetry*

Cyclic voltammetry experiments of diazo substrates **2e**, **2f** and **2g** were recorded together with acrylamide **1a** and hydrazide **1p**.

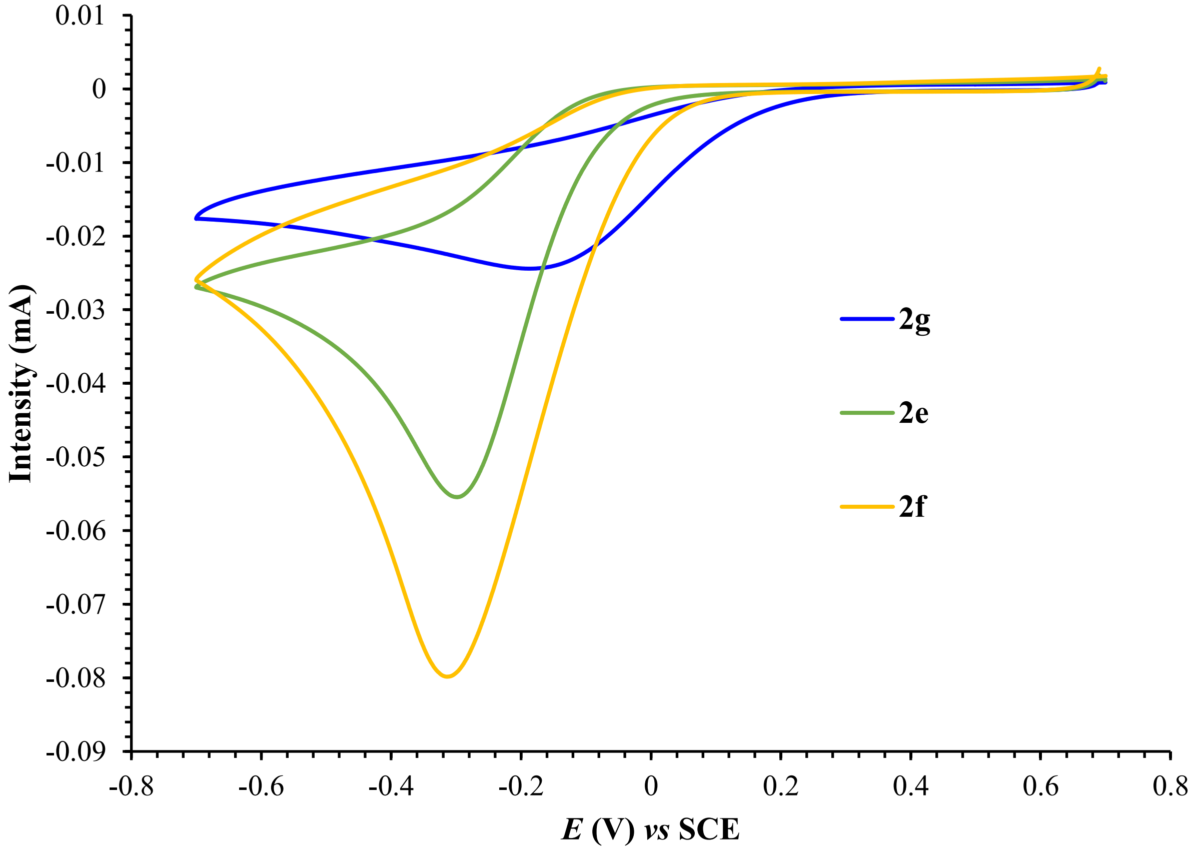

Figure S3 *Cyclic voltammetry of redox active species* ***2g*** *(blue),* ***2e*** *(green) and* ***2f*** *(yellow). Conditions:* ***2*** *2.5 mM (100 mV/s) in MeCN, TBAPF$_6$ 0.1 M, rt. Start at 0.7 V, scan direction to negative potentials. Glassy carbon disk as working electrode, platinum wire as auxiliary electrode and SCE as reference electrode. IUPAC plotting.*

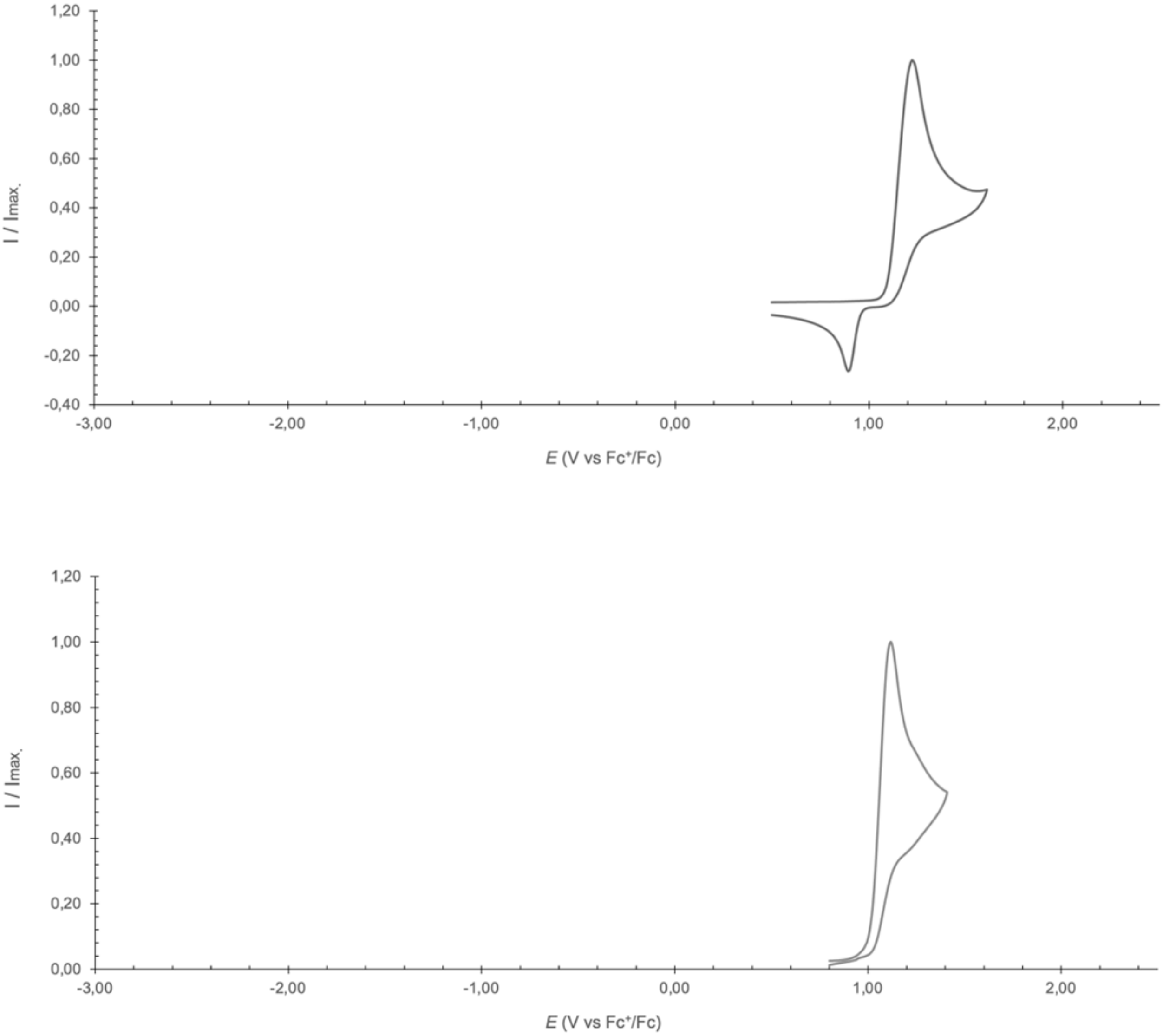

Figure S3-bis *Cyclic voltammetry of redox active species* ***1p*** *(bottom),* ***1a*** *(top). Conditions: 2.5 mM (100 mV/s) in MeCN, $TBAPF_6$ 0.1 M, rt. Start at 0.7 V, scan direction to negative potentials. Glassy carbon disk as working electrode, platinum wire as auxiliary electrode and Ag/AgCl as reference electrode. IUPAC plotting.*

Compound	$E_{c/2}$	*Compound*	$E_{a/2}$
**2e**	-0.17	**1a**	1.46
**2f**	-0.13	**1p**	1.56
**2g**	-0.12	**-**	-

Table S3 *Collected redox data of the presented work for relevant compounds.*

All values are given in V *vs* SCE.

5.3. UV-Vis studies

Different solutions of hypervalent iodine compound **2g**, radical acceptors **1a** or **1p** and Na_2CO_3 in ACN were prepared containing the concentrations of each compound depicted below. UV-Vis absorption spectra were measured in a 1 cm quartz cuvette. Absorption of individual reaction components and mixtures thereof were recorded. A bathochromic shift was observed for a mixture of acrylamide **1a**, diazo **2g** and Na_2CO_3 in ACN. This indicates the interaction between the three reagents. On the other hand, the interaction between hydrazide **1p**, diazo **2g** and Na_2CO_3 in ACN is much weaker, showing a very mild bathochromic shift when mixed.

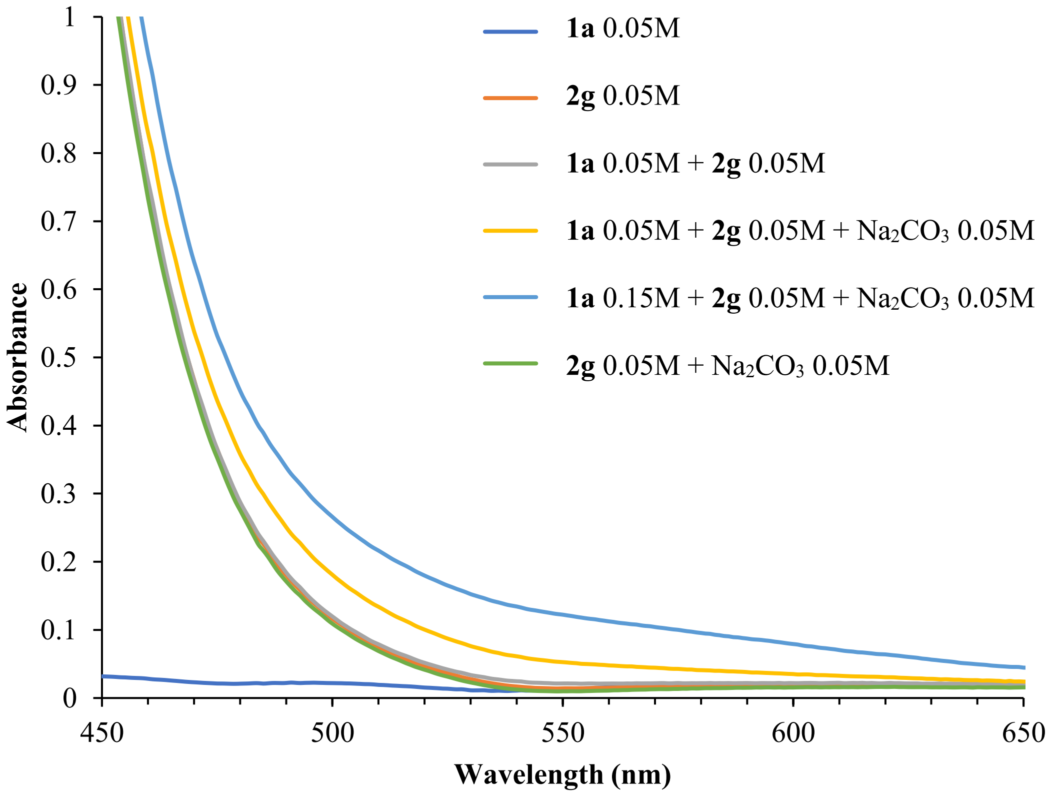

***Figure S4** UV-Vis absorption of individual components and a combination thereof. All spectra were measured in ACN. The concentration of samples reflects the used reaction conditions.*

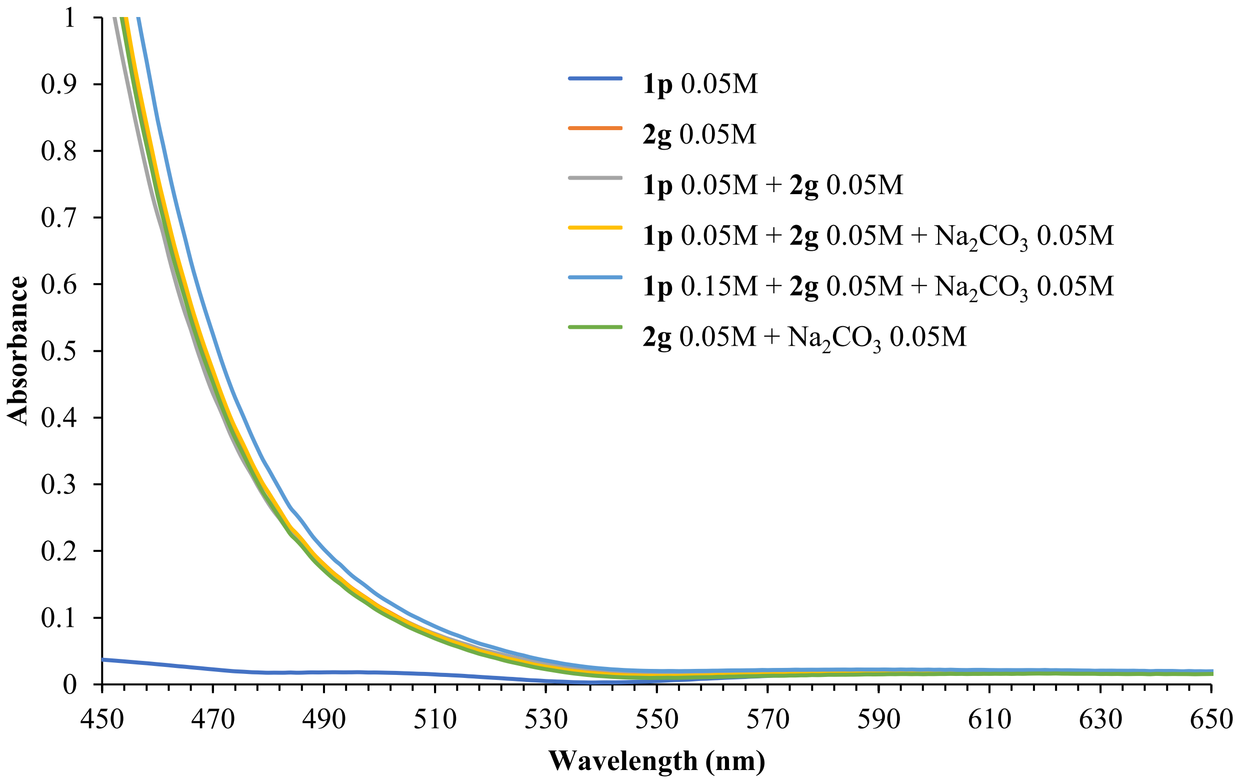

Figure S5 *UV-Vis absorption of individual components and a combination thereof. All spectra were measured in ACN. The concentration of samples reflects the reaction conditions used.*

5.5. *Stern-Volmer quenching studies*

Fluorescence measurements were obtained using septa-capped UV-Quartz cuvettes (10 mm pathlength) obtained from Hellma Analytics. Excitation was performed at 400 nm; fluorescence spectra were obtained from 300-700 nm. The stock solutions were prepared as follows:

1. Photocatalyst 4DPAIPN solution (0.0002 M): To a 25 mL volumetric flask was added 3.90 mg (0.005 mmol) of 4DPAIPN and was dissolved in ACN obtaining a 2.00×10^{-4} M solution.
2. Diazo compound **2g** solution (0.004 M): To a 10 mL volumetric flask was added 22.8 mg (0.04 mmol) of **2g** and was dissolved in ACN obtaining a 4.00×10^{-3} M solution.
3. Hydrazide **1p** solution (0.004 M): To a 10 mL volumetric flask was added 8.1 mg (0.04 mmol) of **1p** and was dissolved in ACN obtaining a 4.00×10^{-3} M solution.

Following preparation, the solutions were allocated to the cuvettes and fluorescence quenching was determined with individual quencher mixtures. 1 mL of the Photocatalyst solution was added together with 1 mL of ACN for the initial measurement. For the experiments with quencher, 1 mL of Photocatalyst solution was added together with increasing amounts of quencher (0, 10, 50, 100 and 200 µL as indicated) and adjusting

concentration with the corresponding volume of ACN to reach 2 mL. Degassing of each individual solution for 20 seconds was performed prior to recording the data.

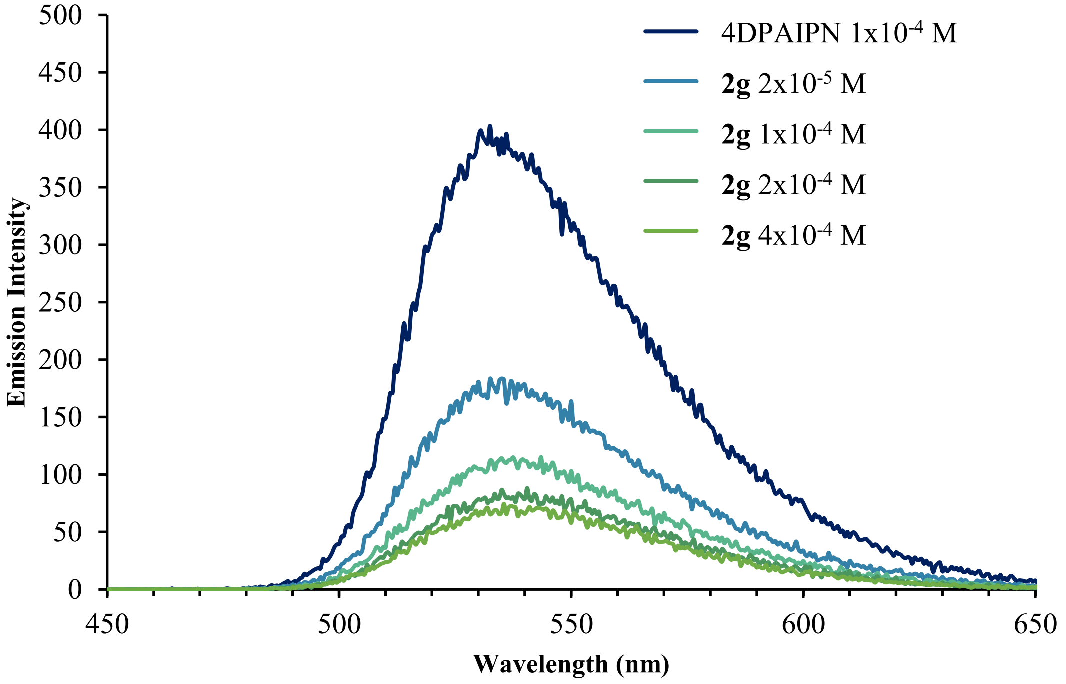

Figure S6 *Emission plot luminescence quenching of 4DPAIPN (2.0 x 10^{-4} M in degassed ACN) by addition of known amounts (10, 50, 100 and 200 μL) of diazo compound* ***2g*** *solution 2, $\lambda_{exc.}$ = 400 nm.*

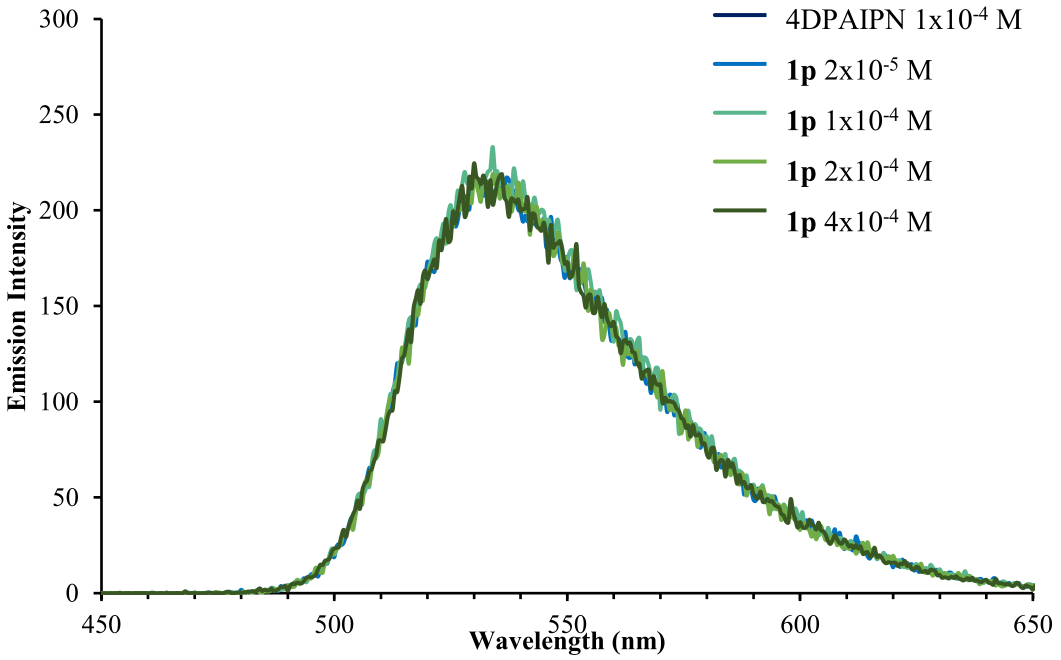

Figure S7 *Emission plot luminescence quenching of 4DPAIPN (2.0 x 10^{-4} M in degassed ACN) by addition of known amounts (10, 50, 100 and 200 μL) of hydrazide* ***1p*** *solution 3,* $\lambda_{exc.}$ *= 400 nm.*

Linear regression of I_0/I against concentration was carried out to yield the Stern-Volmer quenching rate constant (K_{SV}). The following Stern-Volmer plots for luminescence quenching of 4DPAIPN (2.0 × 10^{-4} M in degassed ACN) by quenchers were obtained. The excited catalyst is only quenched by the diazo compound **2g** with a Stern-Volmer quenching rate constant of 11,937 M^{-1}.

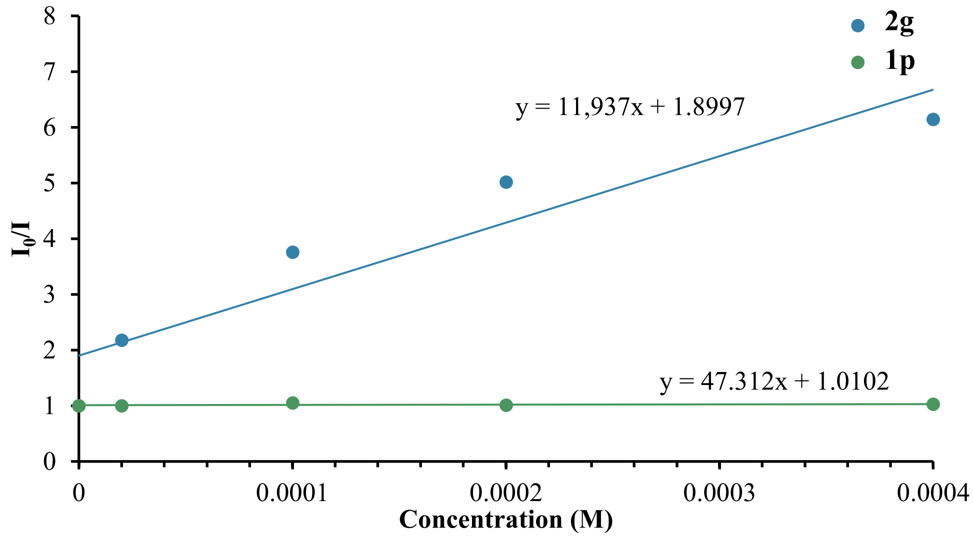

Figure S8 *Stern-Volmer plots for luminescence quenching of 4DPAIPN (2.0 x 10^{45} M in degassed ACN) by addition of diazo compound* ***2g*** *(blue) and hydrazide* ***1p*** *(green),* $\lambda_{exc.}$ *= 400 nm,* $\lambda_{em.}$ *= 530 nm,* K_{sv} *= Stern-Volmer constant.*

5.6. Three-component EDA

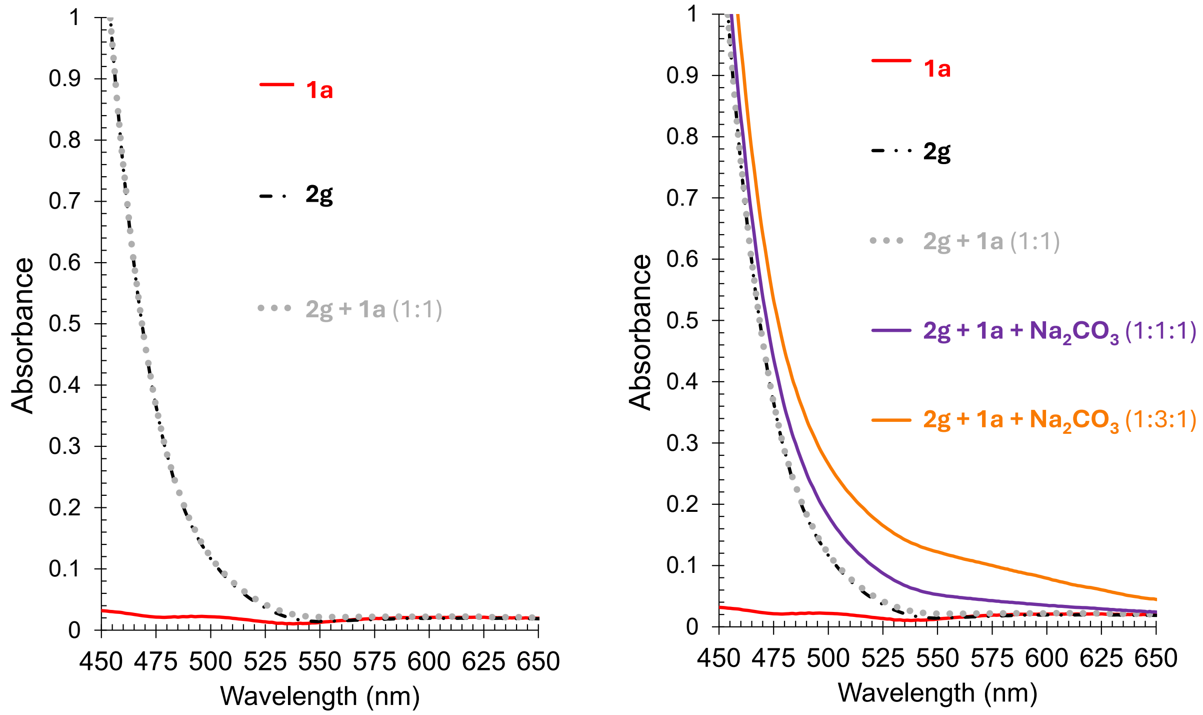

Figure S9 *UV-Vis absorption of individual components and a combination thereof. All spectra were measured in ACN.*

6. NMR Spectra

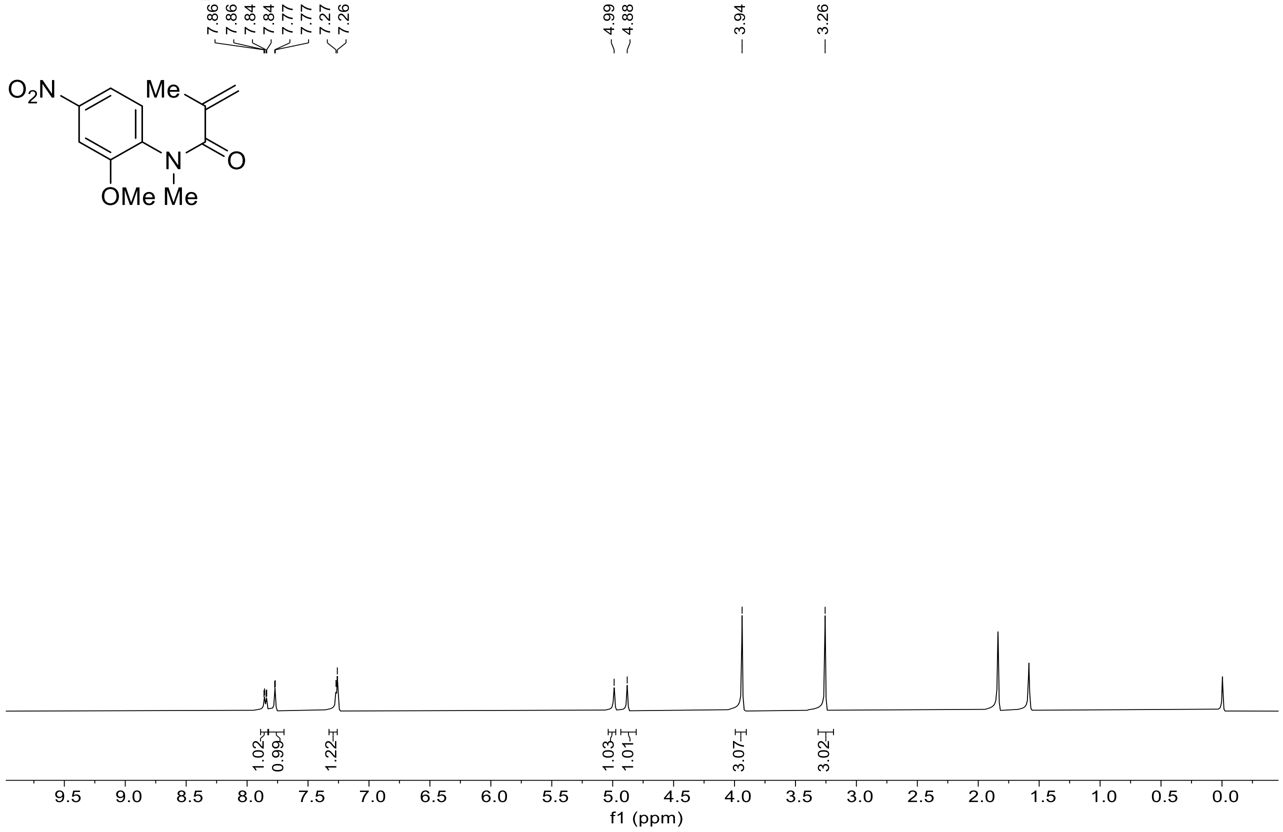

^{1}H NMR (500 MHz, $CDCl_3$) of compound **1i**

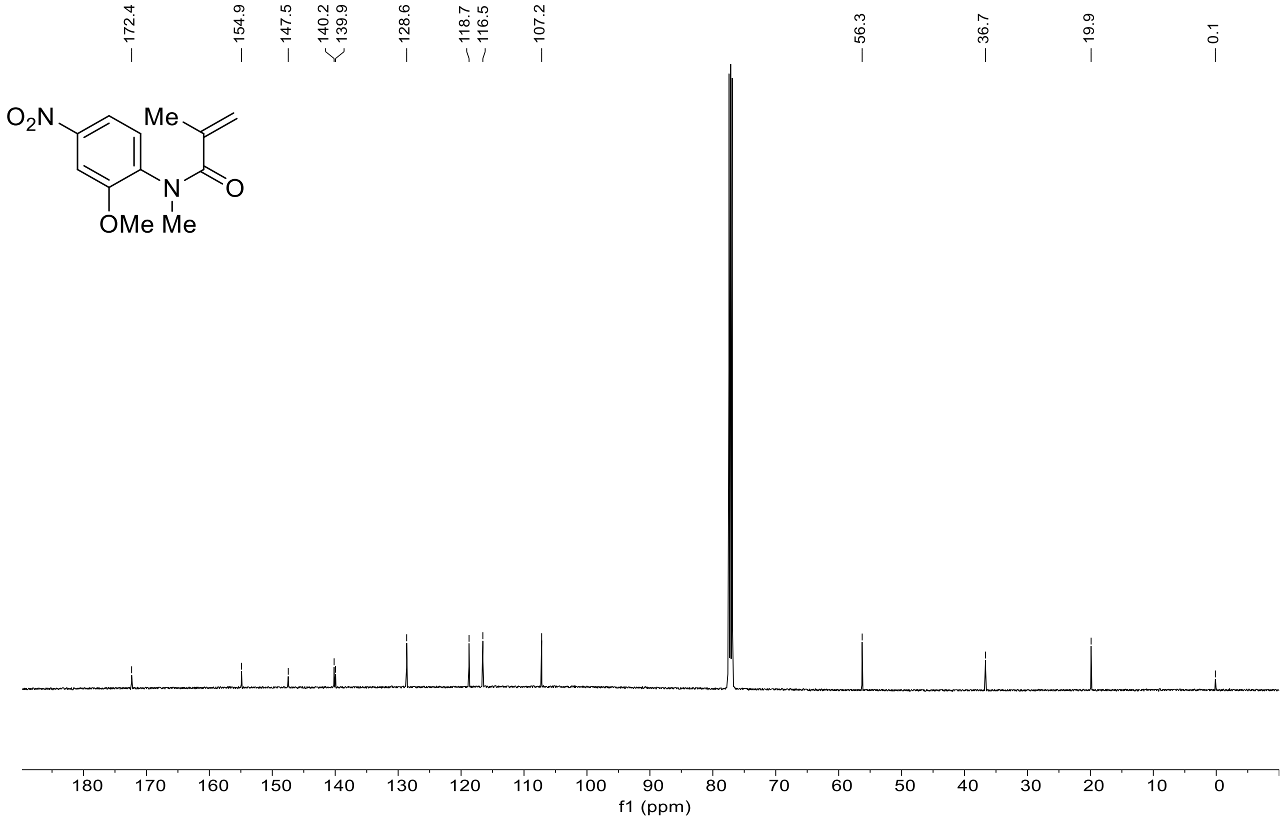

^{13}C NMR (126 MHz, $CDCl_3$) of compound **1i**

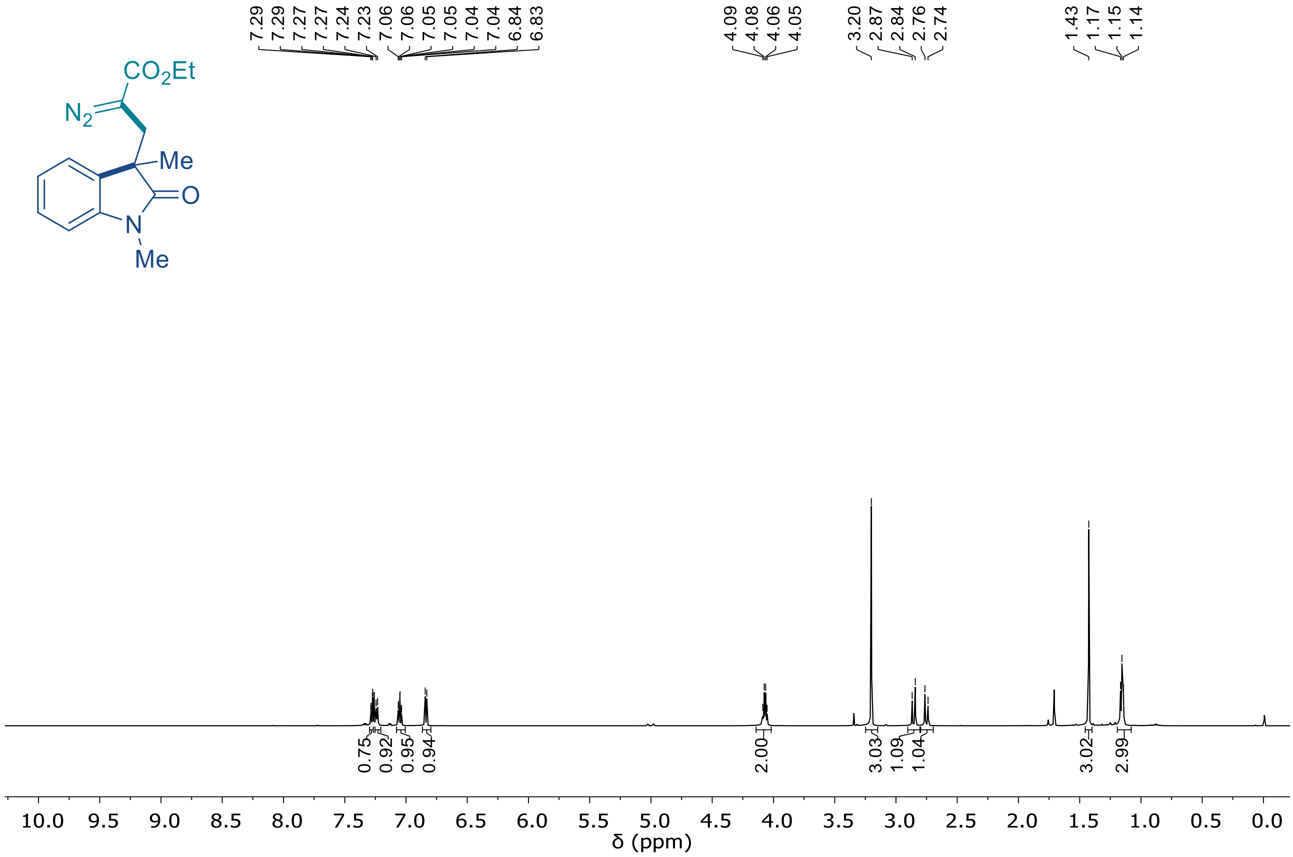

^{1}H NMR (600 MHz, $CDCl_3$) of compound **3a**

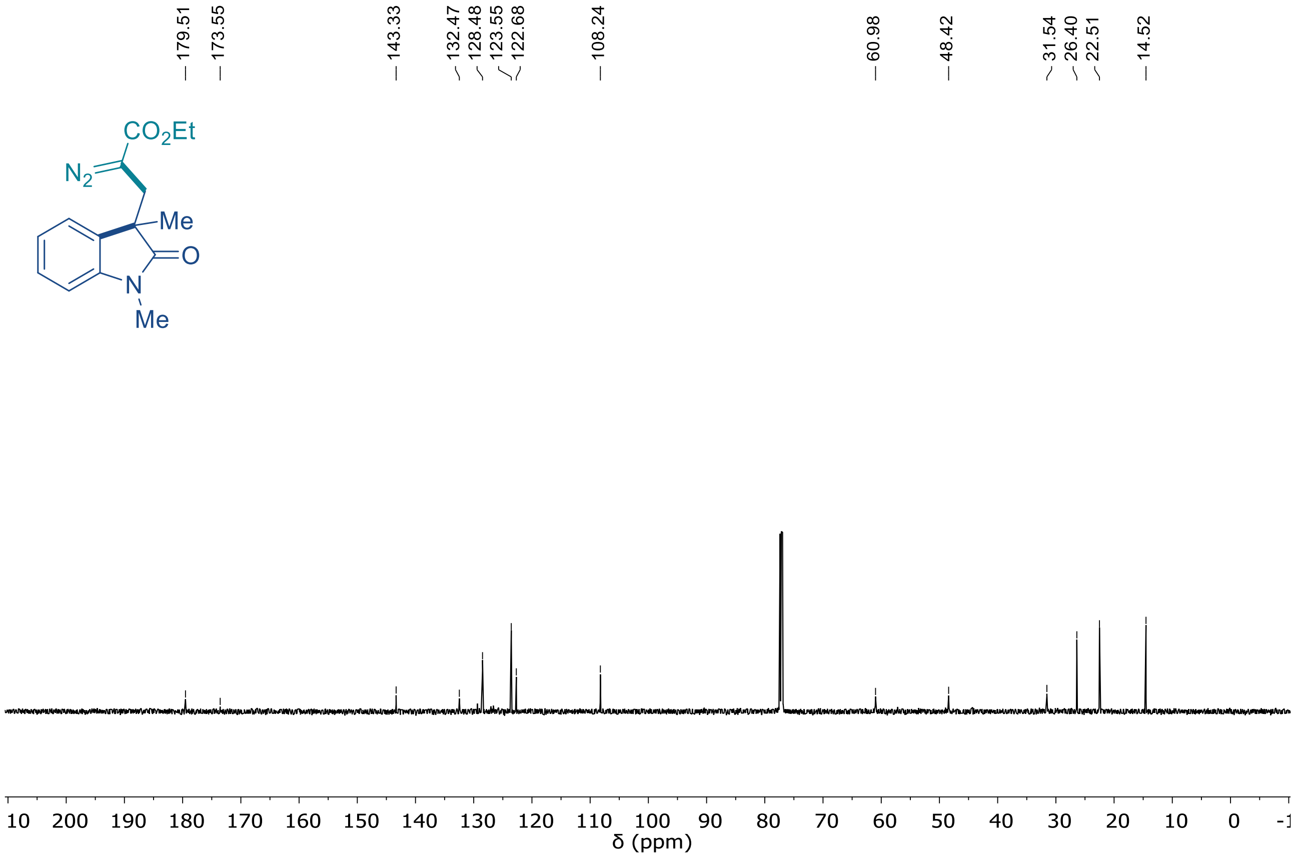

^{13}C NMR (151 MHz, $CDCl_3$) of compound **3a**

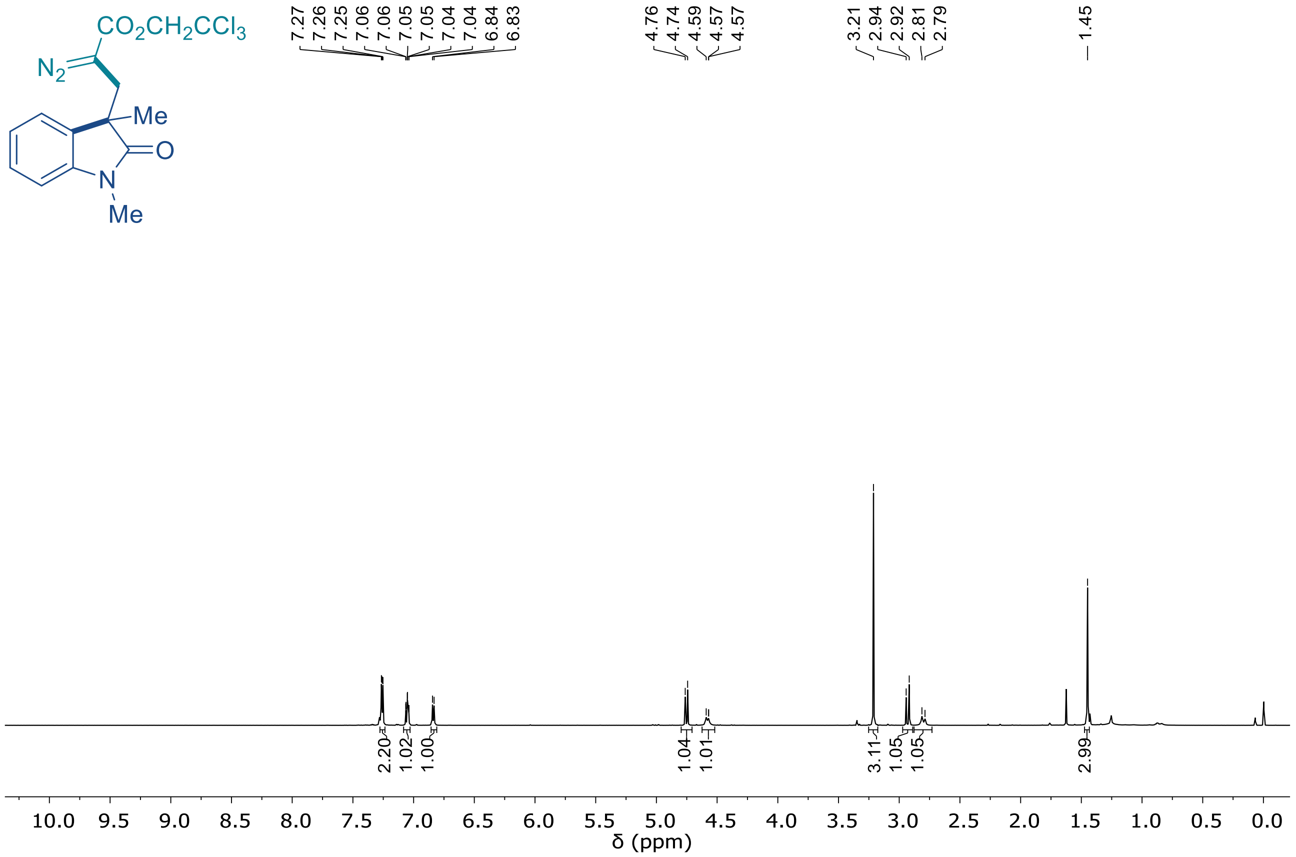

^{1}H NMR (600 MHz, CDCl3) of compound **3b**

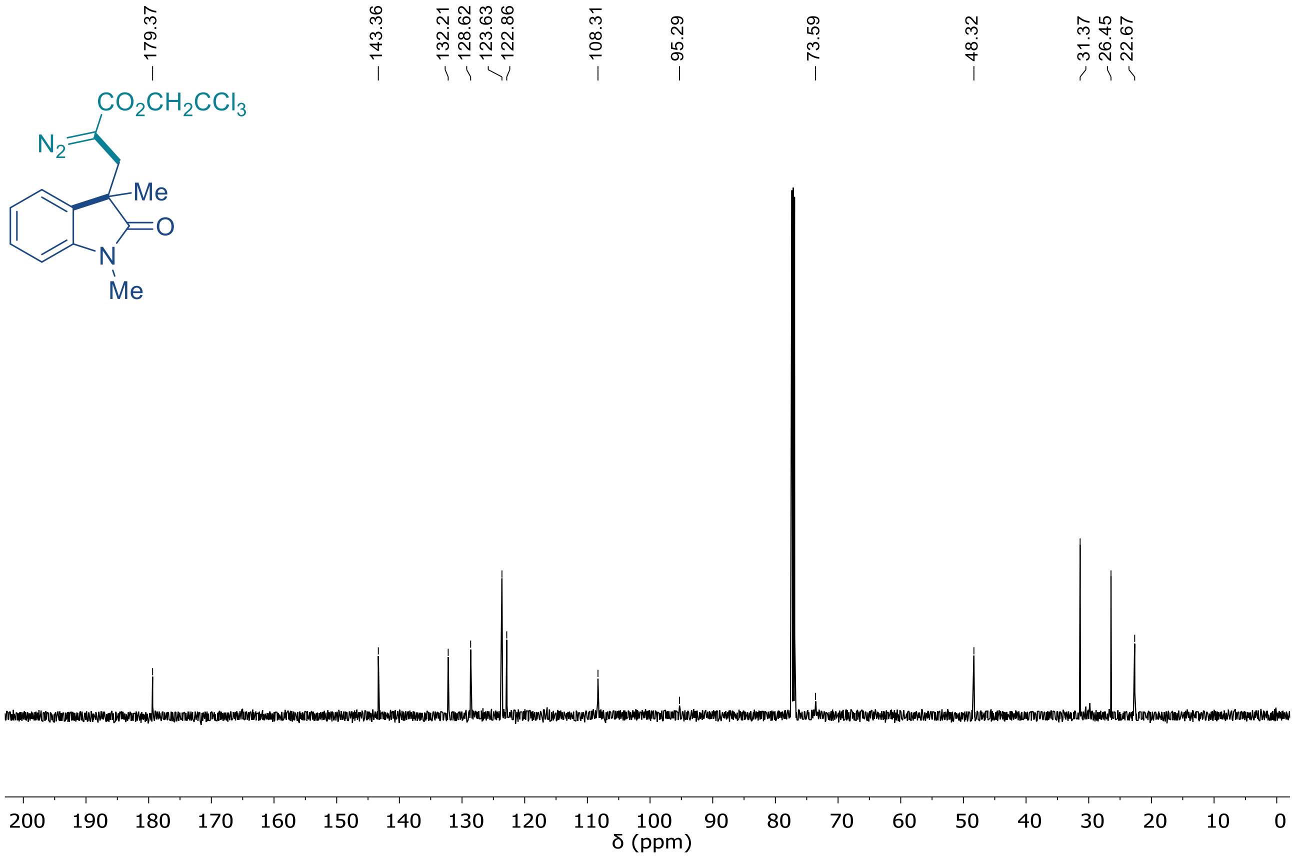

^{13}C NMR (151 MHz, CDCl3) of compound **3b**

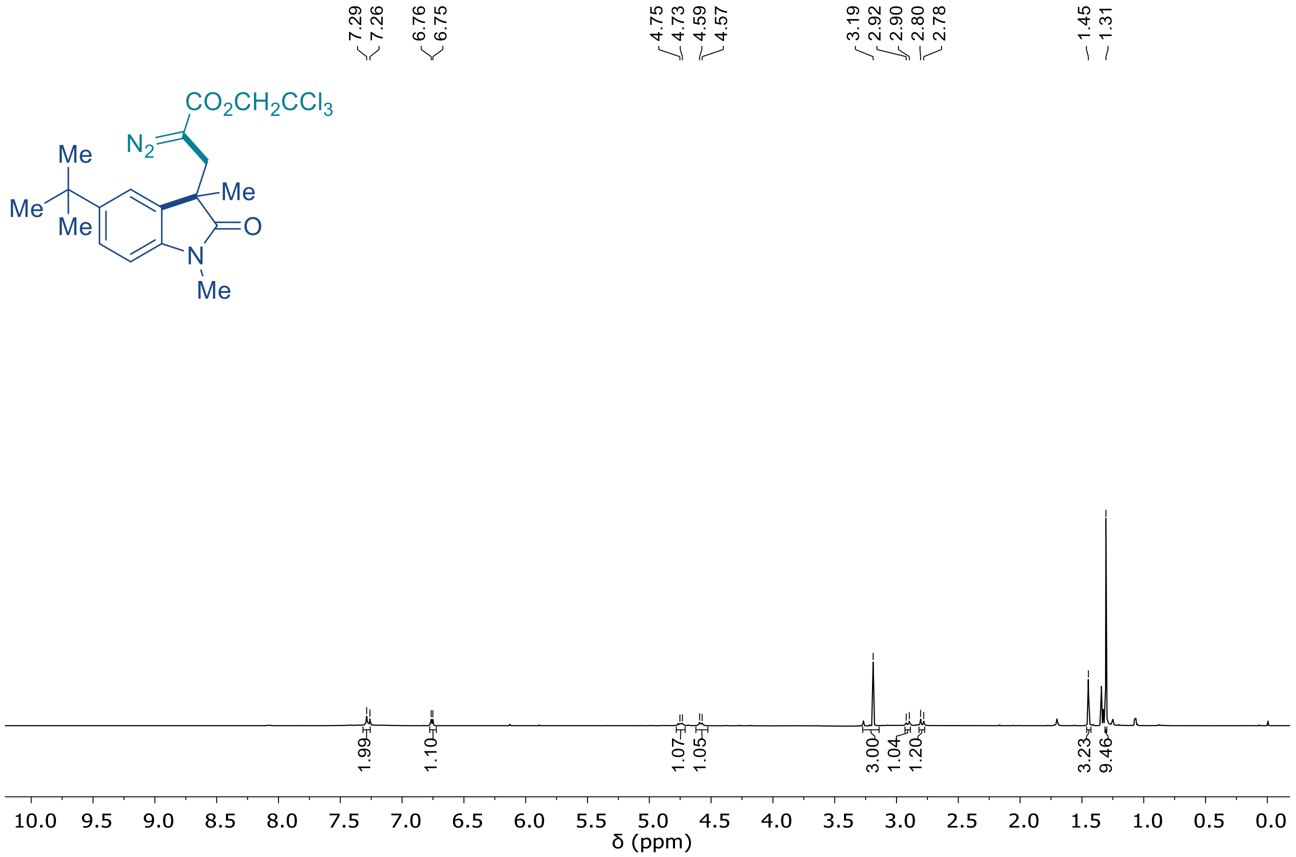

^{1}H NMR (600 MHz, $CDCl_3$) of compound **3c**

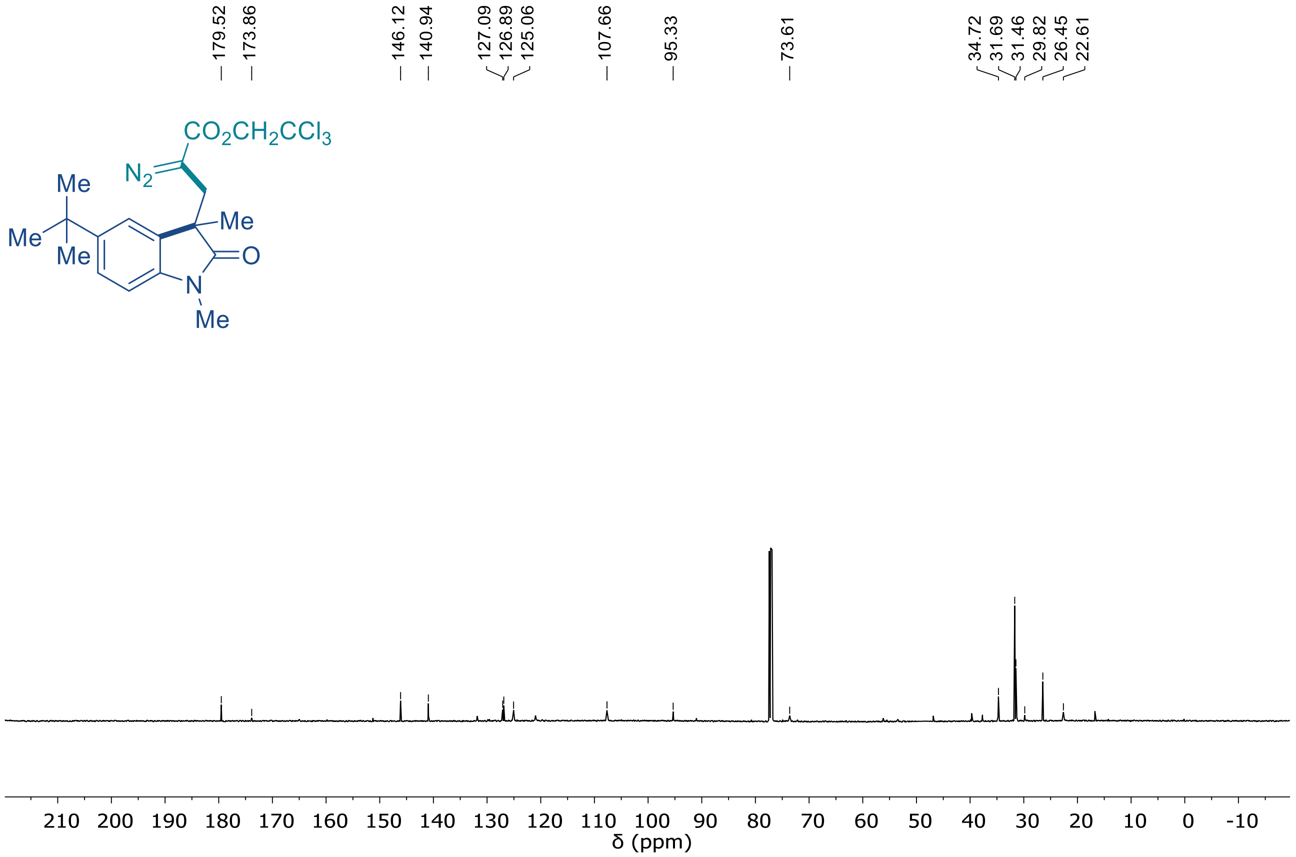

^{13}C NMR (126 MHz, $CDCl_3$) of compound **3c**

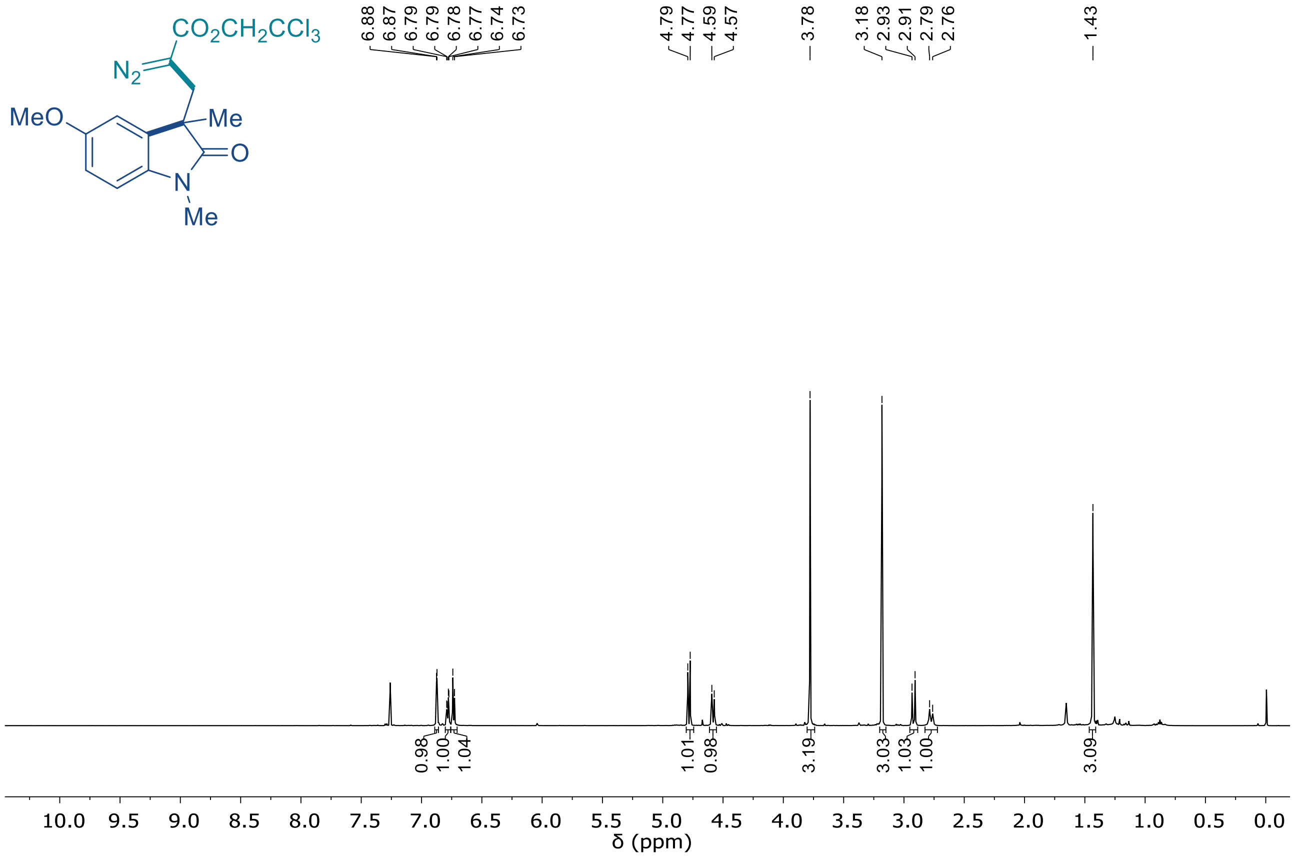

^{1}H NMR (600 MHz, $CDCl_3$) of compound **3d**

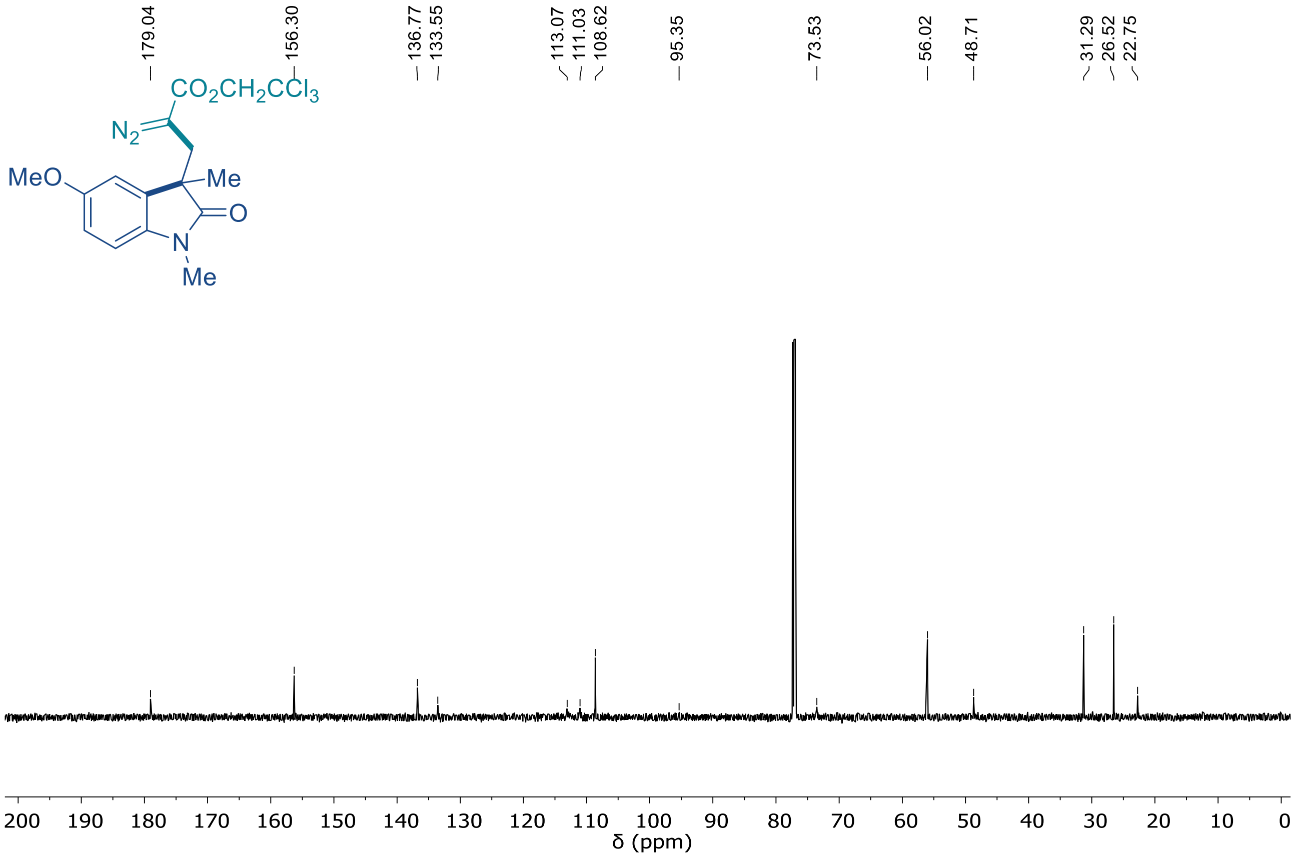

^{13}C NMR (126 MHz, $CDCl_3$) of compound **3d**

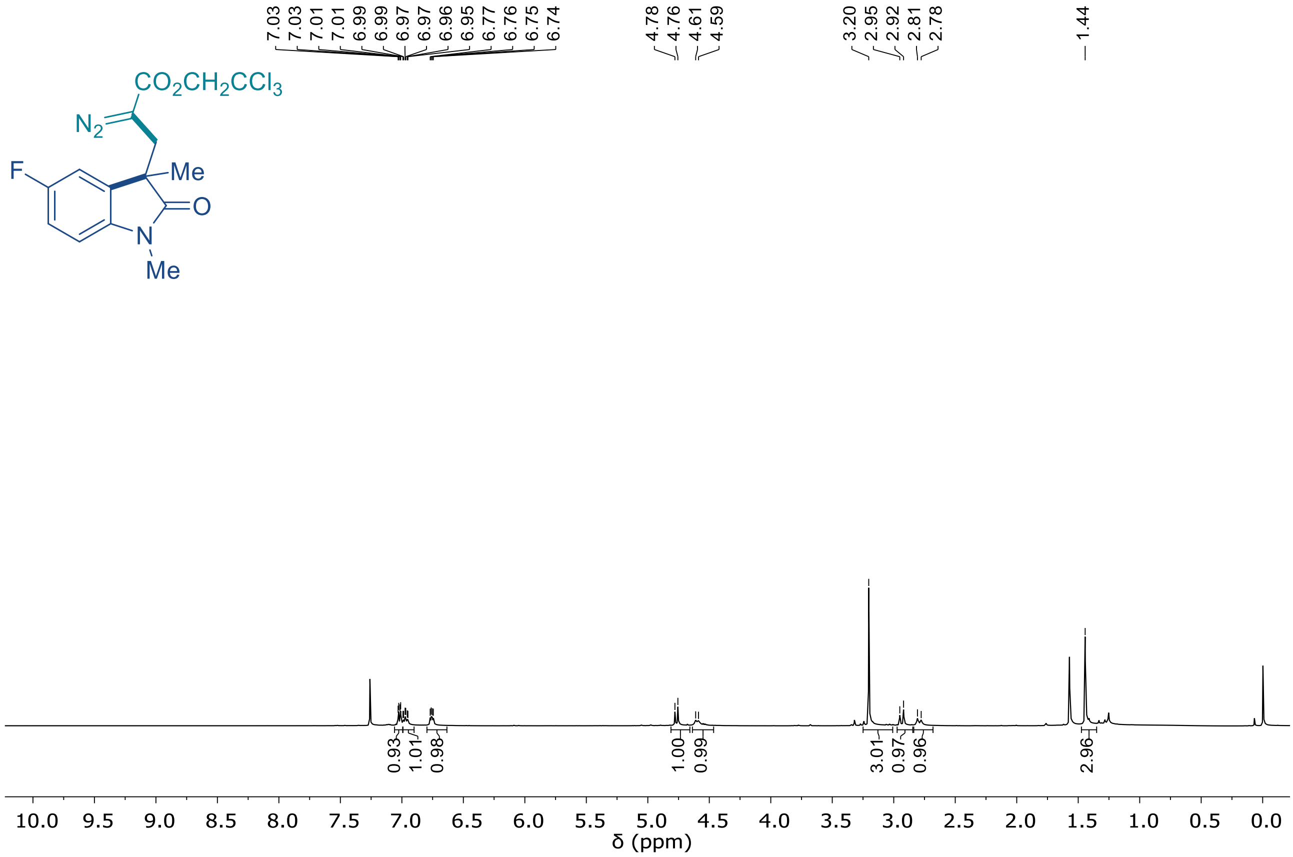

^{1}H NMR (500 MHz, $CDCl_3$) of compound **3e**

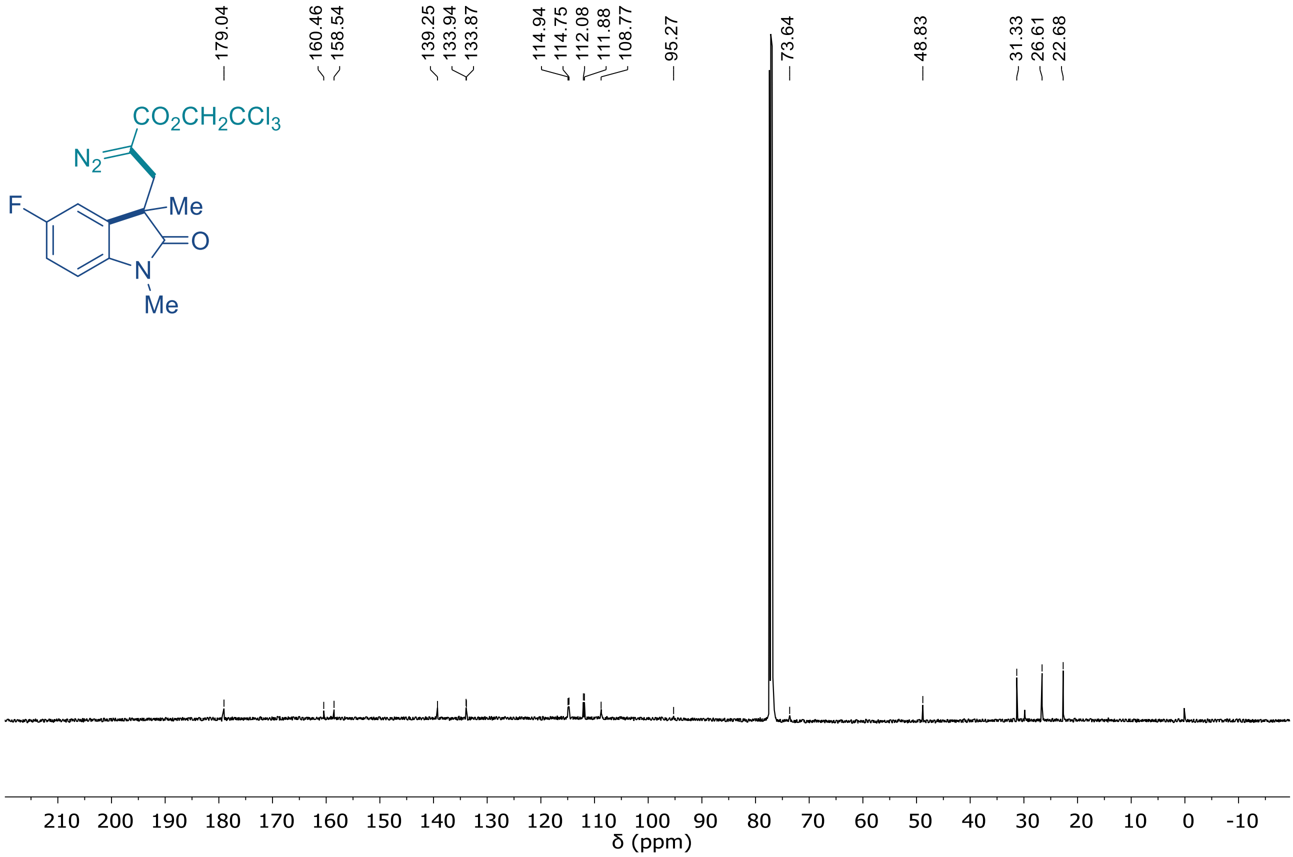

^{13}C NMR (126 MHz, $CDCl_3$) of compound **3e**

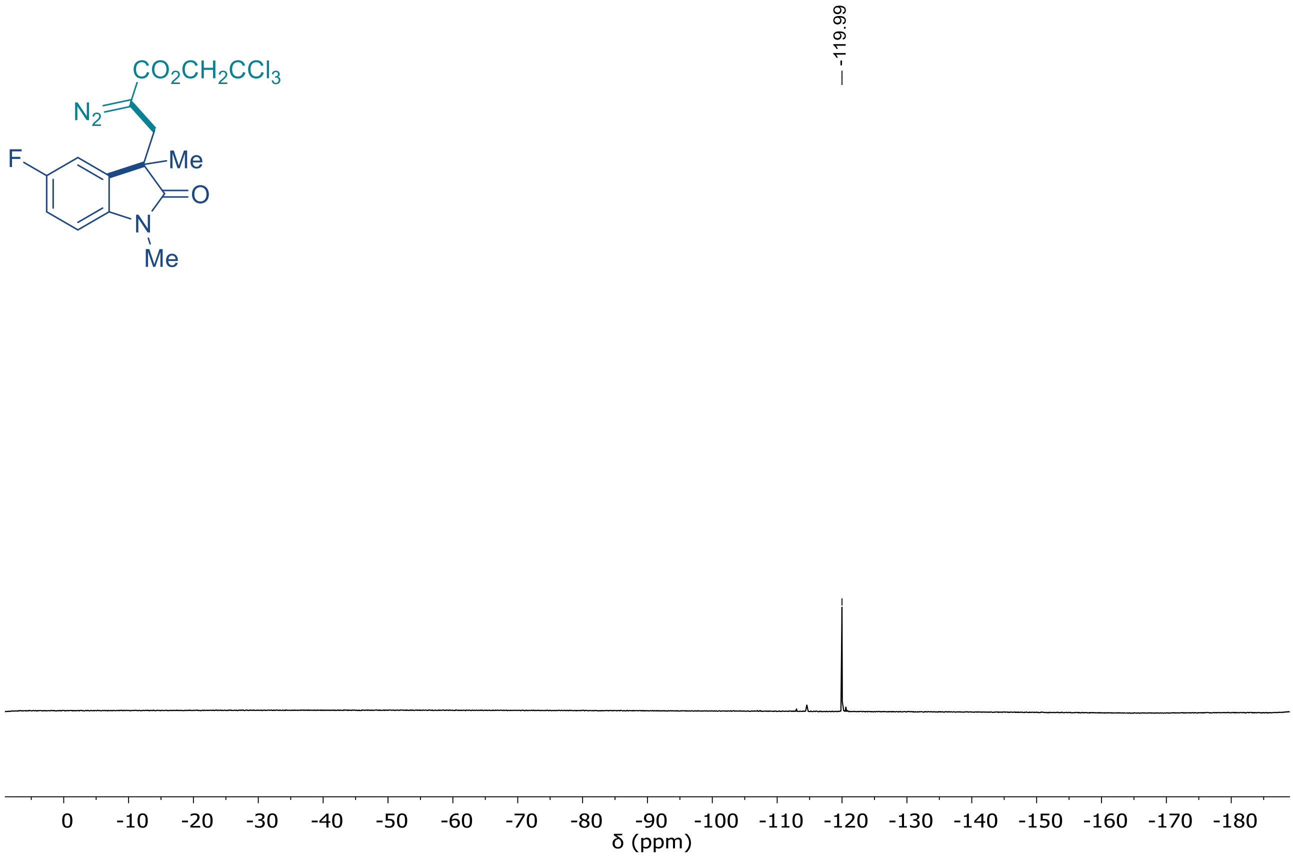

^{19}F NMR (377 MHz, CDCl$_3$) of compound **3e**

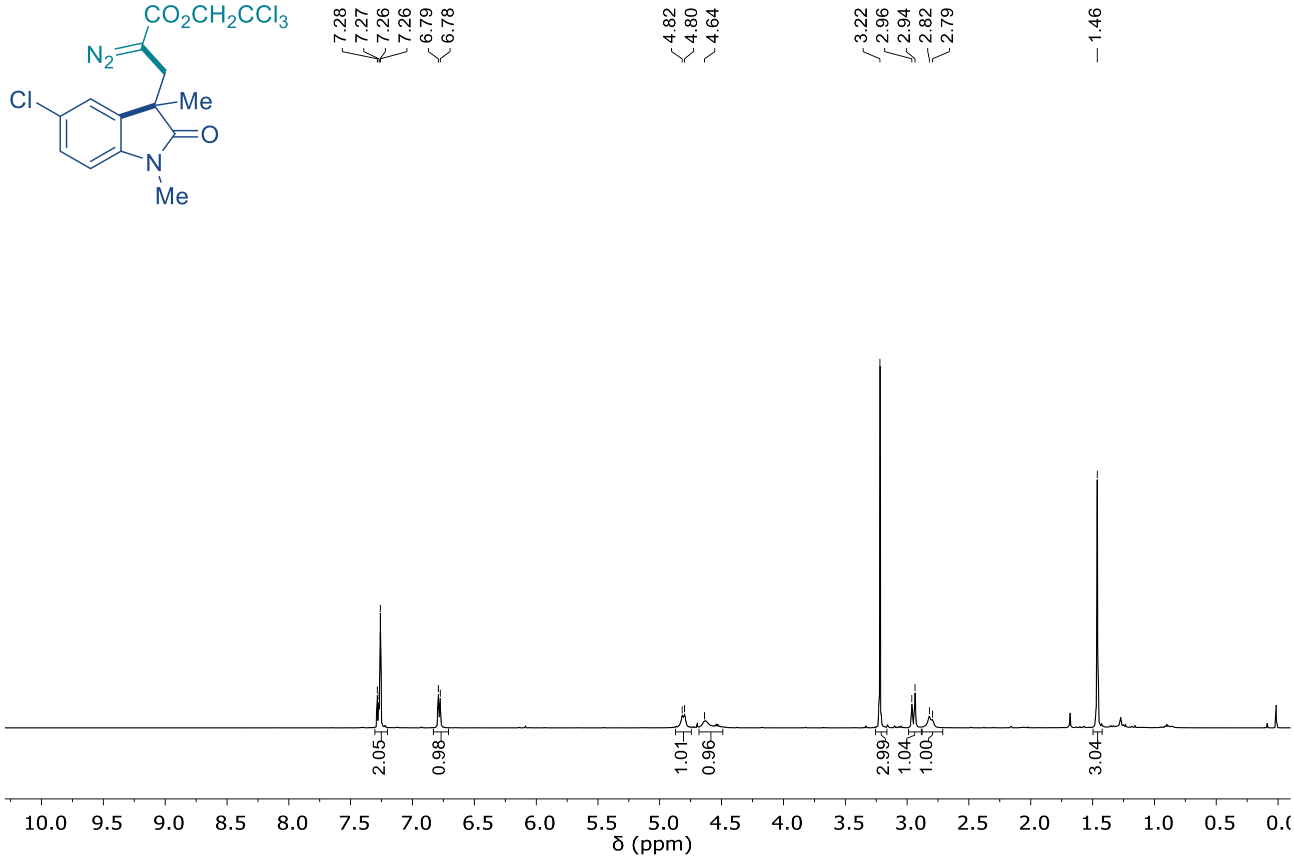

^{1}H NMR (600 MHz, CDCl$_3$) of compound **3f**

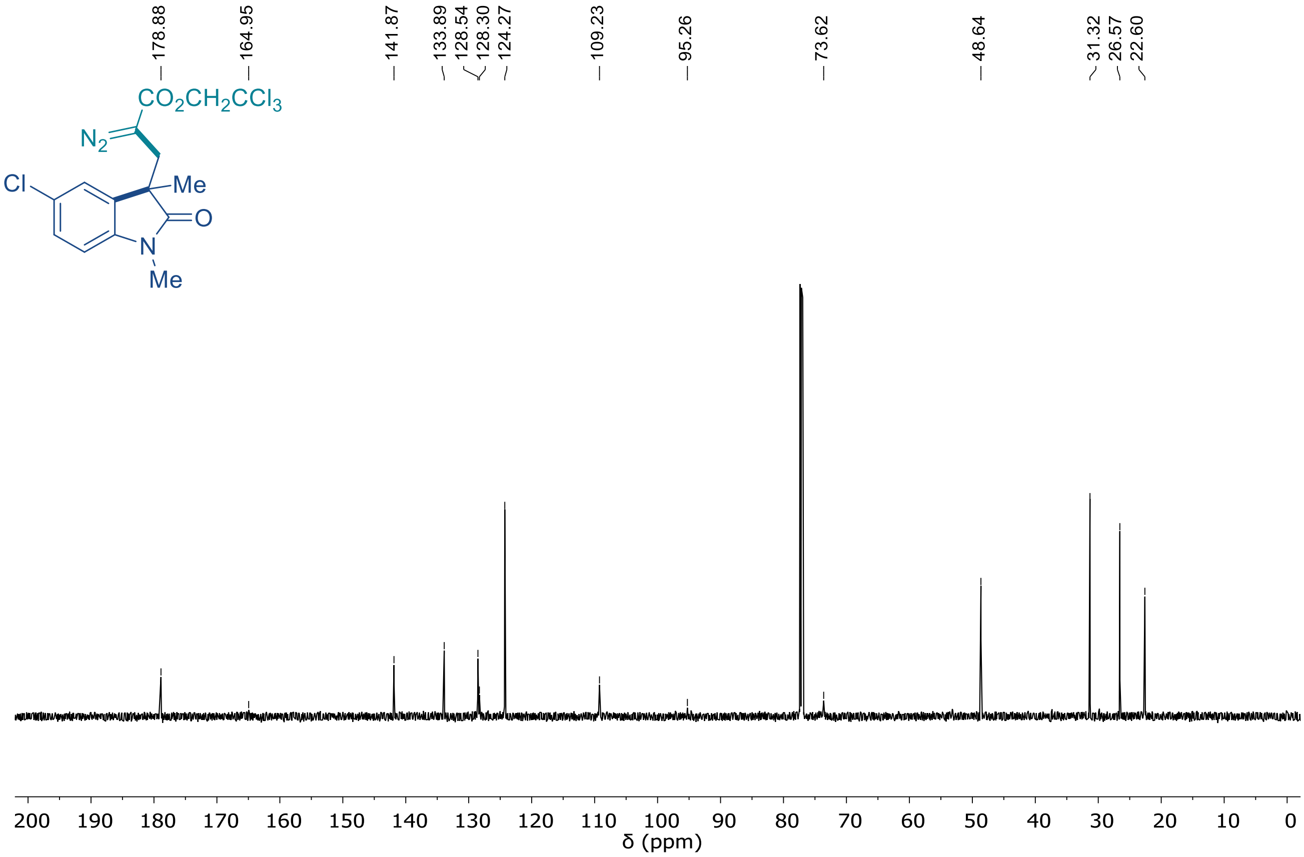

^{13}C NMR (151 MHz, $CDCl_3$) of compound **3f**

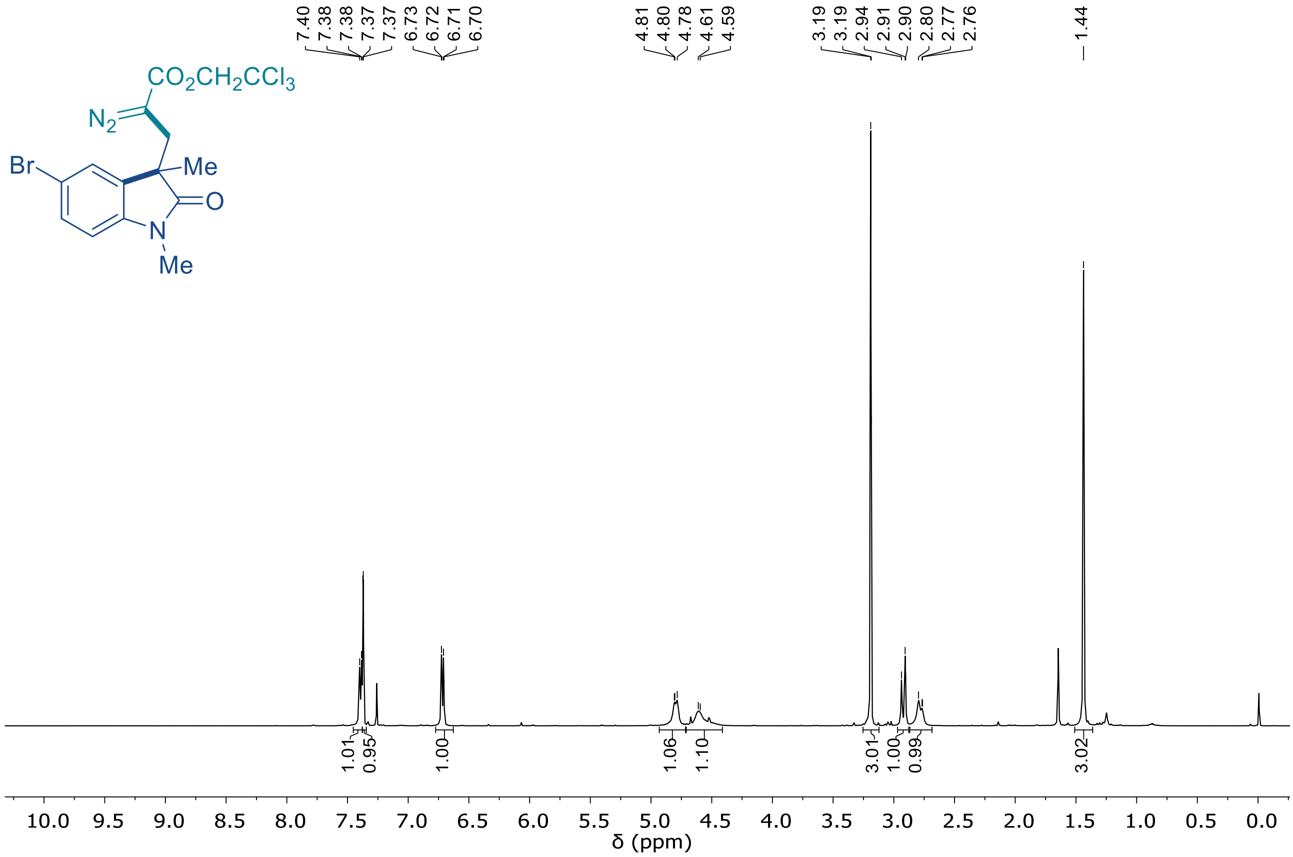

^{1}H NMR (500 MHz, $CDCl_3$) of compound **3g**

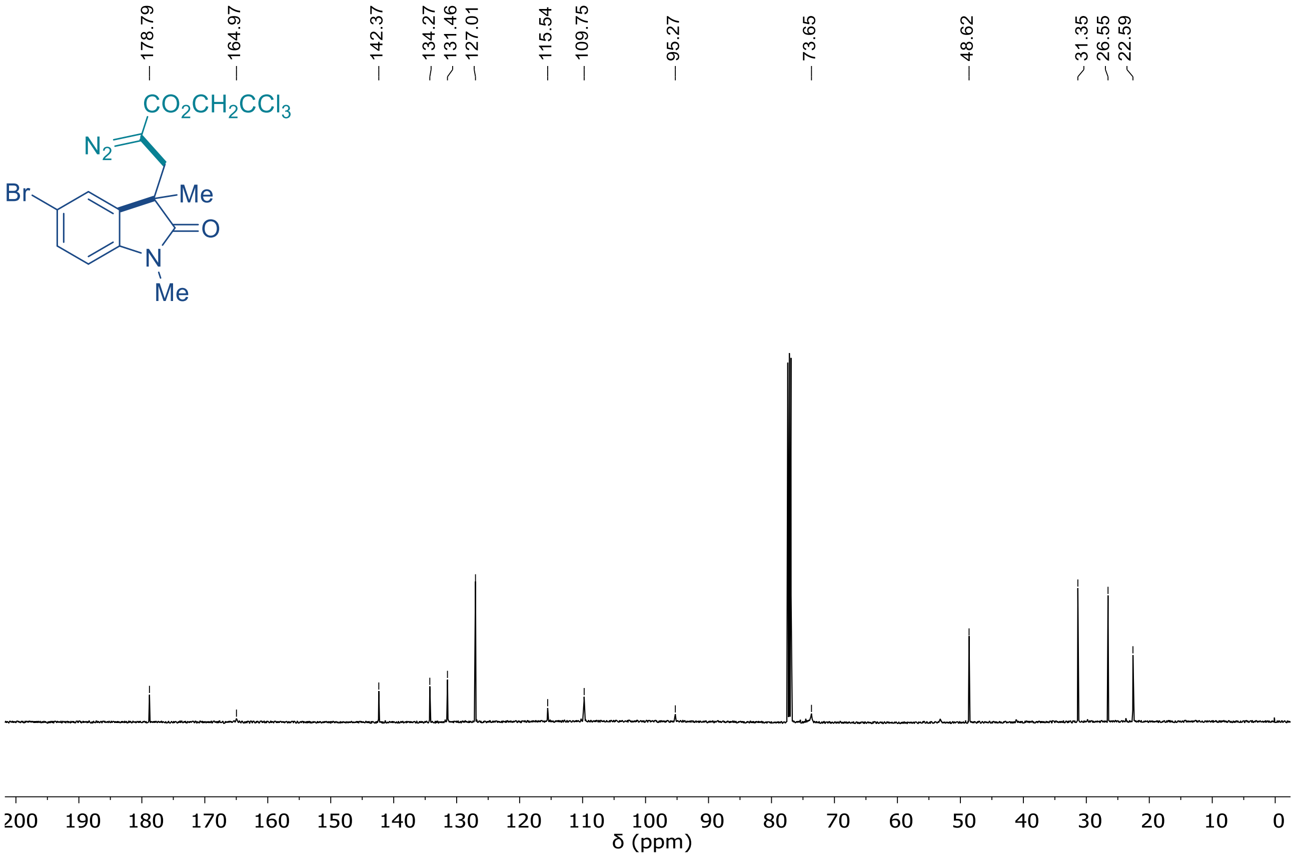

^{13}C NMR (151 MHz, $CDCl_3$) of compound **3g**

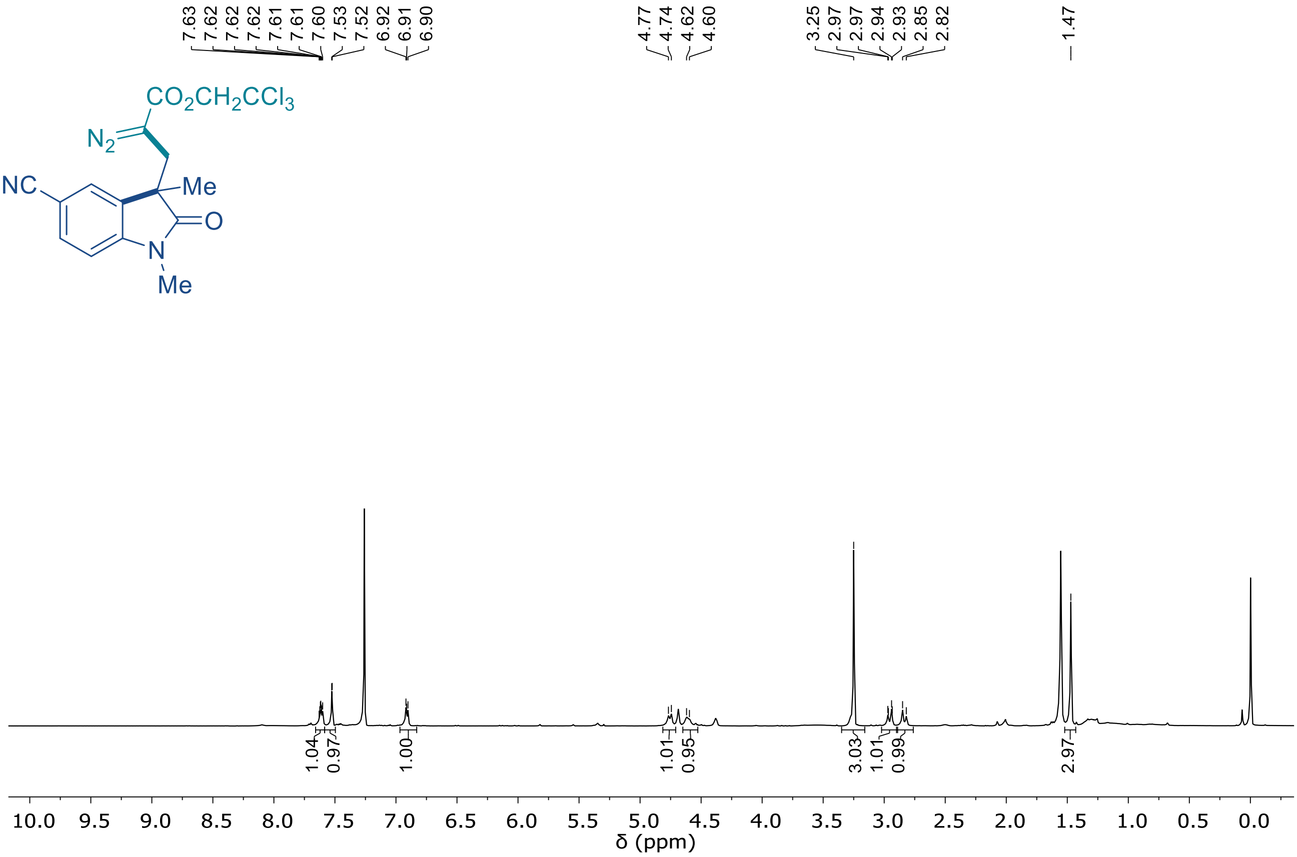

^{1}H NMR (500 MHz, $CDCl_3$) of compound **3h**

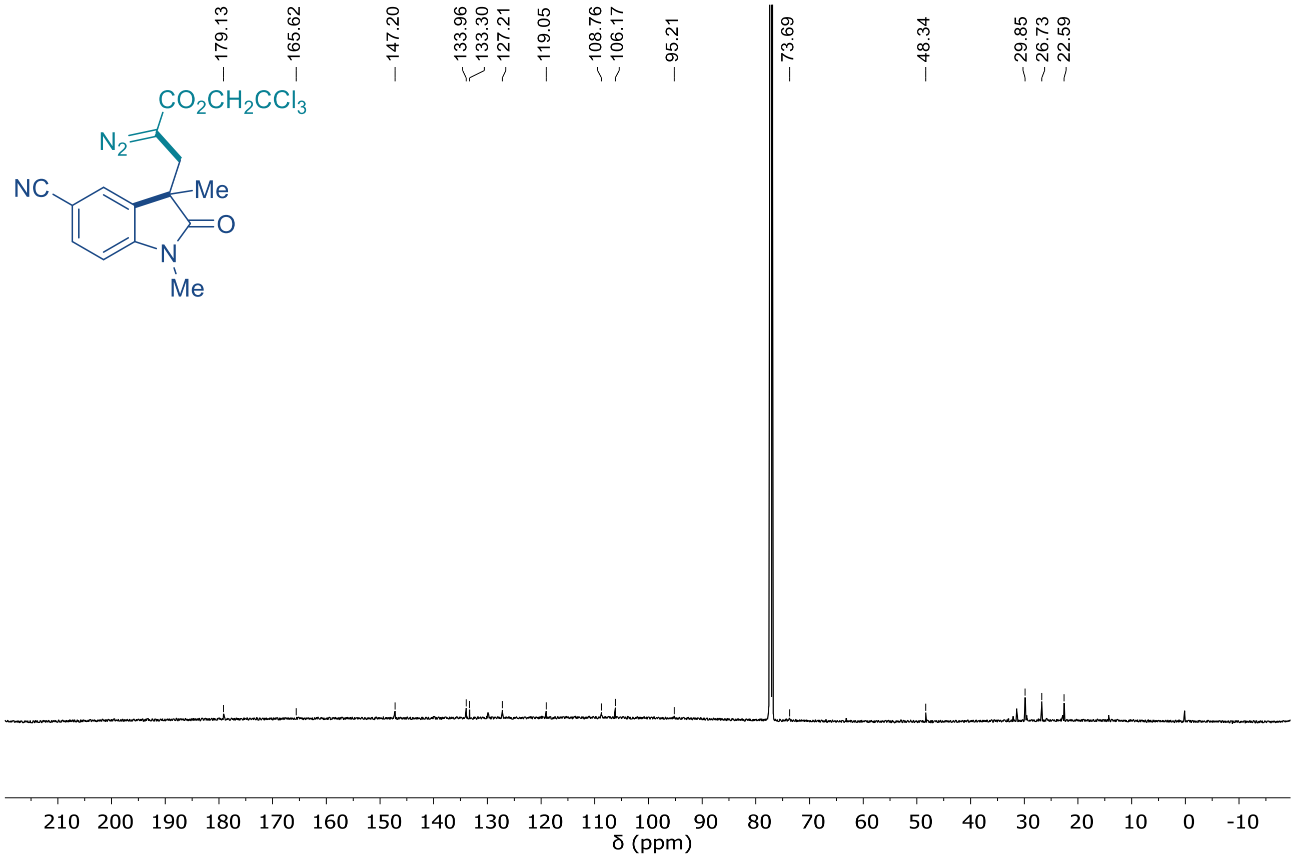

^{13}C NMR (126 MHz, $CDCl_3$) of compound **3h**

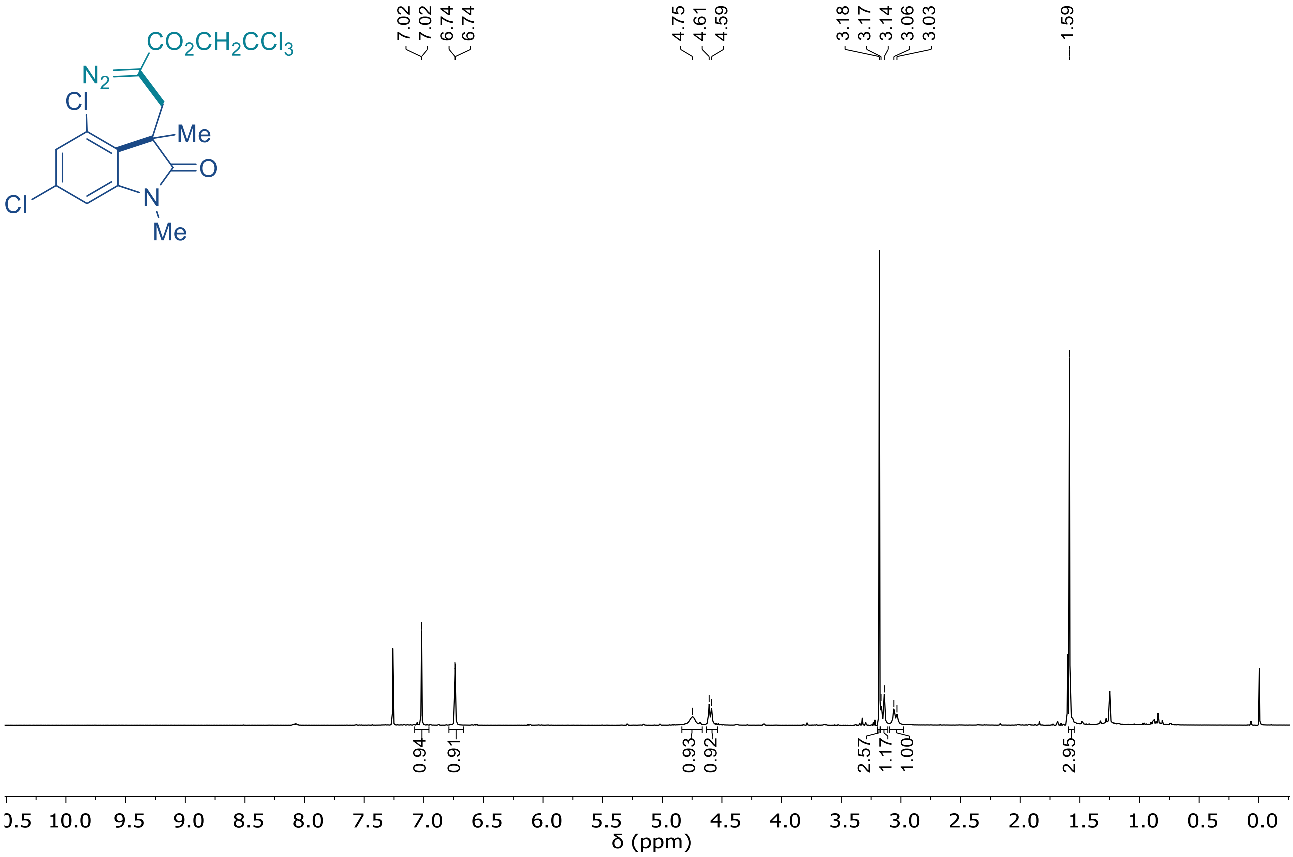

^{1}H NMR (600 MHz, $CDCl_3$) of compound **3i**

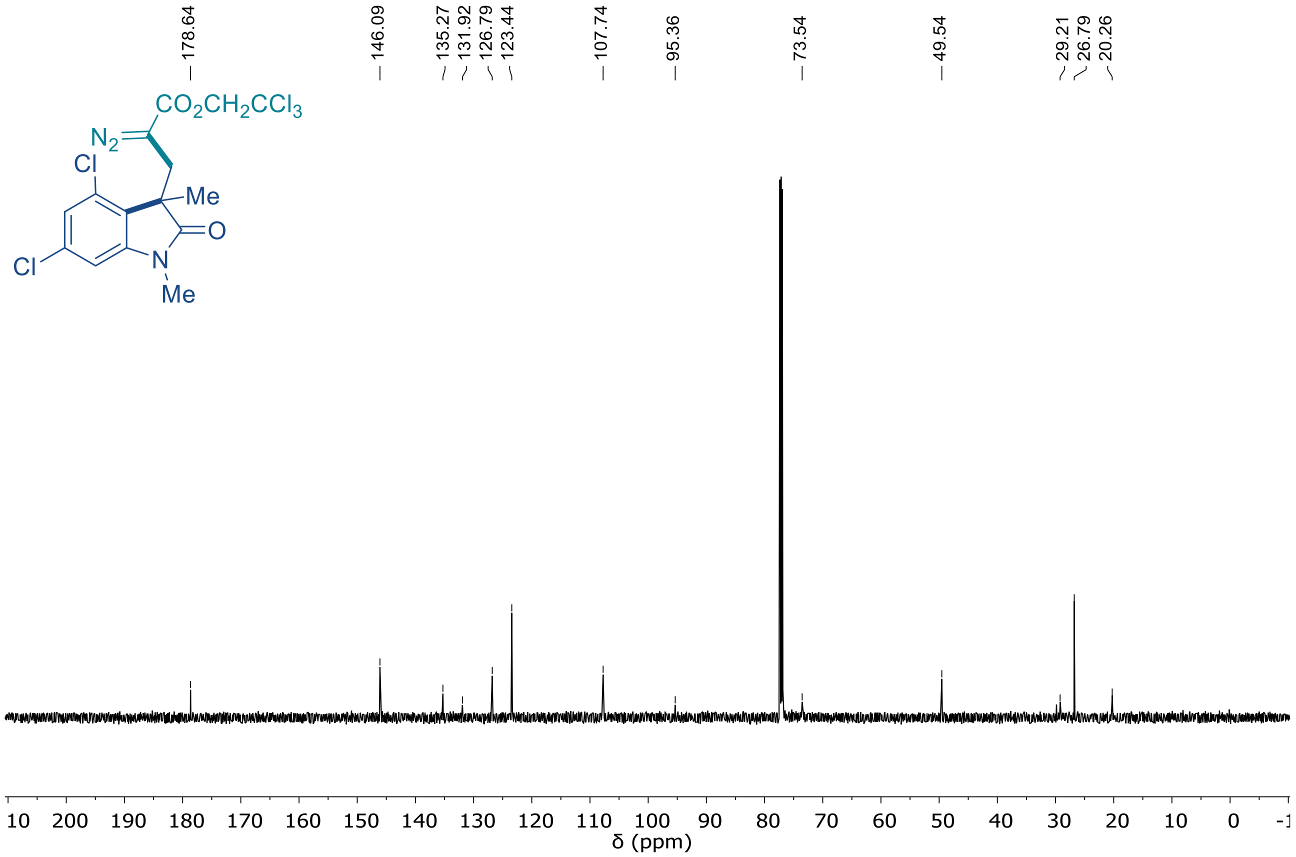

^{13}C NMR (151 MHz, $CDCl_3$) of compound **3i**

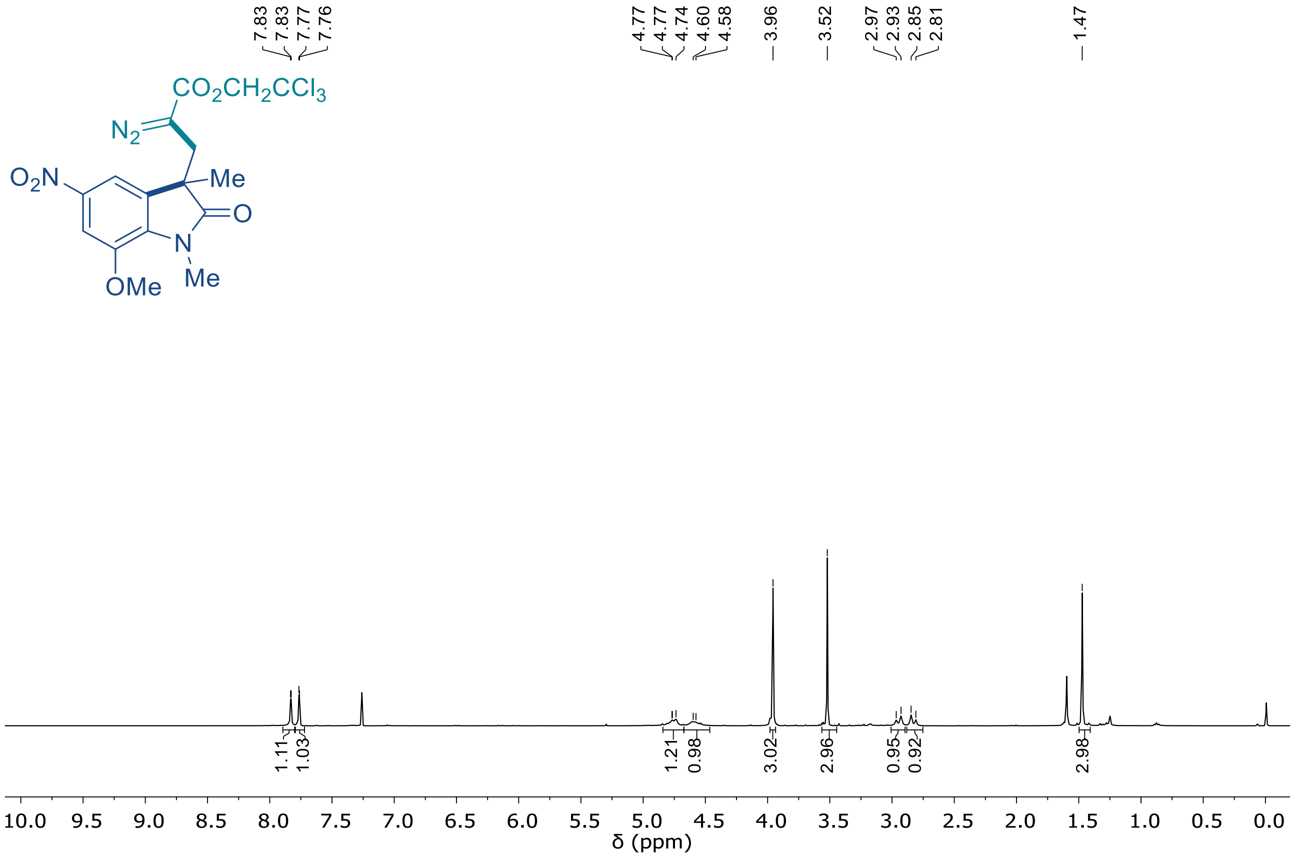

^{1}H NMR (400 MHz, $CDCl_3$) of compound **3j**

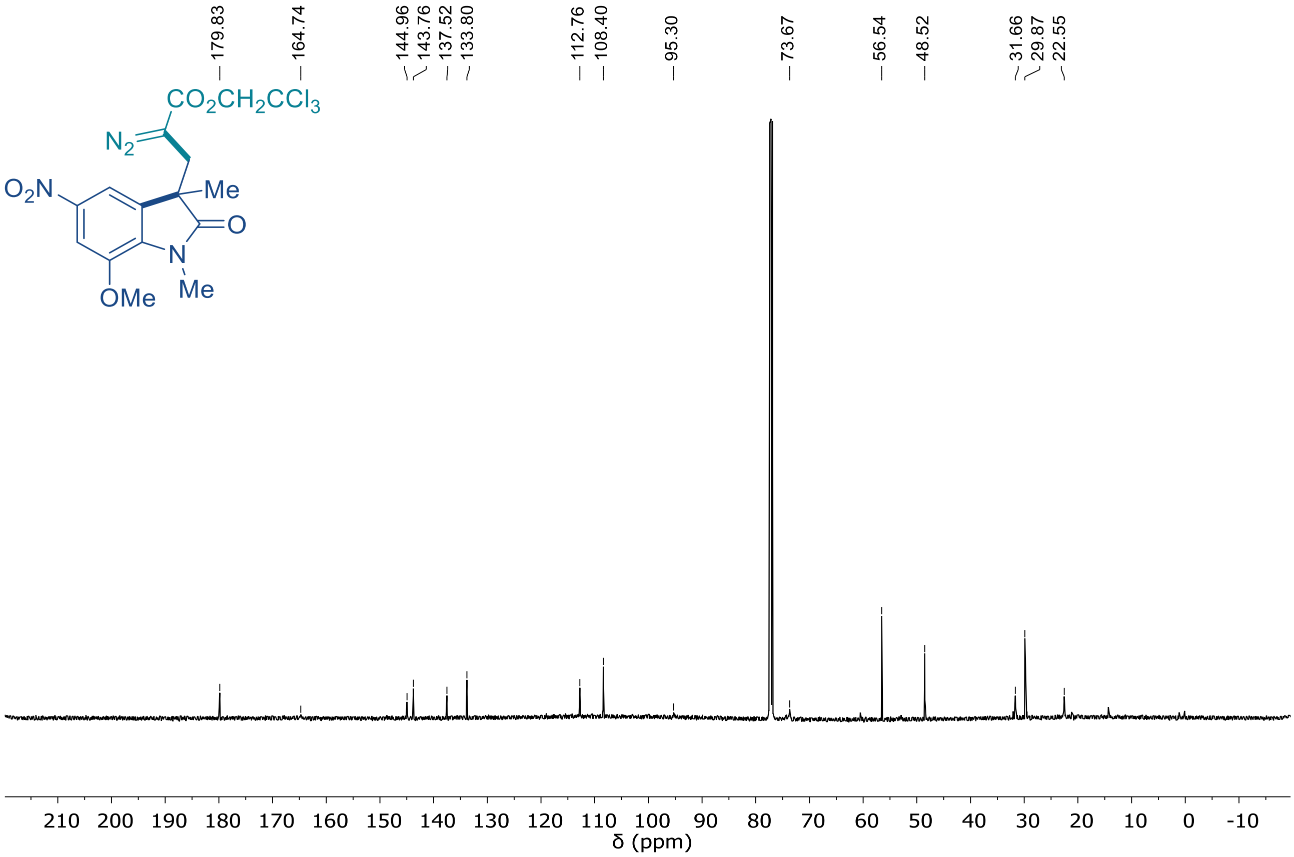

^{13}C NMR (101 MHz, $CDCl_3$) of compound **3j**

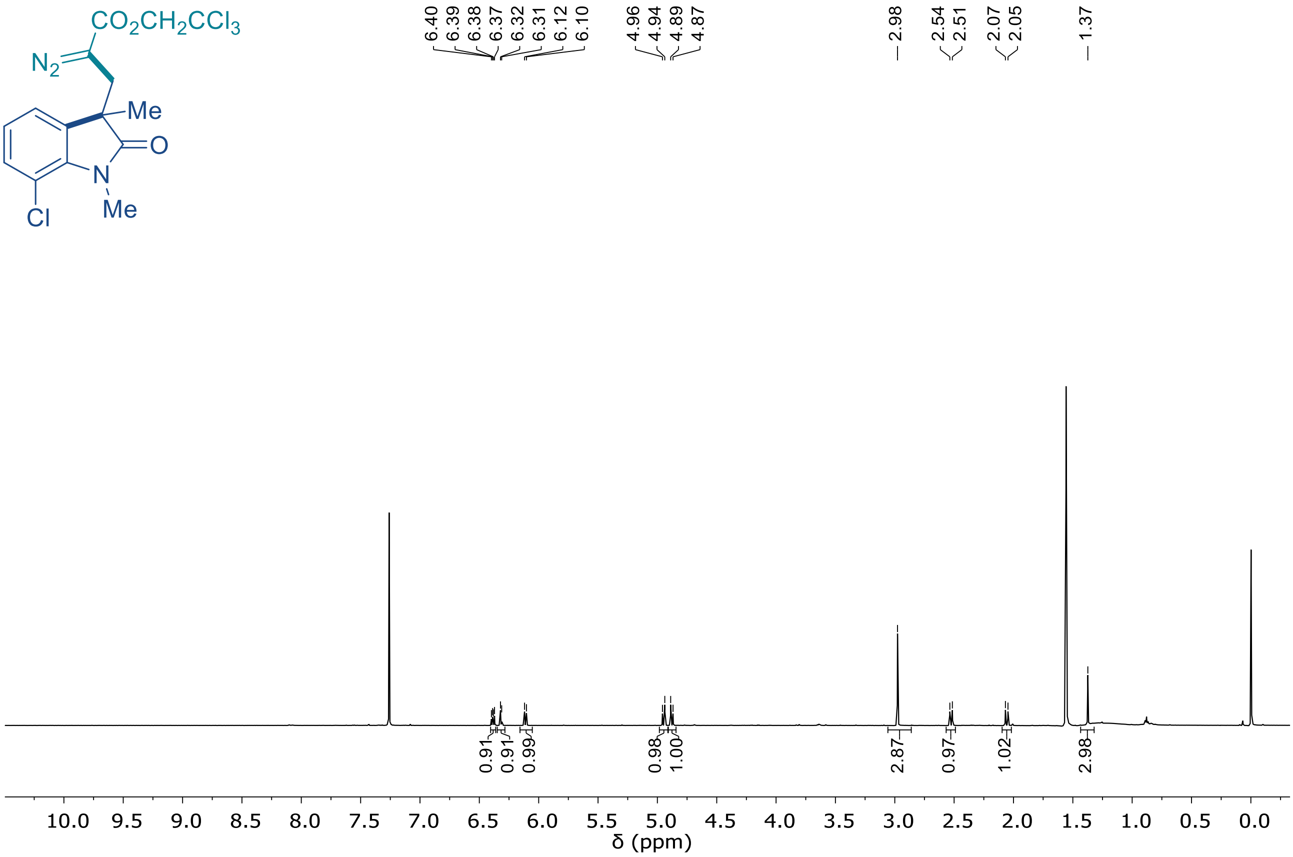

^{1}H NMR (500 MHz, $CDCl_3$) of compound **3k**

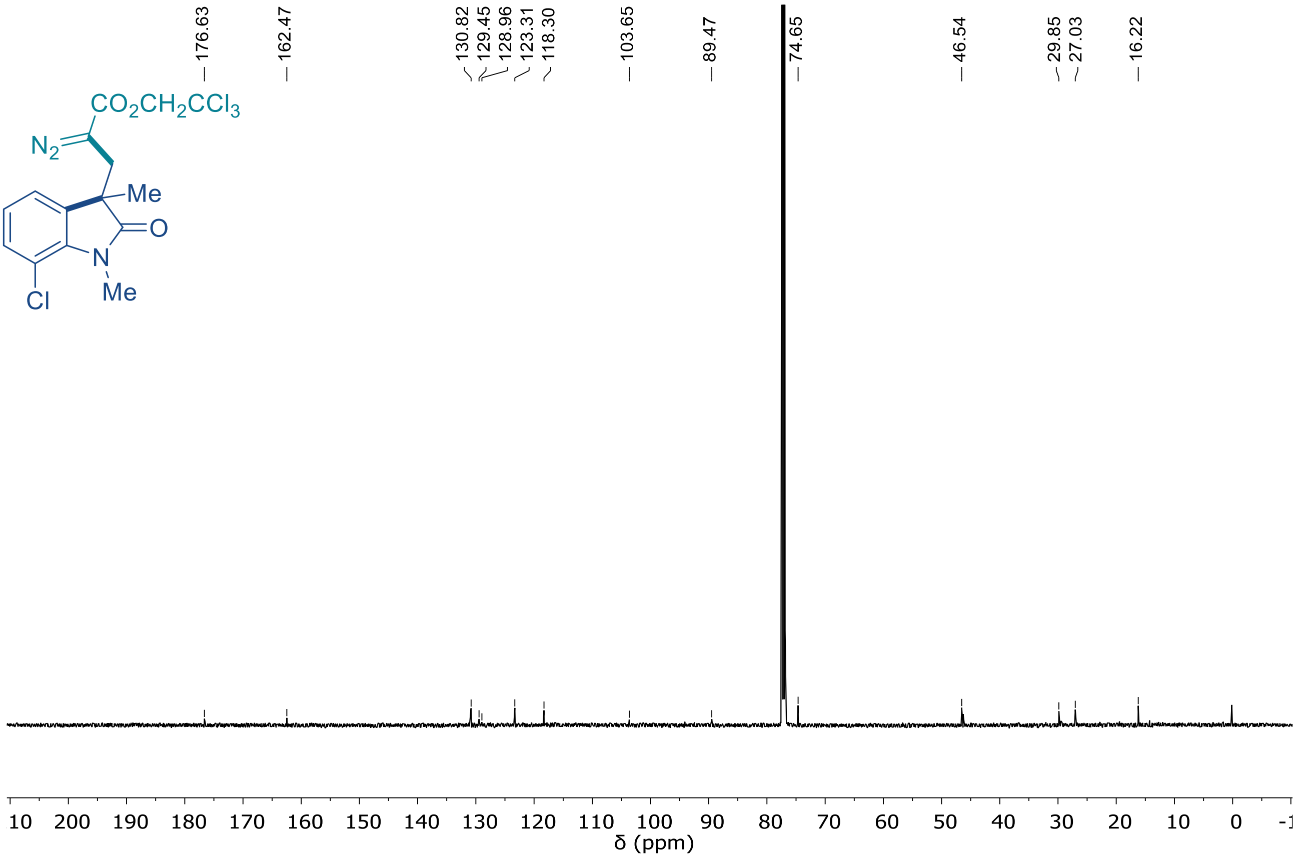

^{13}C NMR (151 MHz, $CDCl_3$) of compound **3k**

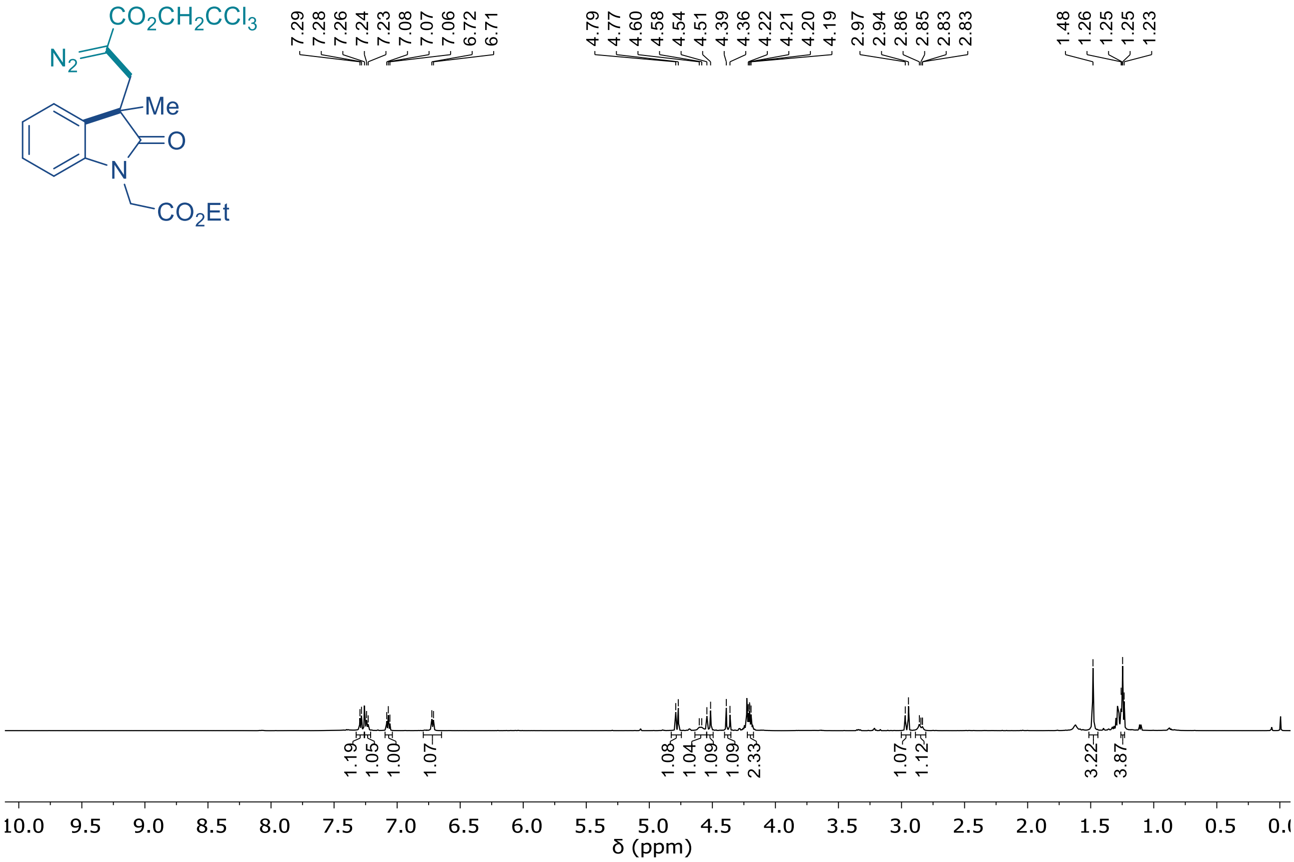

^{1}H NMR (600 MHz, $CDCl_3$) of compound **3l**

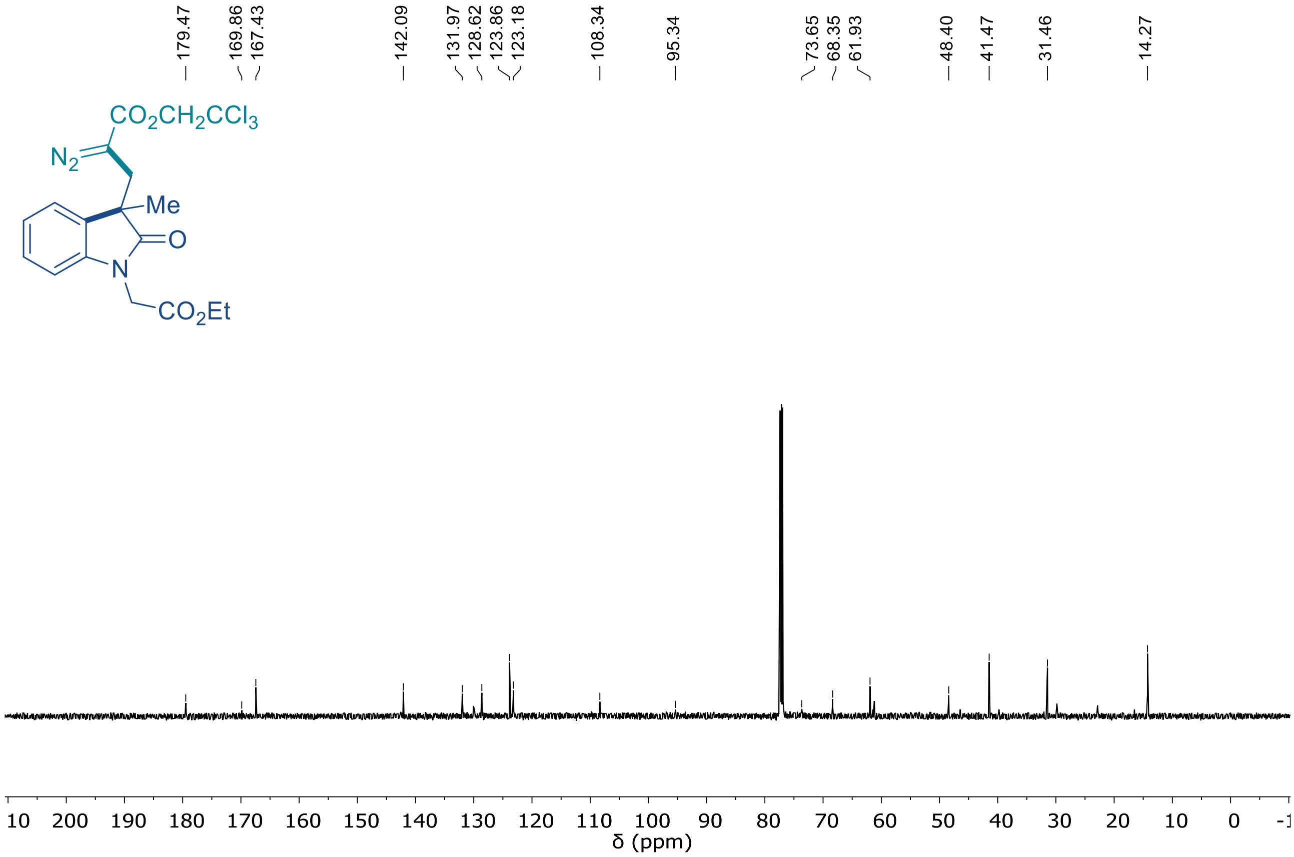

^{13}C NMR (151 MHz, $CDCl_3$) of compound **3l**

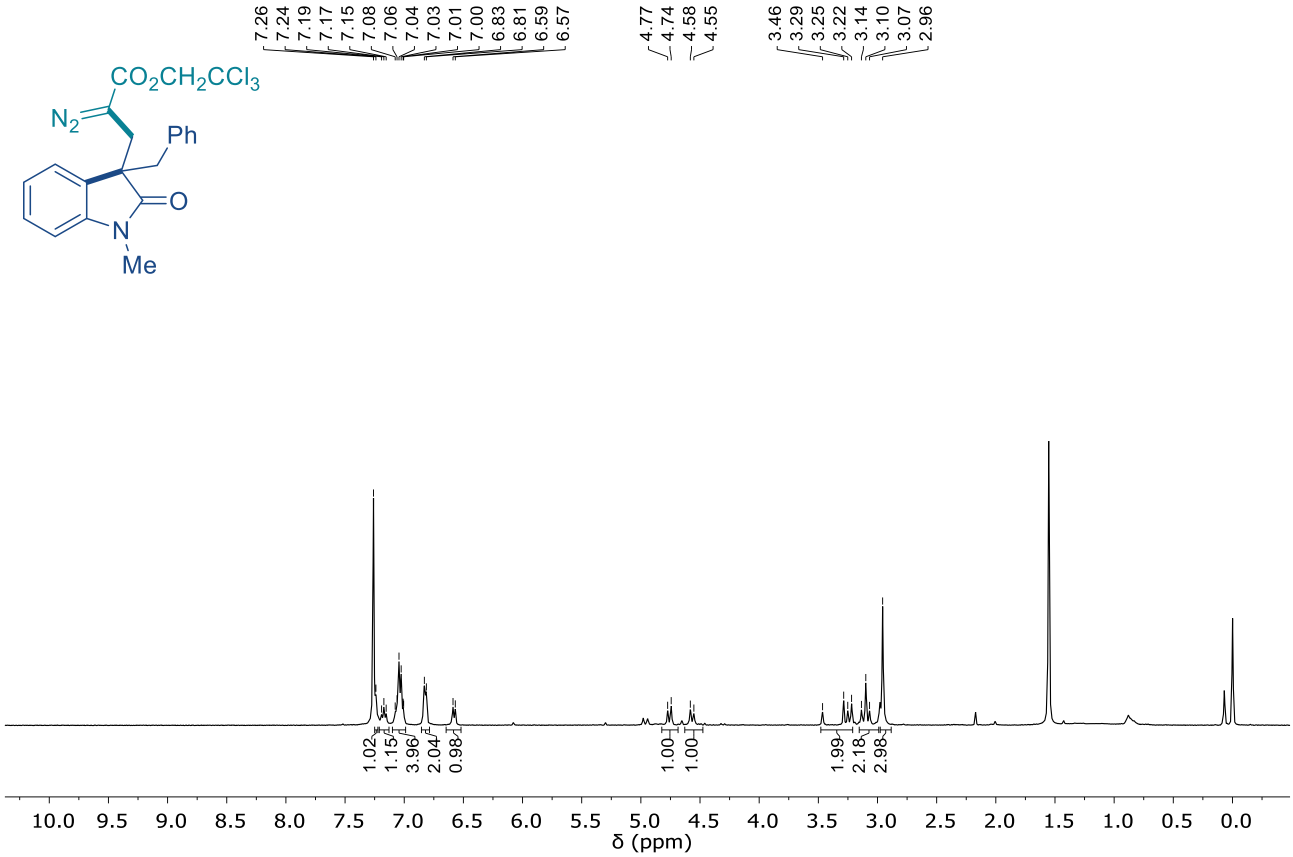

^{1}H NMR (400 MHz, $CDCl_3$) of compound **3m**

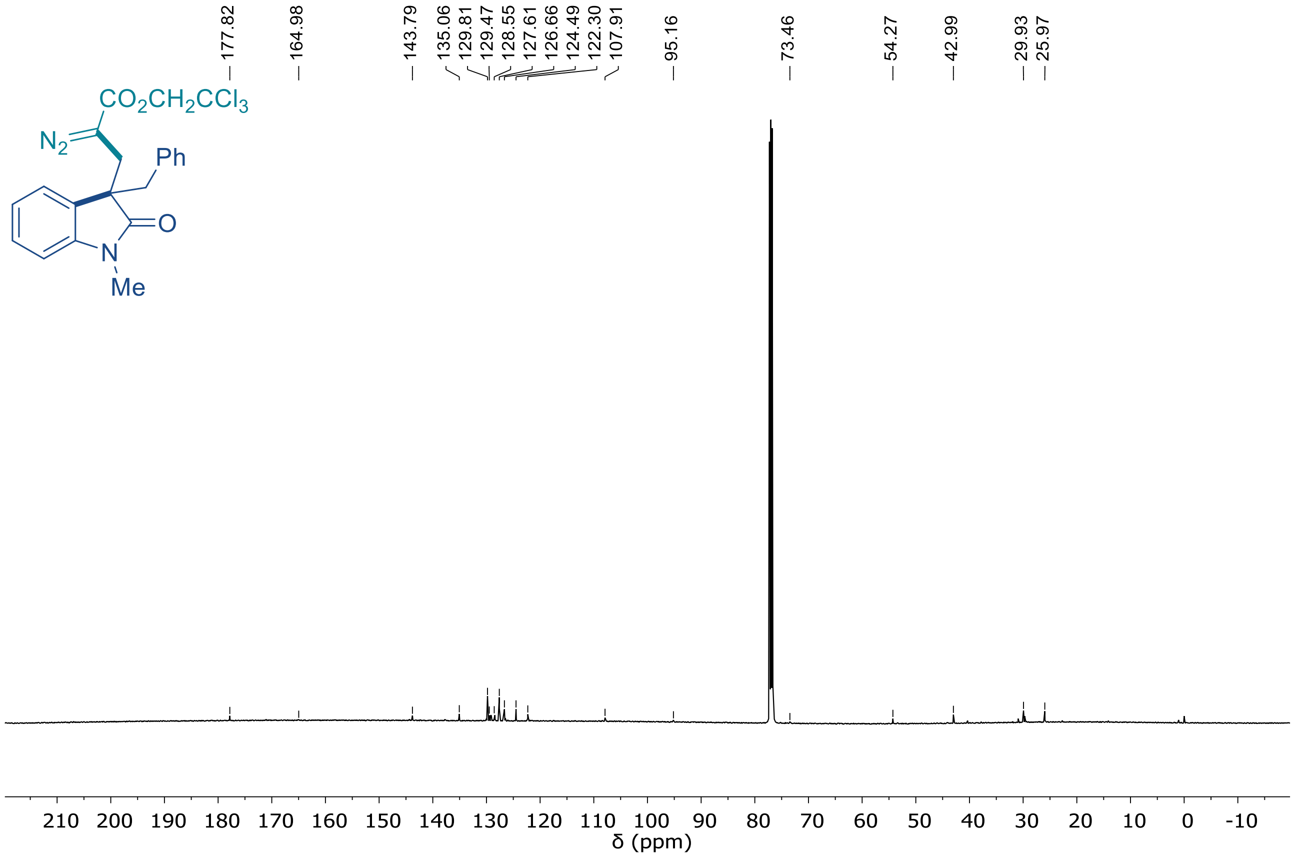

^{13}C NMR (151 MHz, CDCl3) of compound **3m**

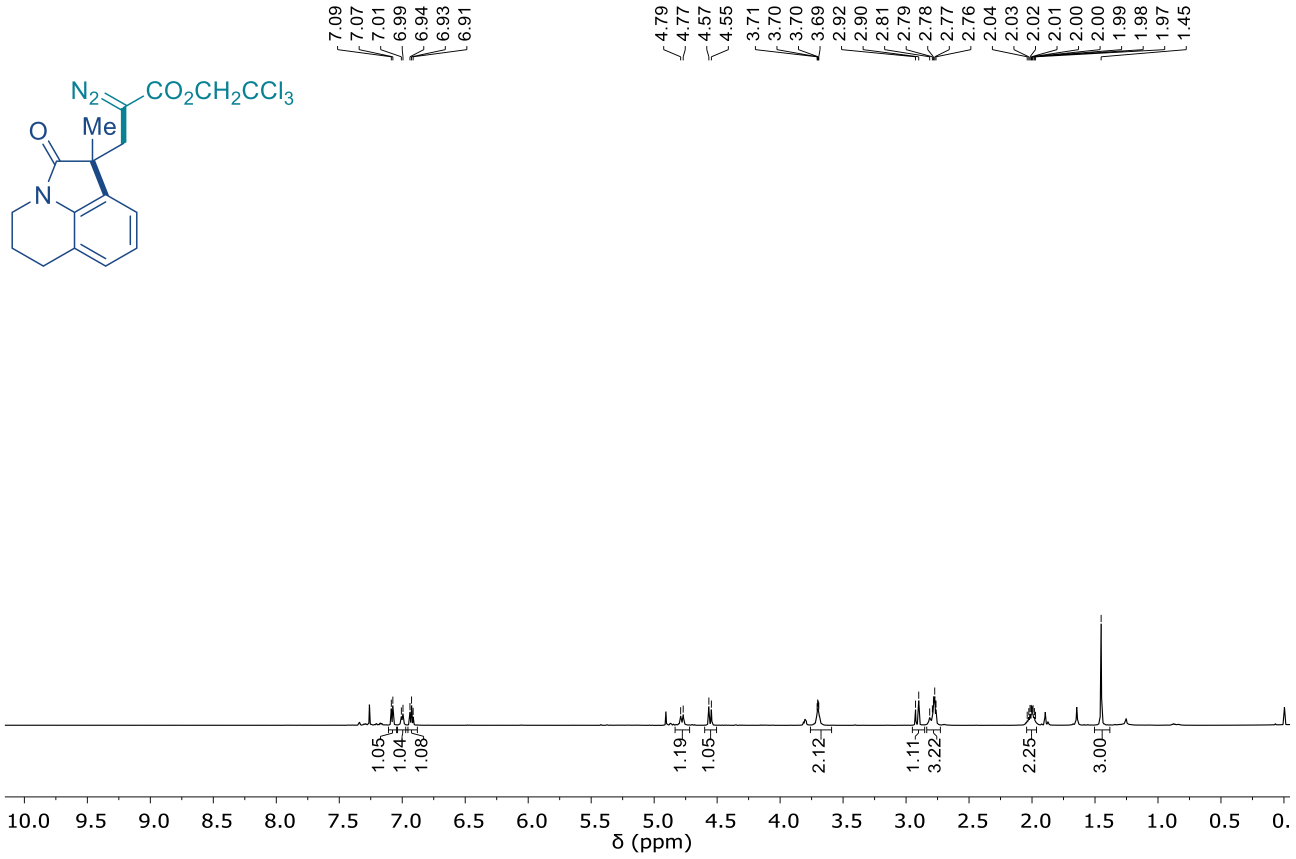

^{1}H NMR (600 MHz, CDCl3) of compound **3n**

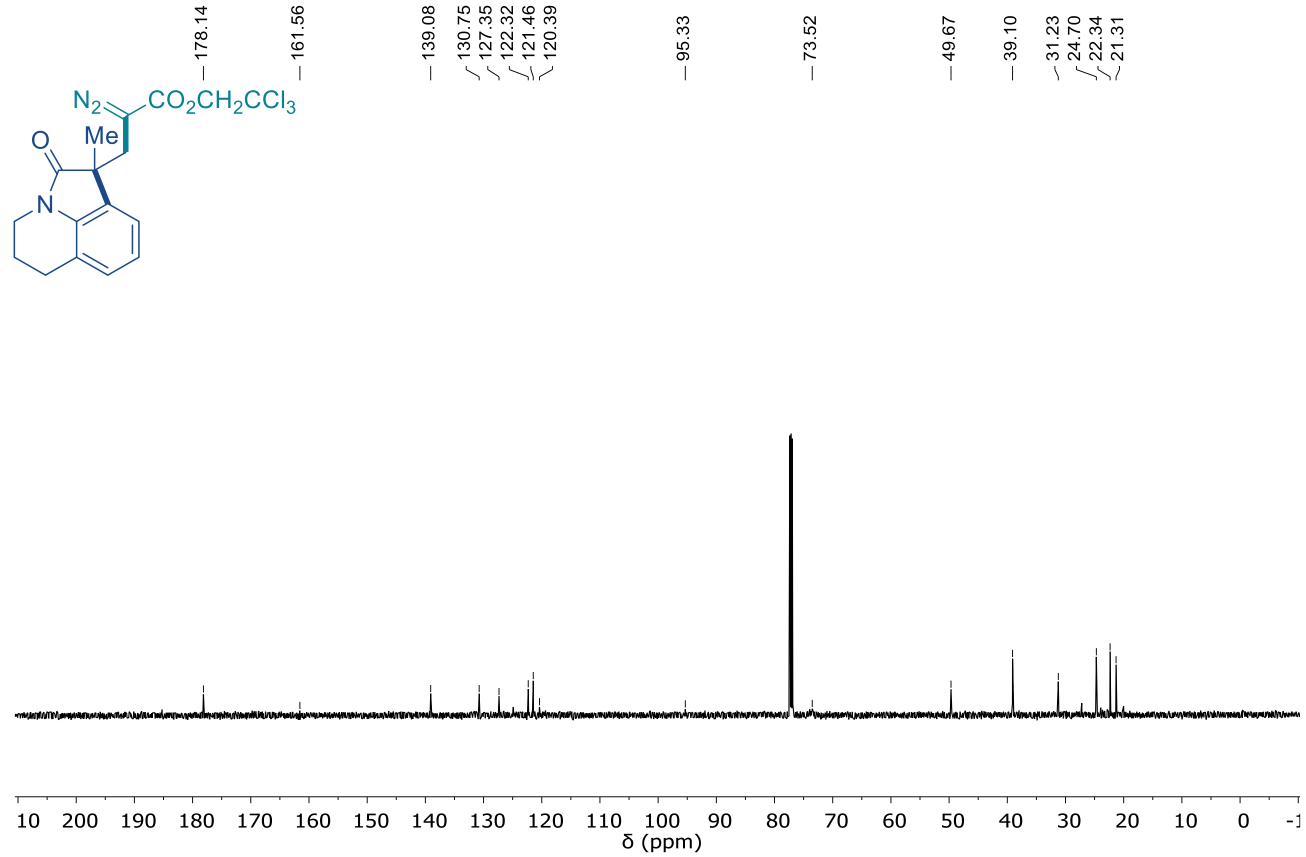

^{13}C NMR (151 MHz, $CDCl_3$) of compound **3n**

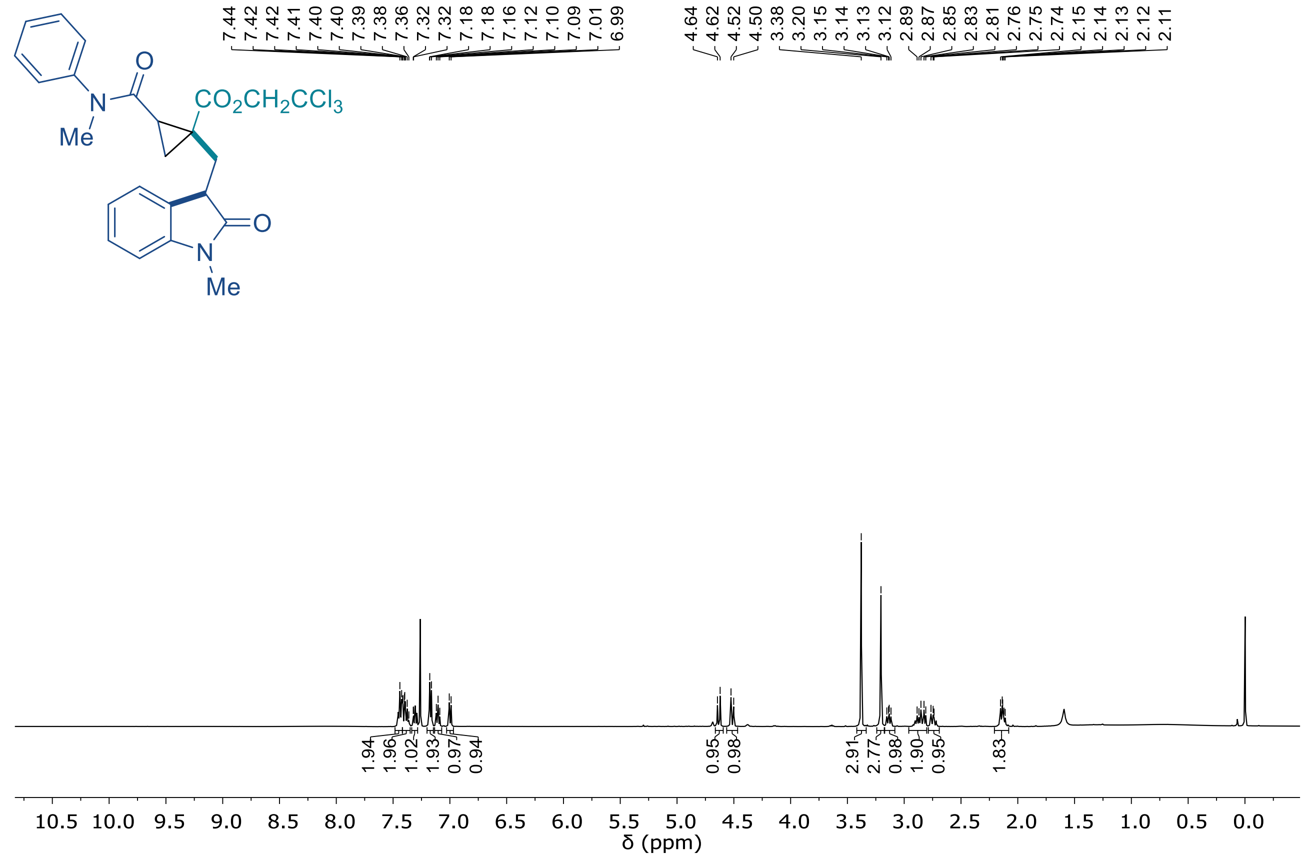

^{1}H NMR (400 MHz, $CDCl_3$) of compound **3o**

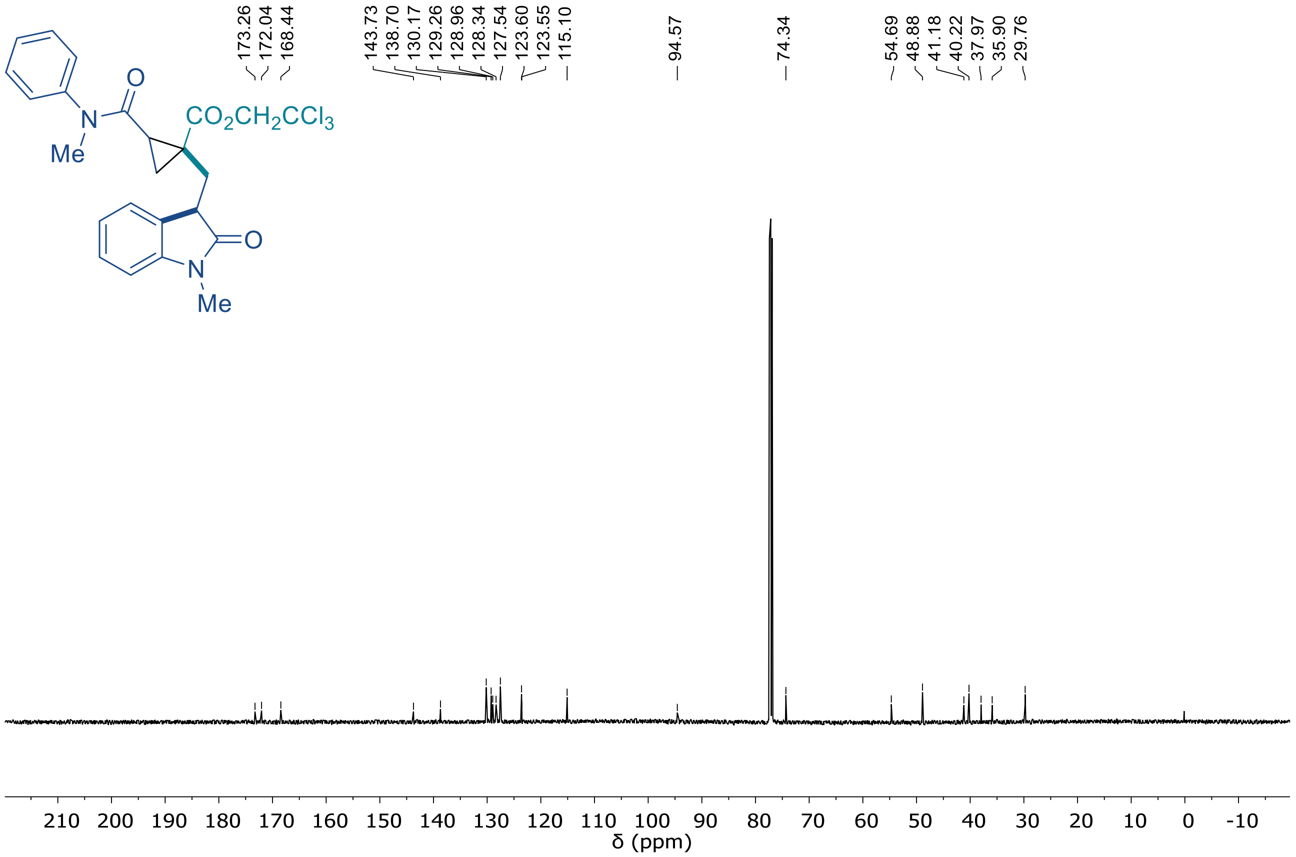

^{13}C NMR (151 MHz, $CDCl_3$) of compound **3o**

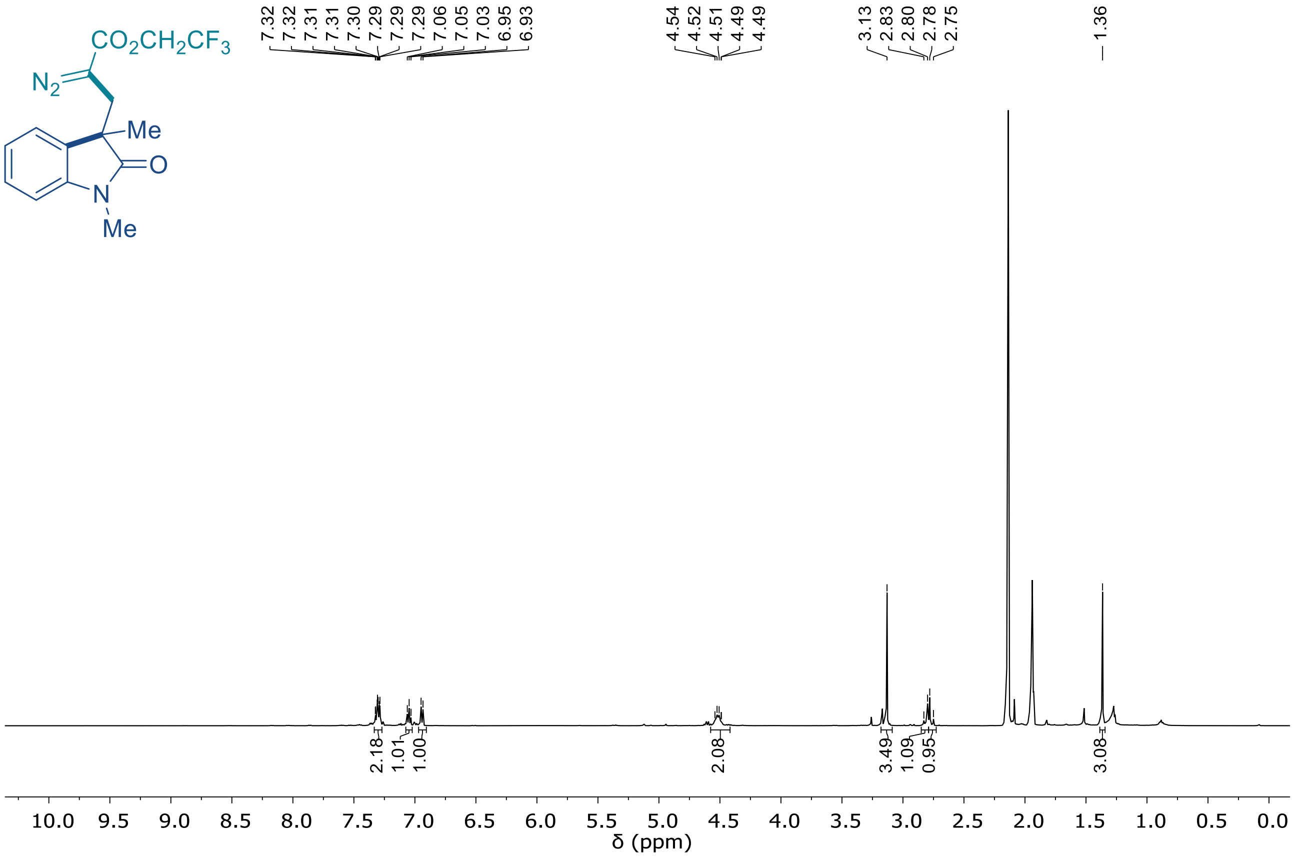

^{1}H NMR (500 MHz, CD_3CN) of compound **3p**

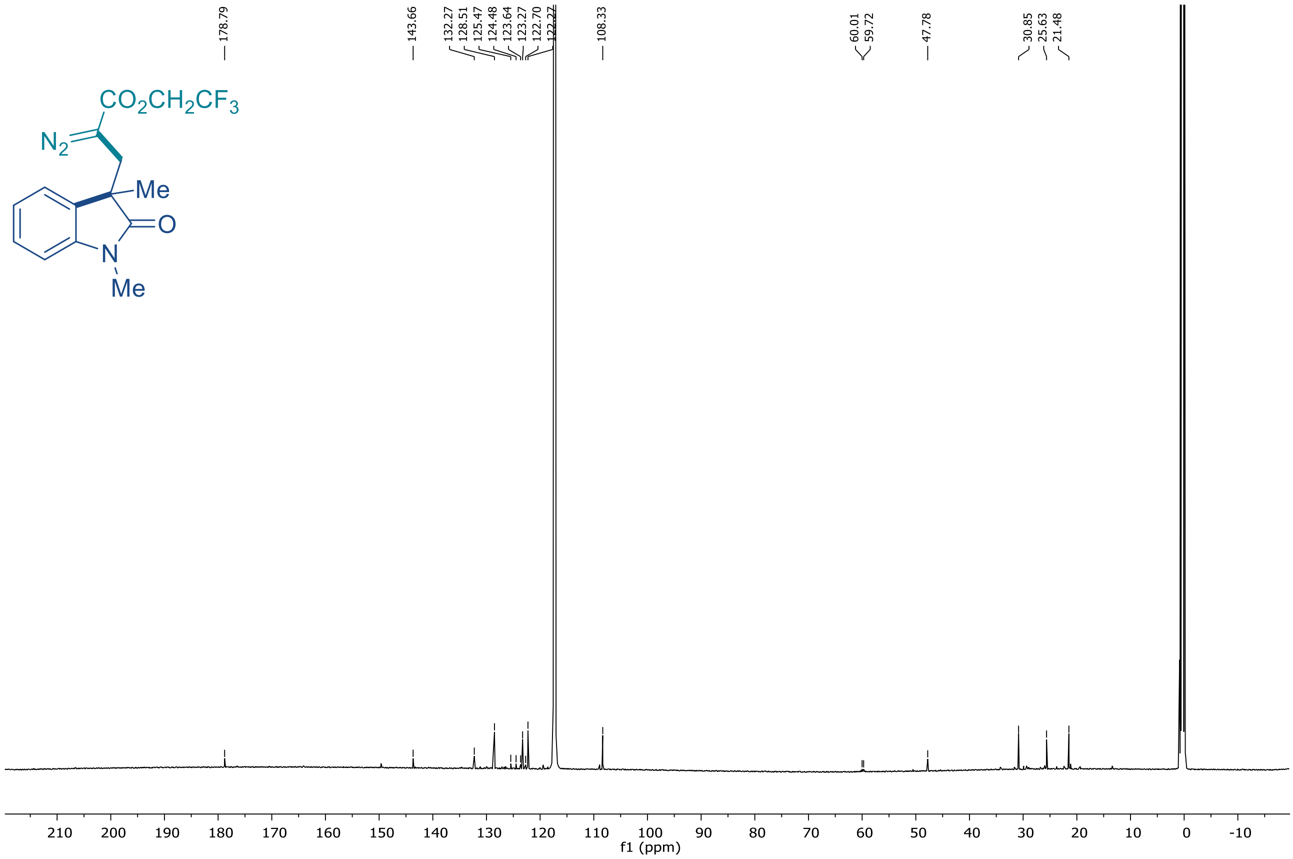

^{13}C NMR (126 MHz, CD_3CN) of compound **3p**

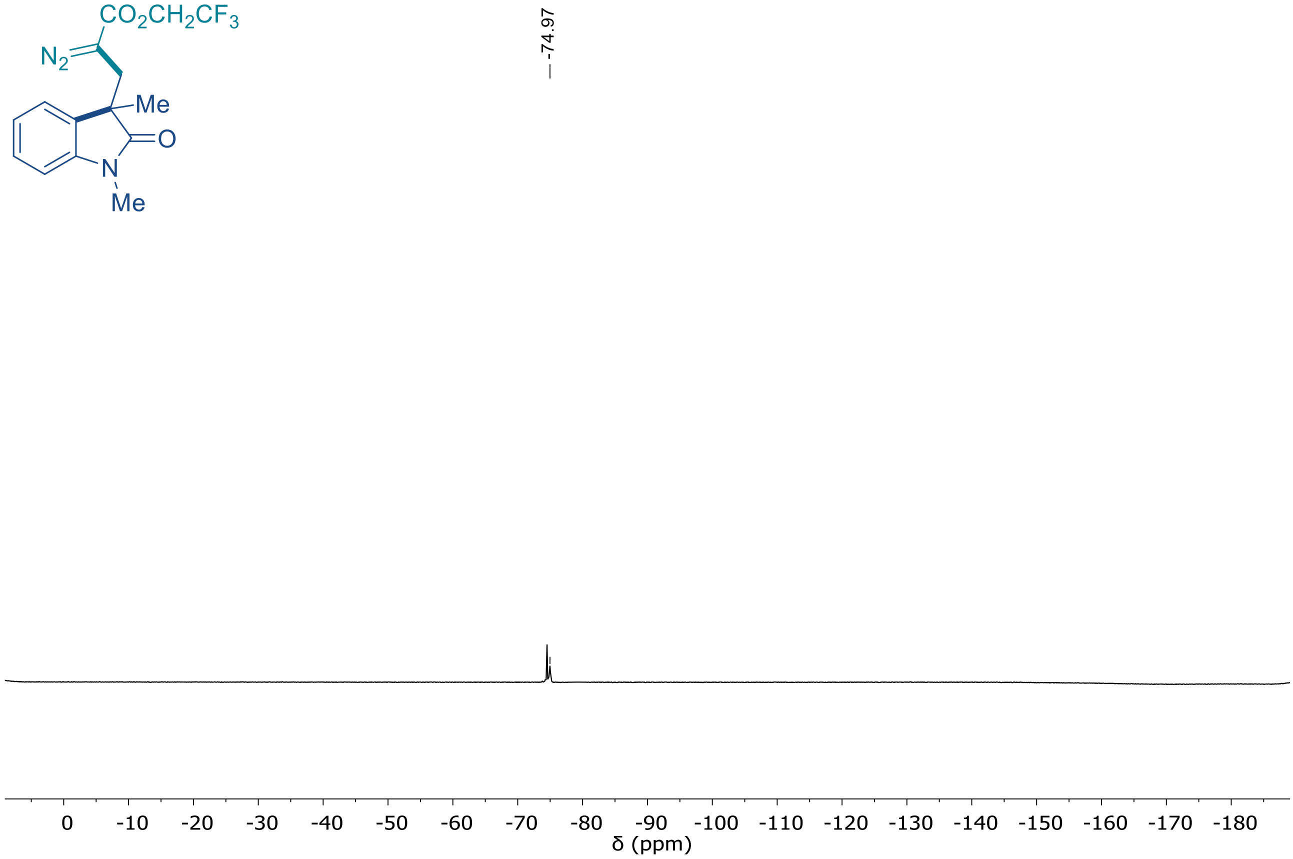

^{19}F NMR (377 MHz, CD_3CN) of compound **3p**

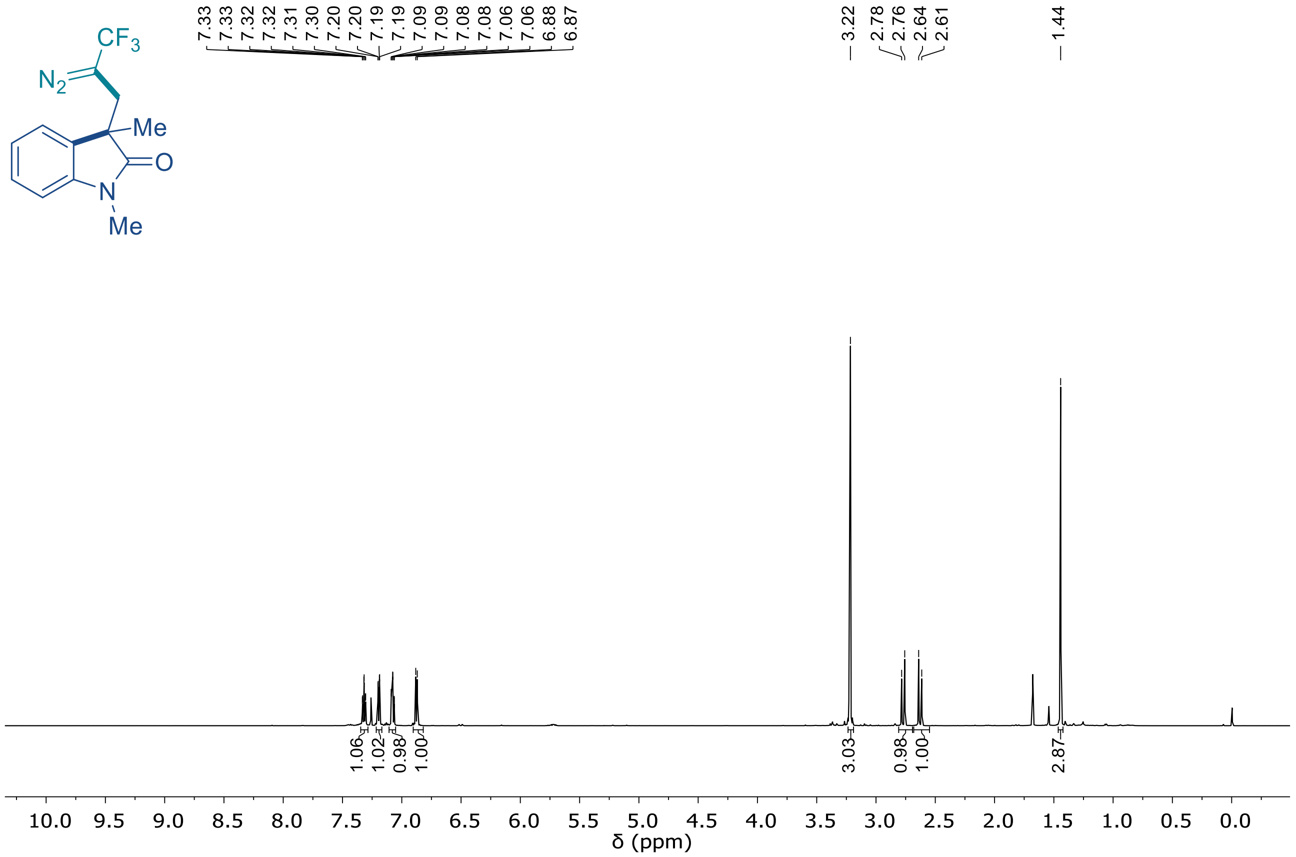

^{1}H NMR (600 MHz, $CDCl_3$) of compound **3q**

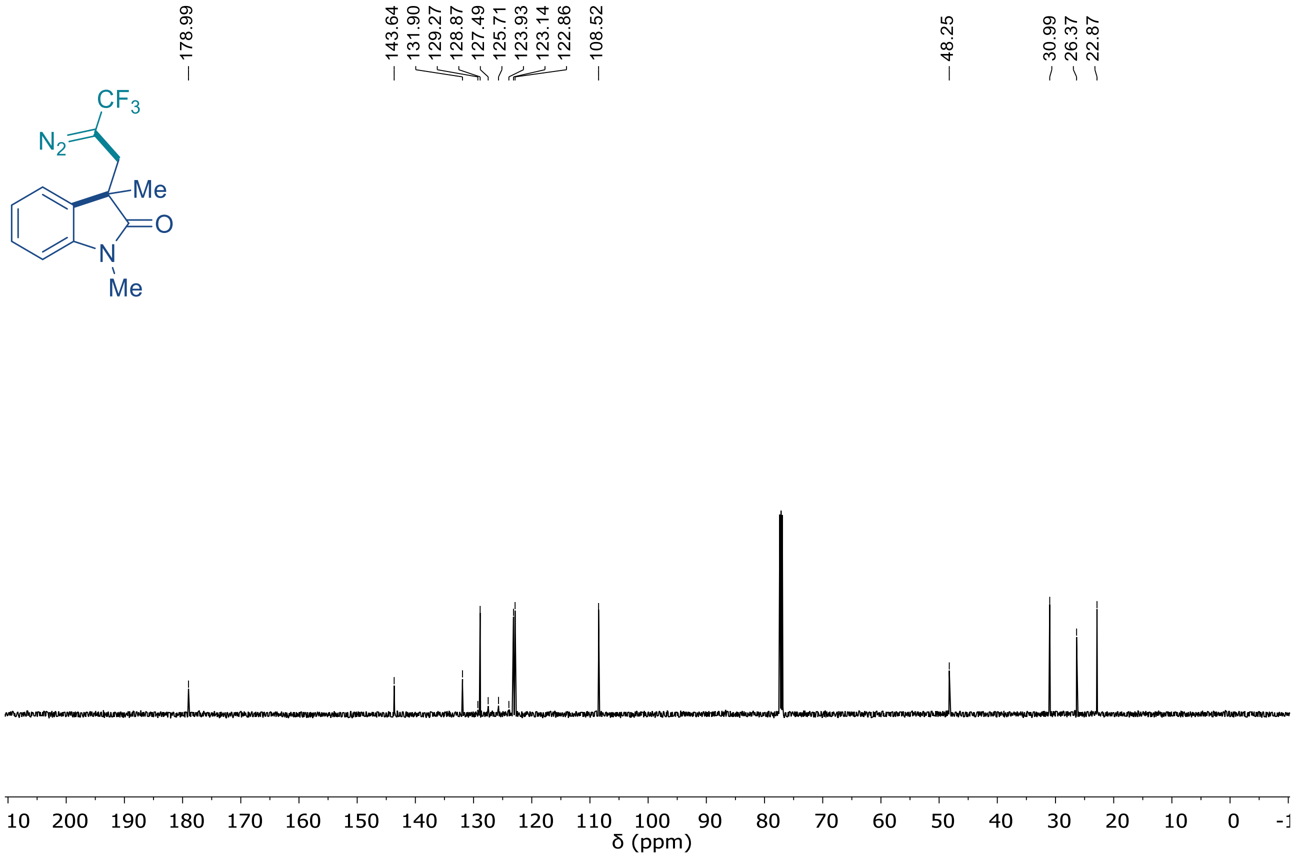

^{13}C NMR (151 MHz, $CDCl_3$) of compound **3q**

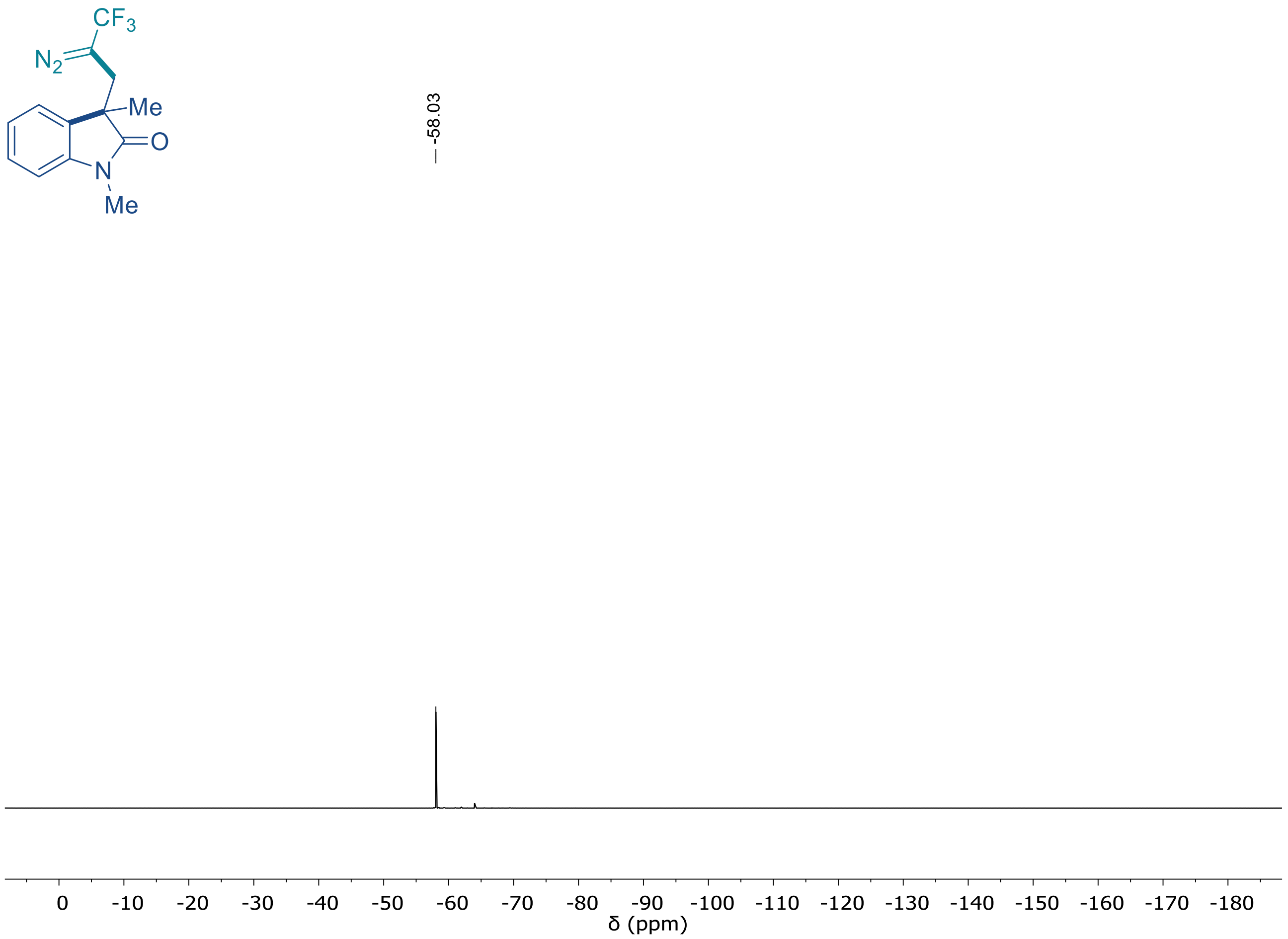

^{19}F NMR (282 MHz, $CDCl_3$) of compound **3q**

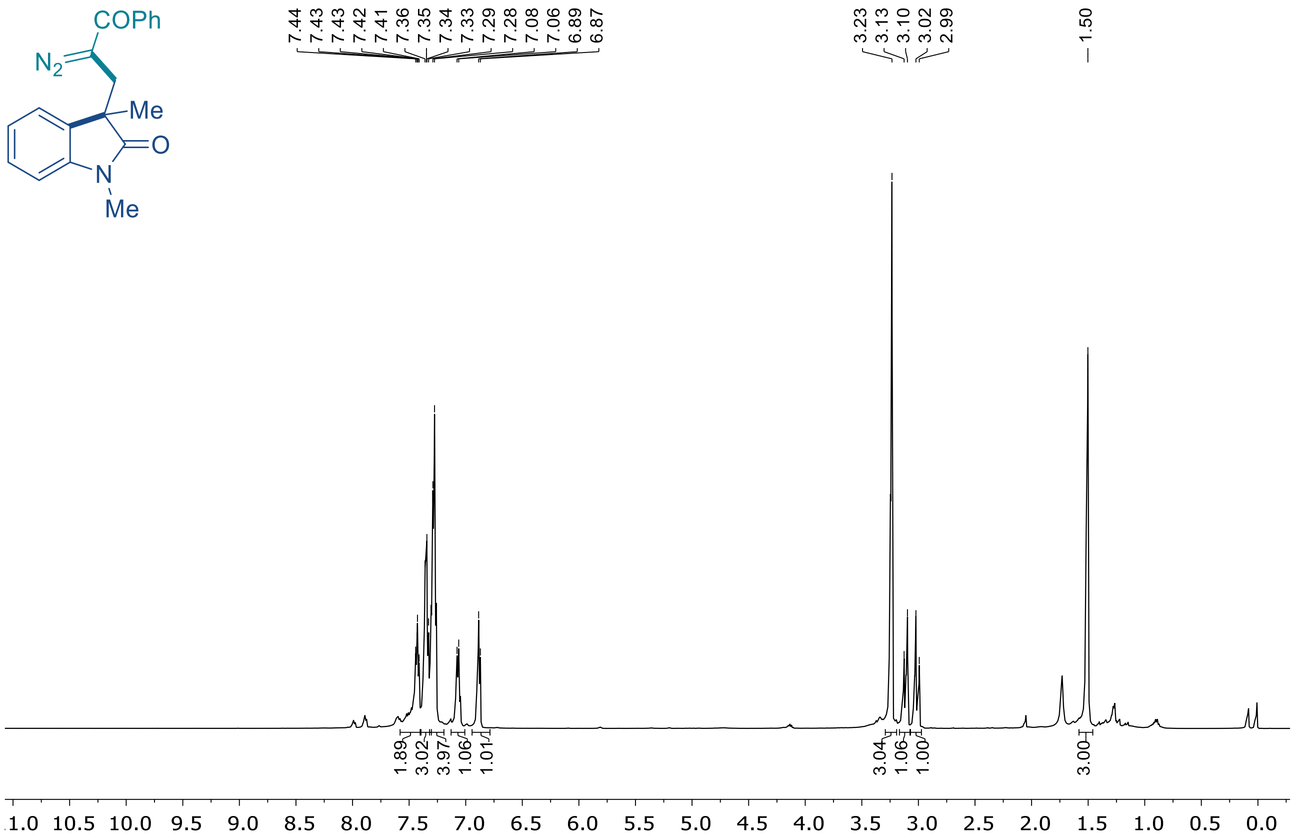

^{1}H NMR (500 MHz, $CDCl_3$) of compound **3r**

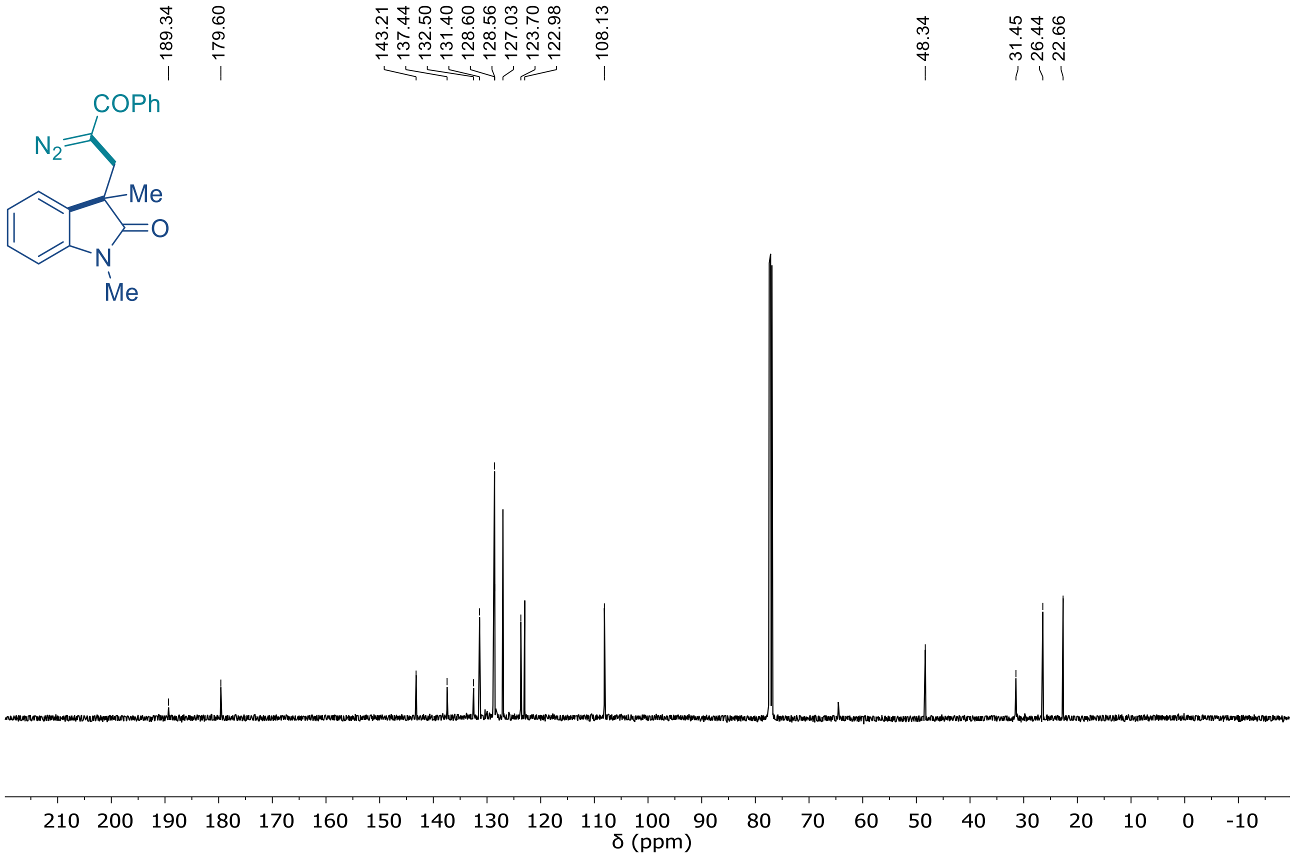

^{13}C NMR (126 MHz, $CDCl_3$) of compound **3r**

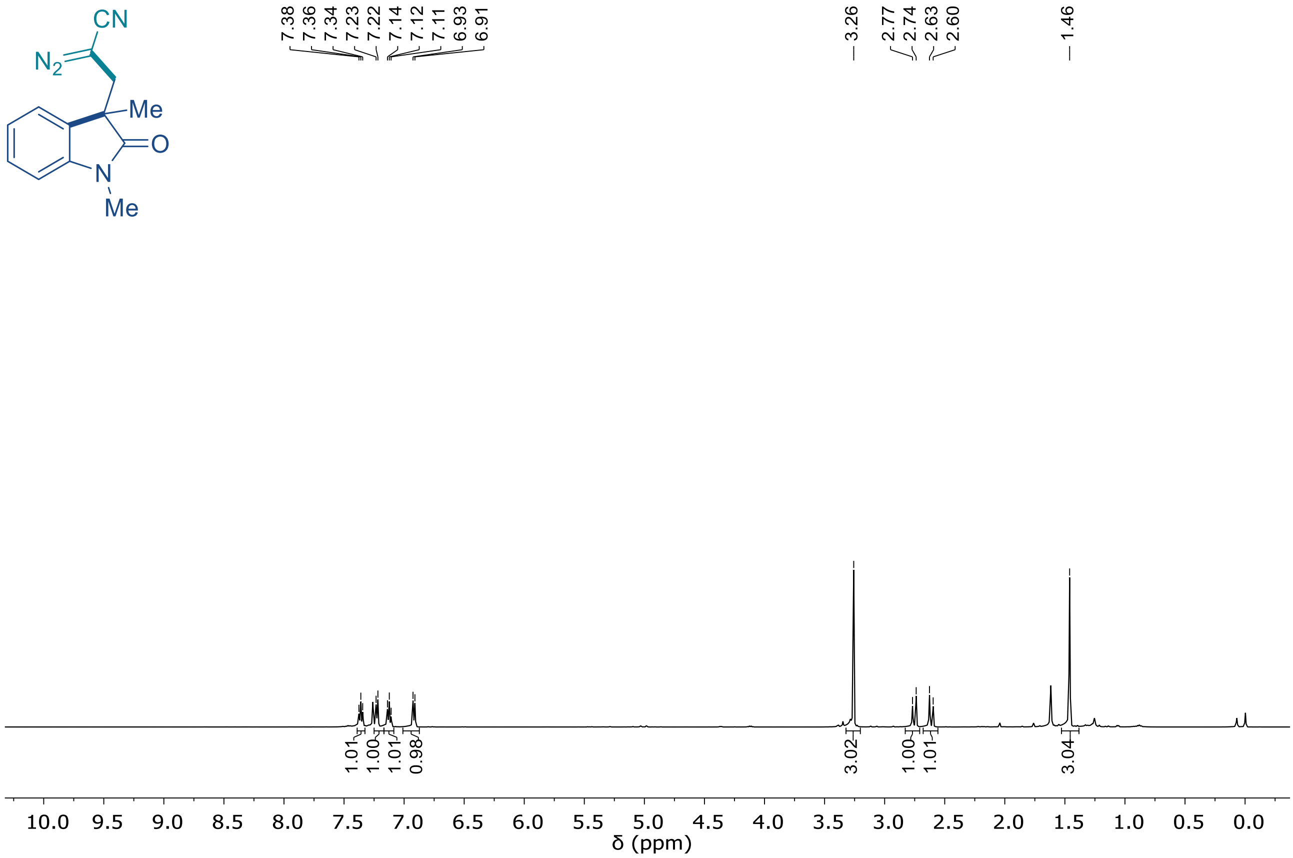

^{1}H NMR (500 MHz, $CDCl_3$) of compound **3s**

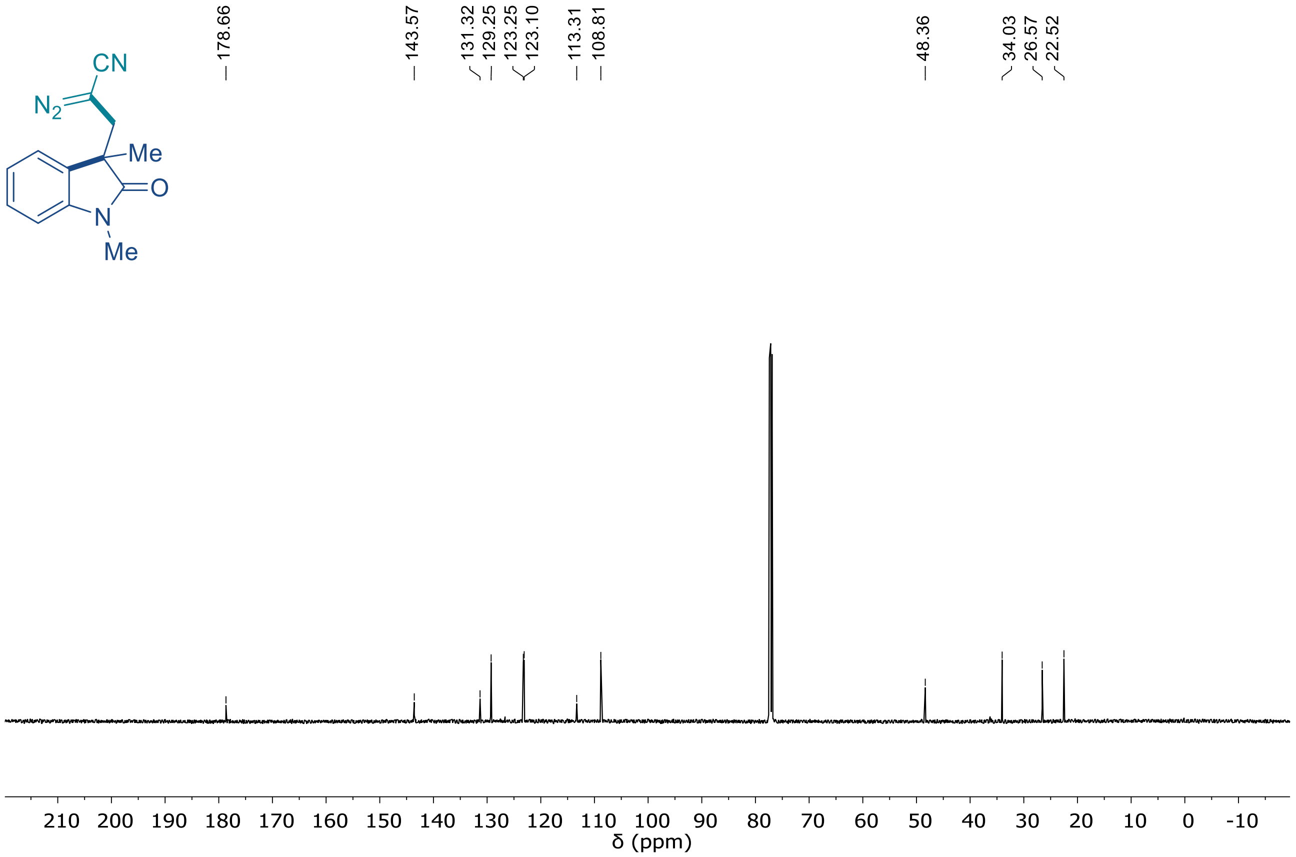

^{13}C NMR (126 MHz, $CDCl_3$) of compound **3s**

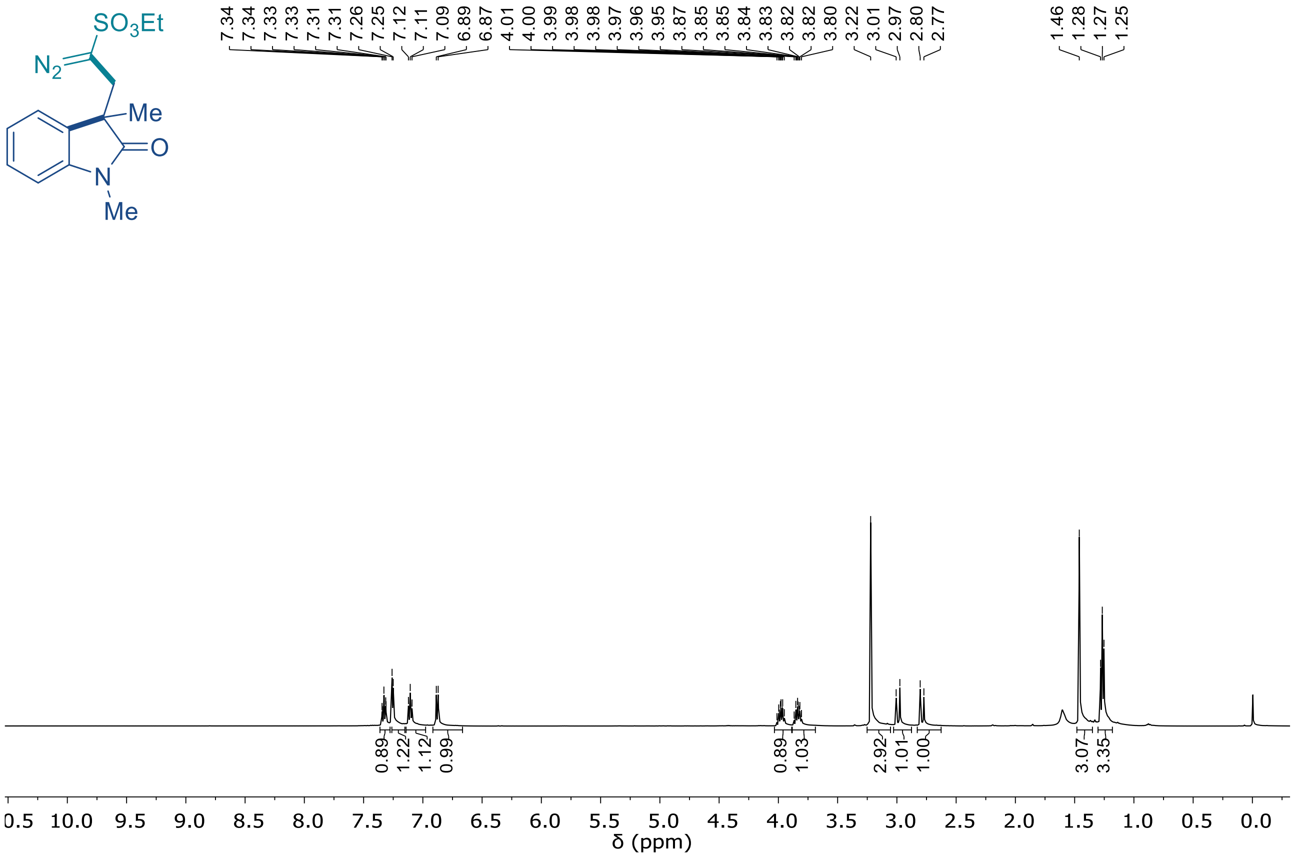

^{1}H NMR (500 MHz, $CDCl_3$) of compound **3t**

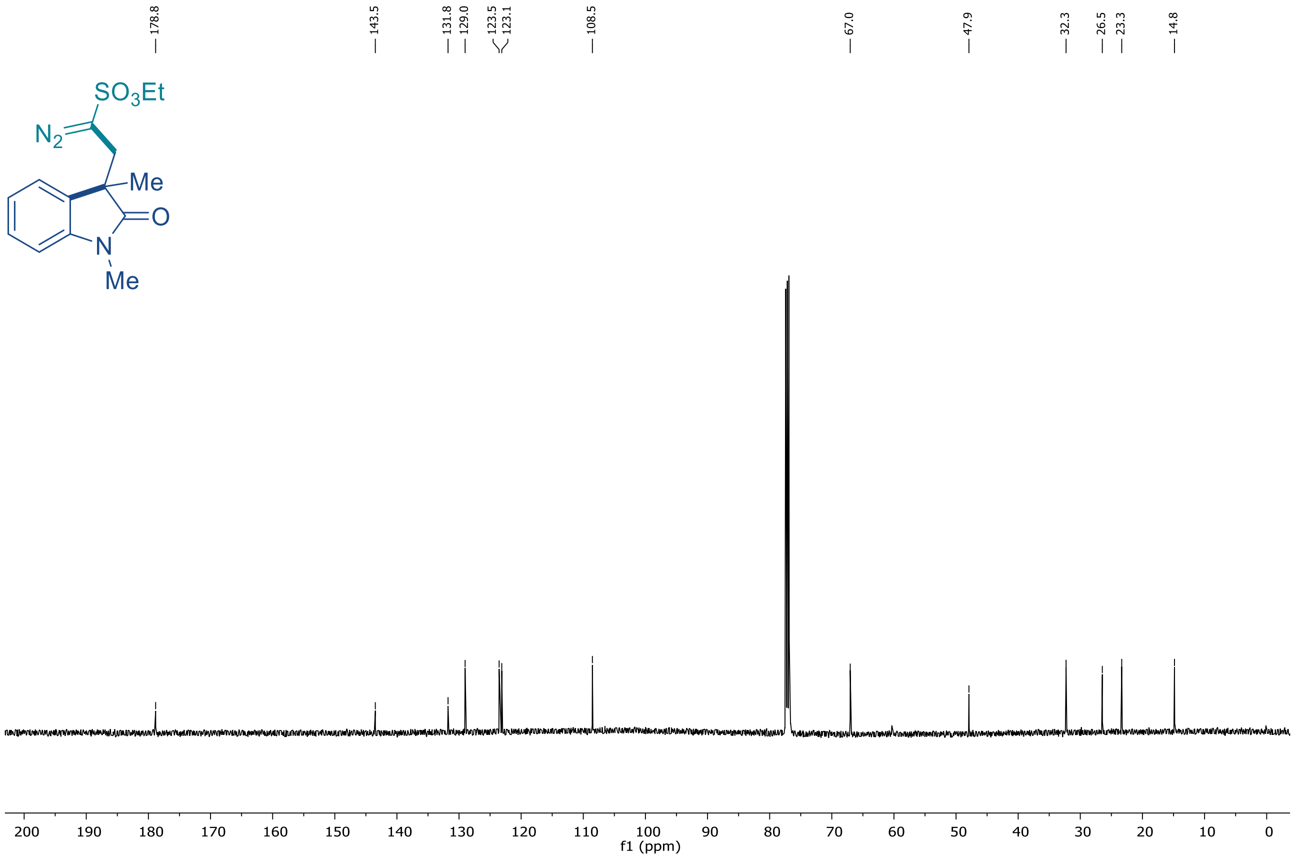

^{13}C NMR (126 MHz, $CDCl_3$) of compound **3t**

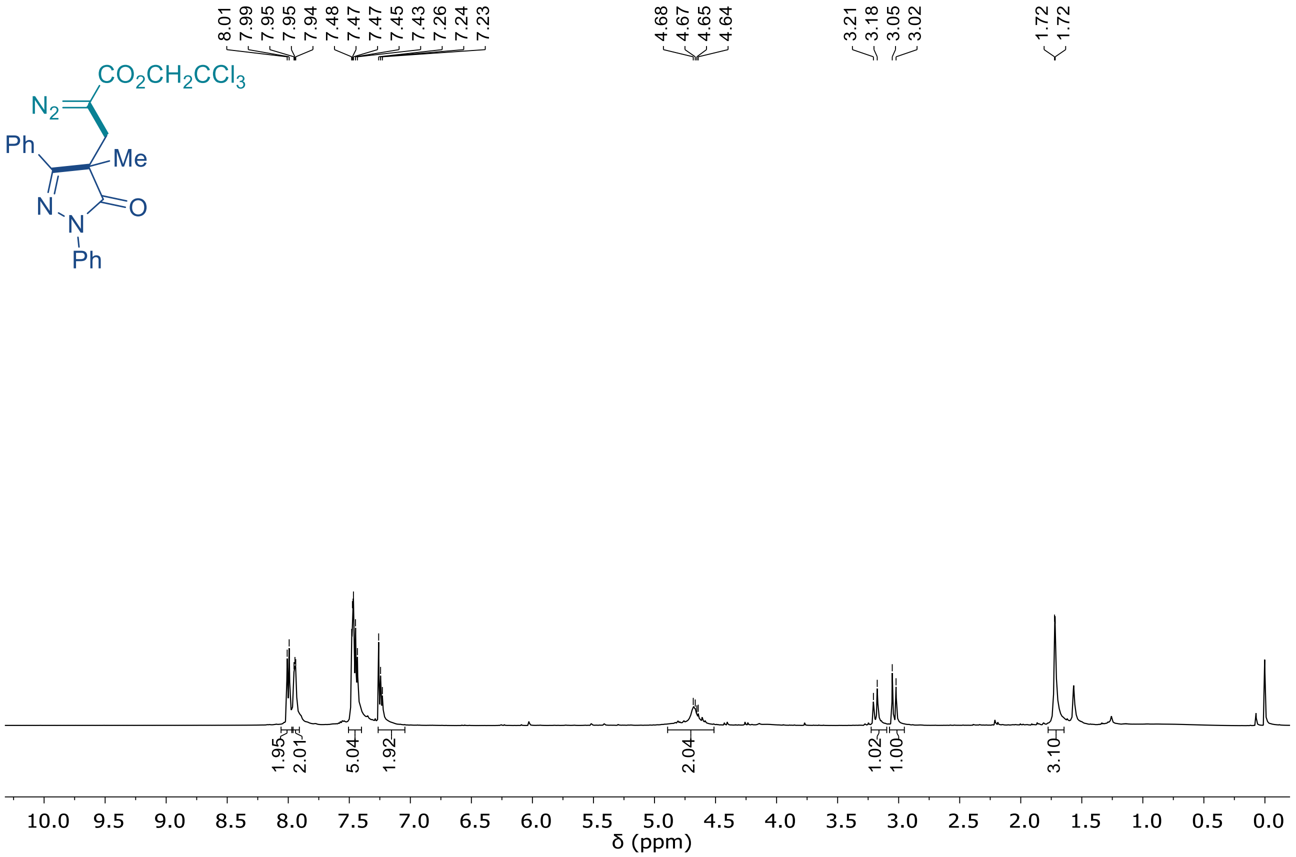

^{1}H NMR (500 MHz, $CDCl_3$) of compound **3u**

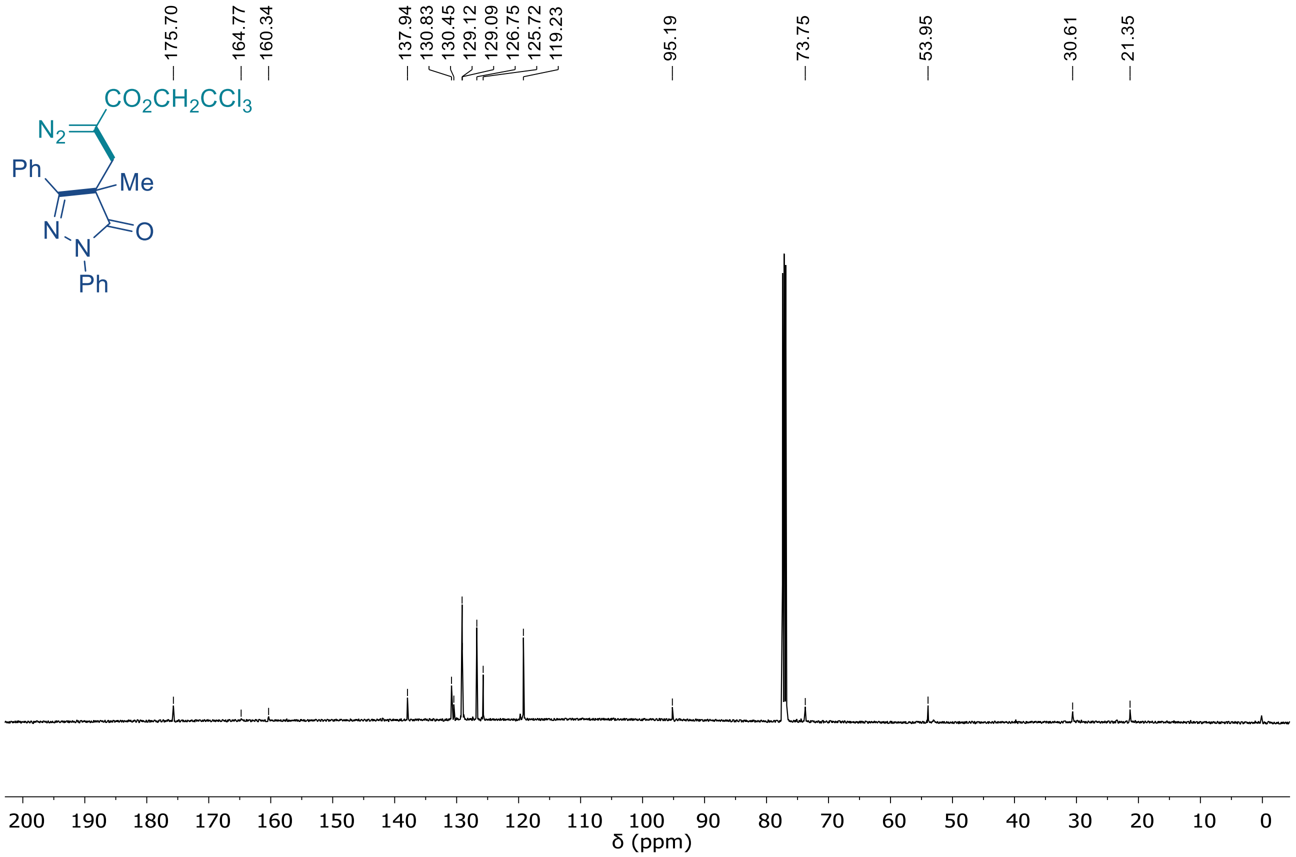

^{13}C NMR (126 MHz, $CDCl_3$) of compound **3u**

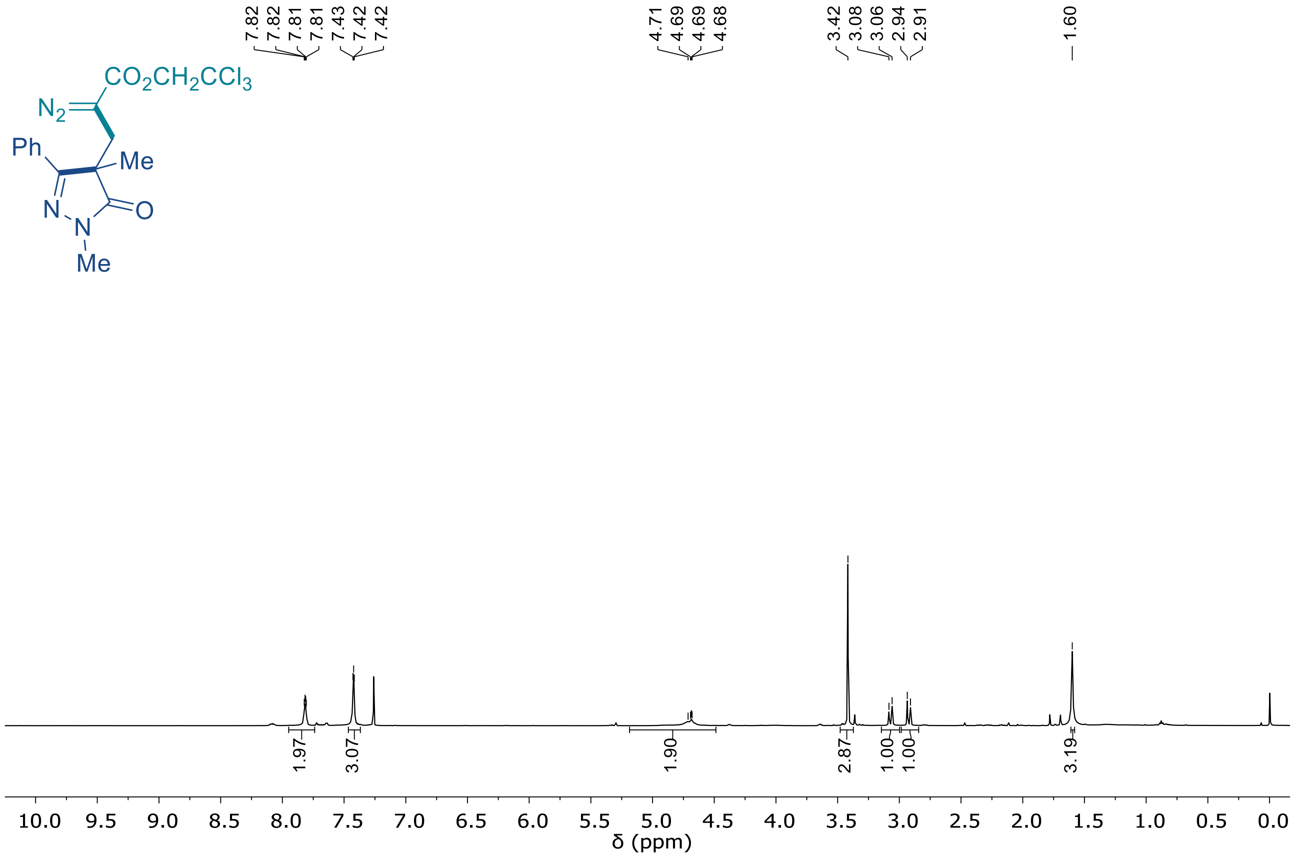

^{1}H NMR (600 MHz, $CDCl_3$) of compound **3v**

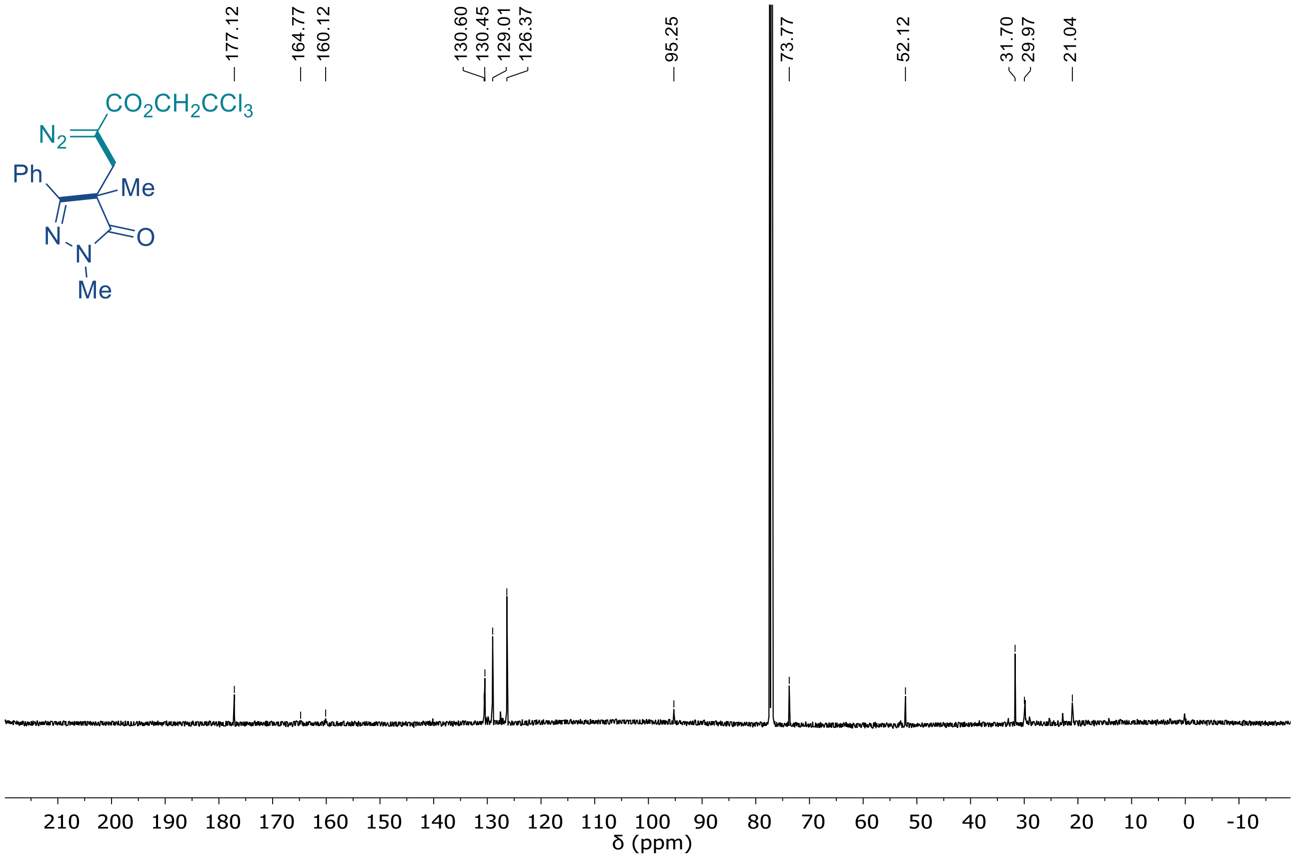

^{13}C NMR (126 MHz, $CDCl_3$) of compound **3v**

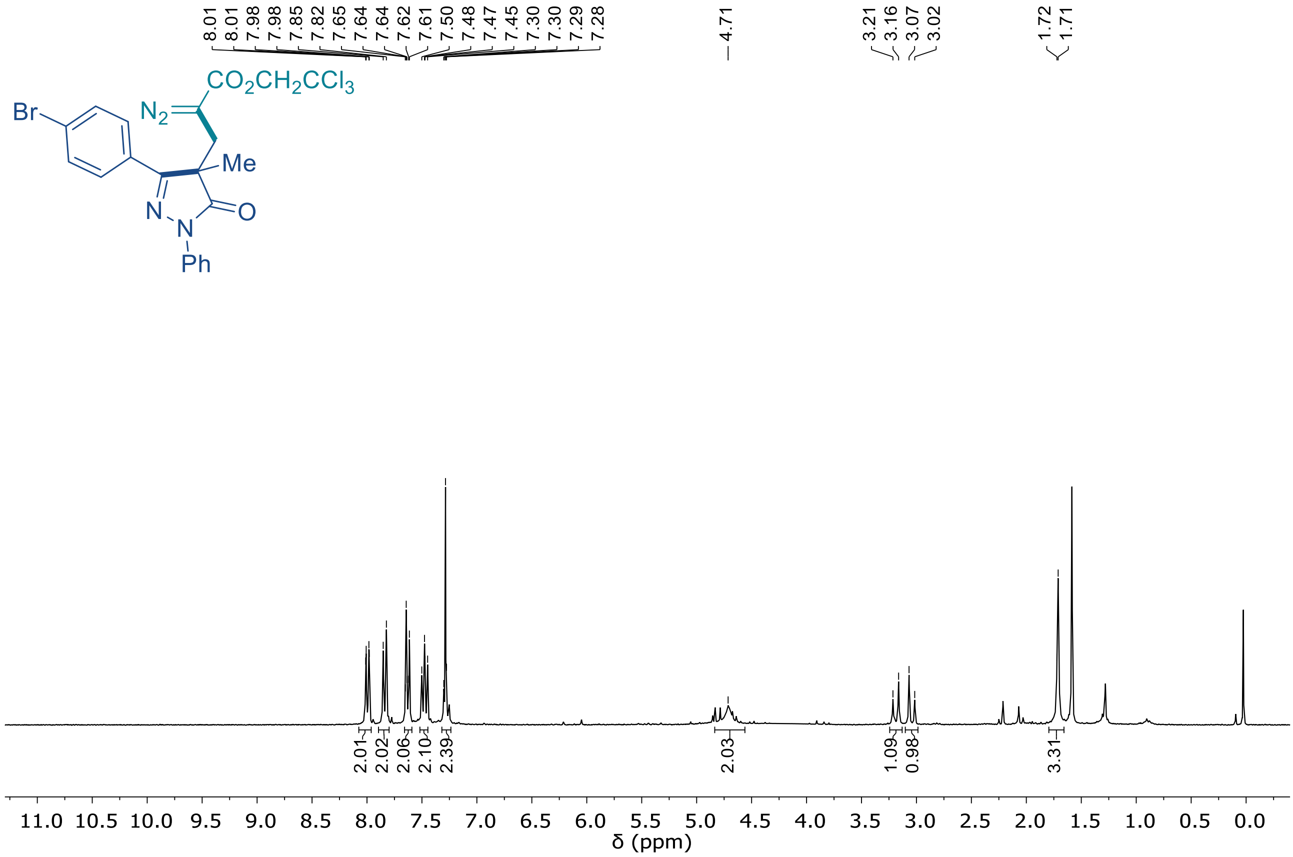

^{1}H NMR (300 MHz, $CDCl_3$) of compound **3w**

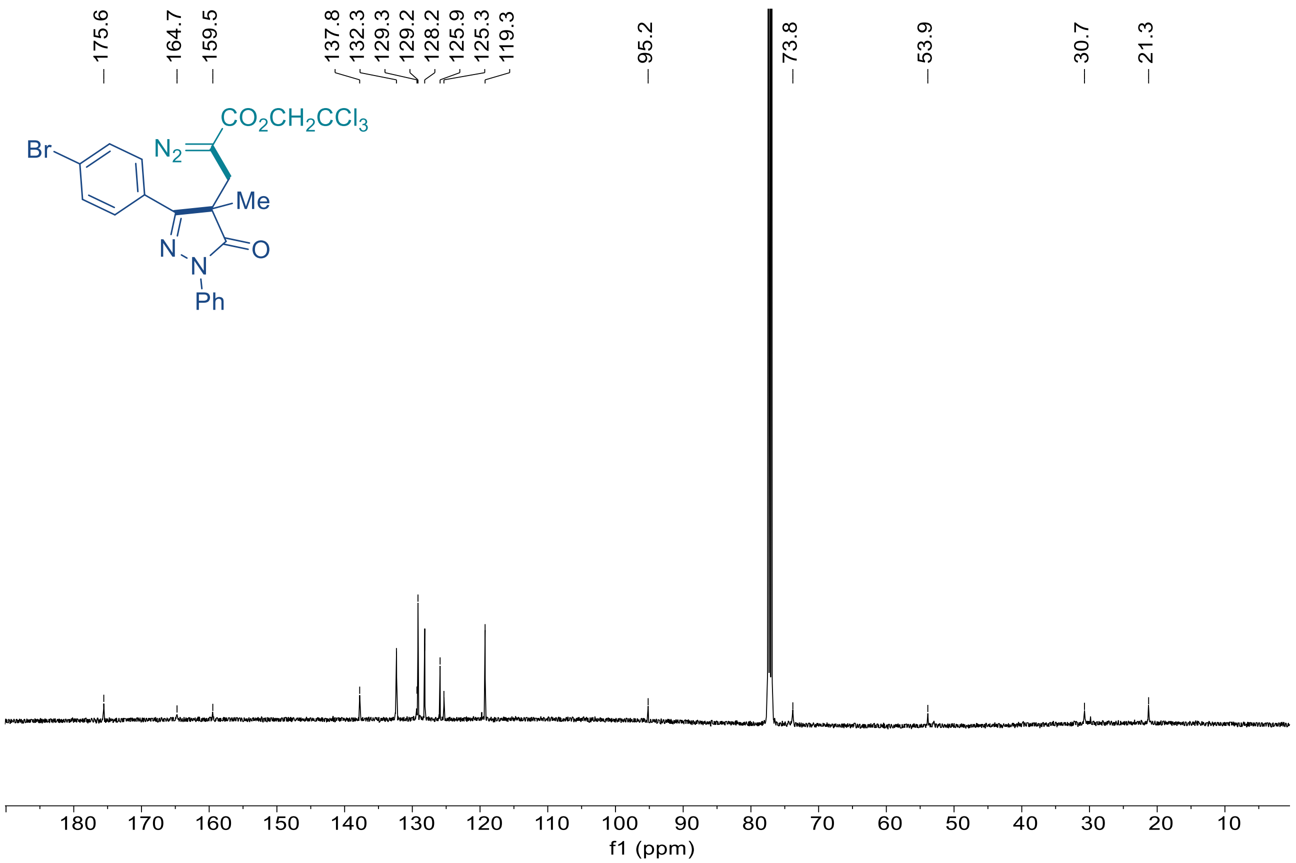

^{13}C NMR (126 MHz, $CDCl_3$) of compound **3w**

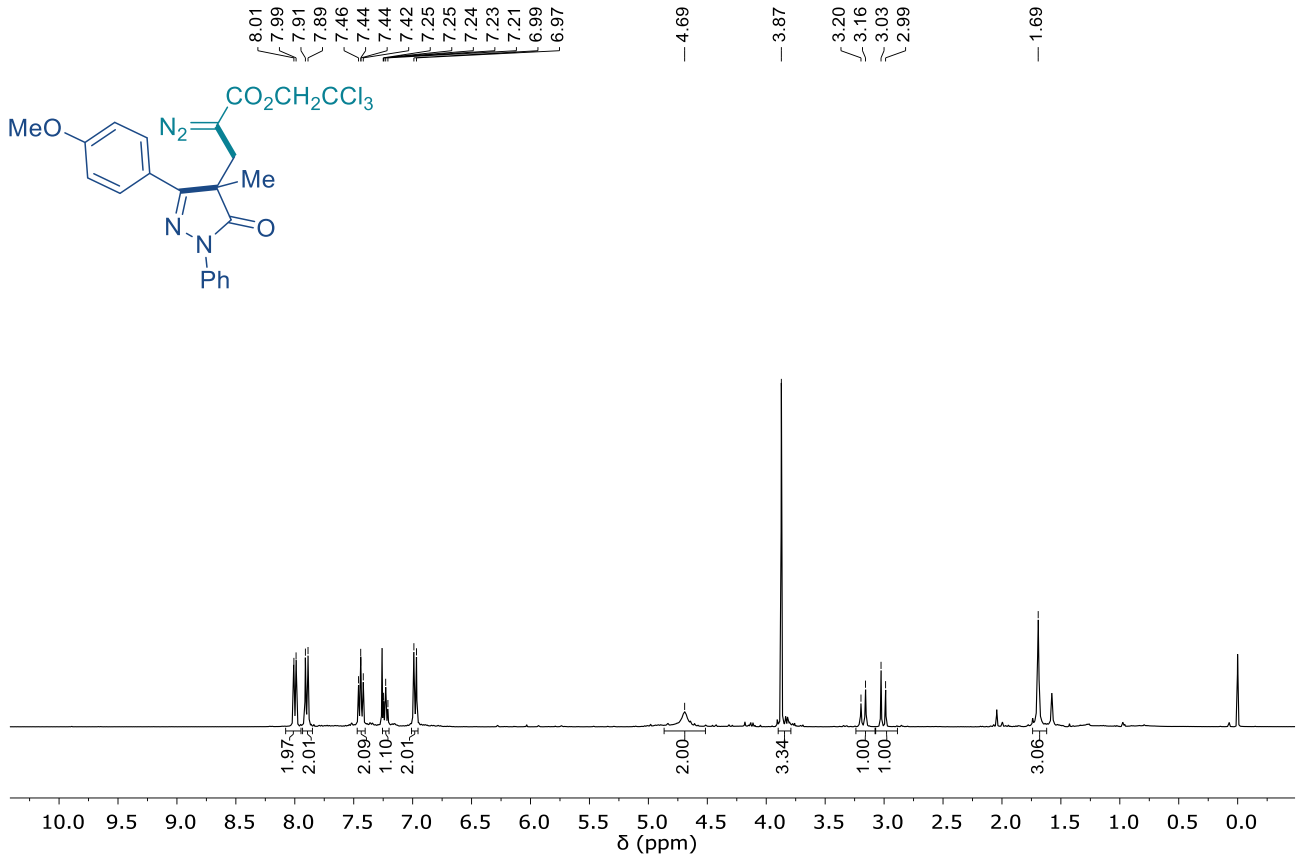

^{1}H NMR (400 MHz, $CDCl_3$) of compound **3x**

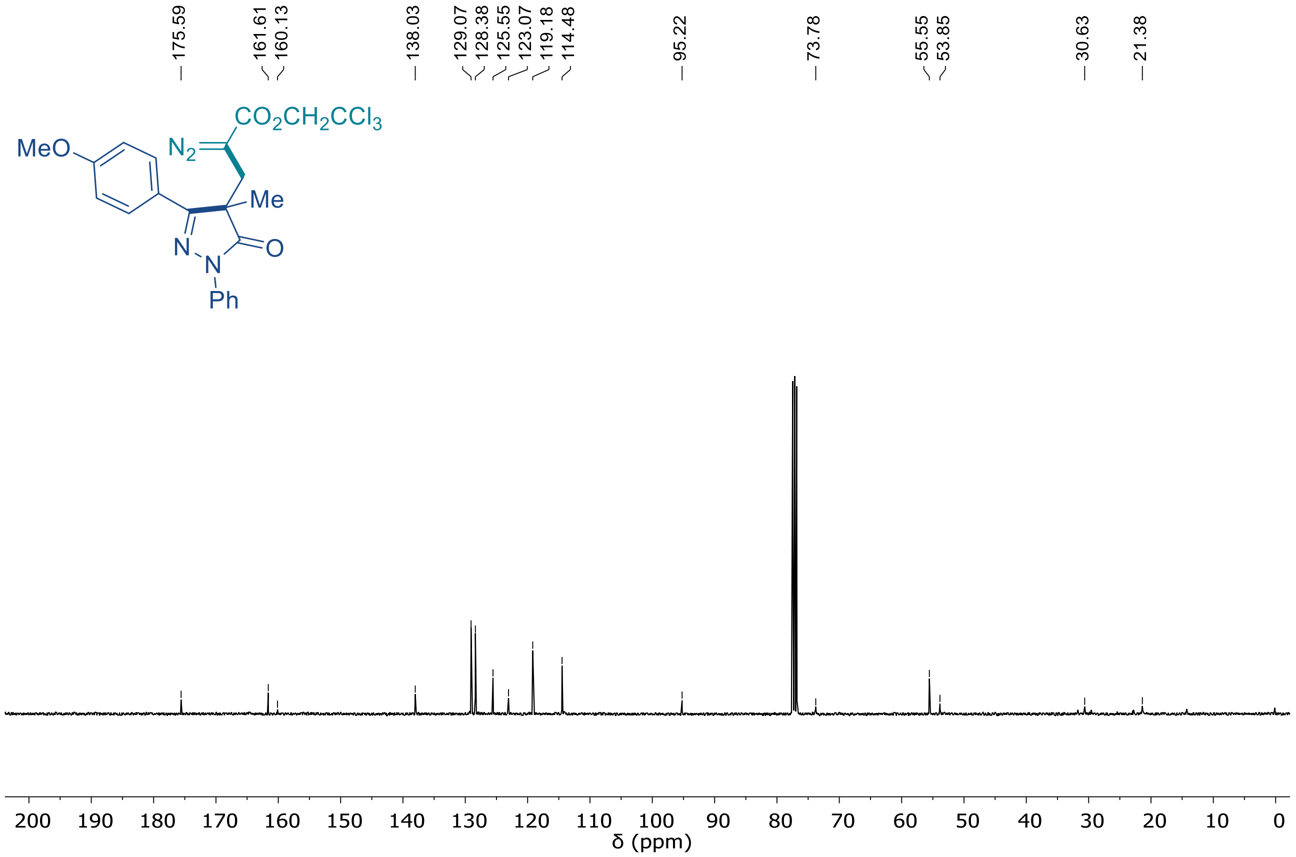

^{13}C NMR (126 MHz, $CDCl_3$) of compound **3x**

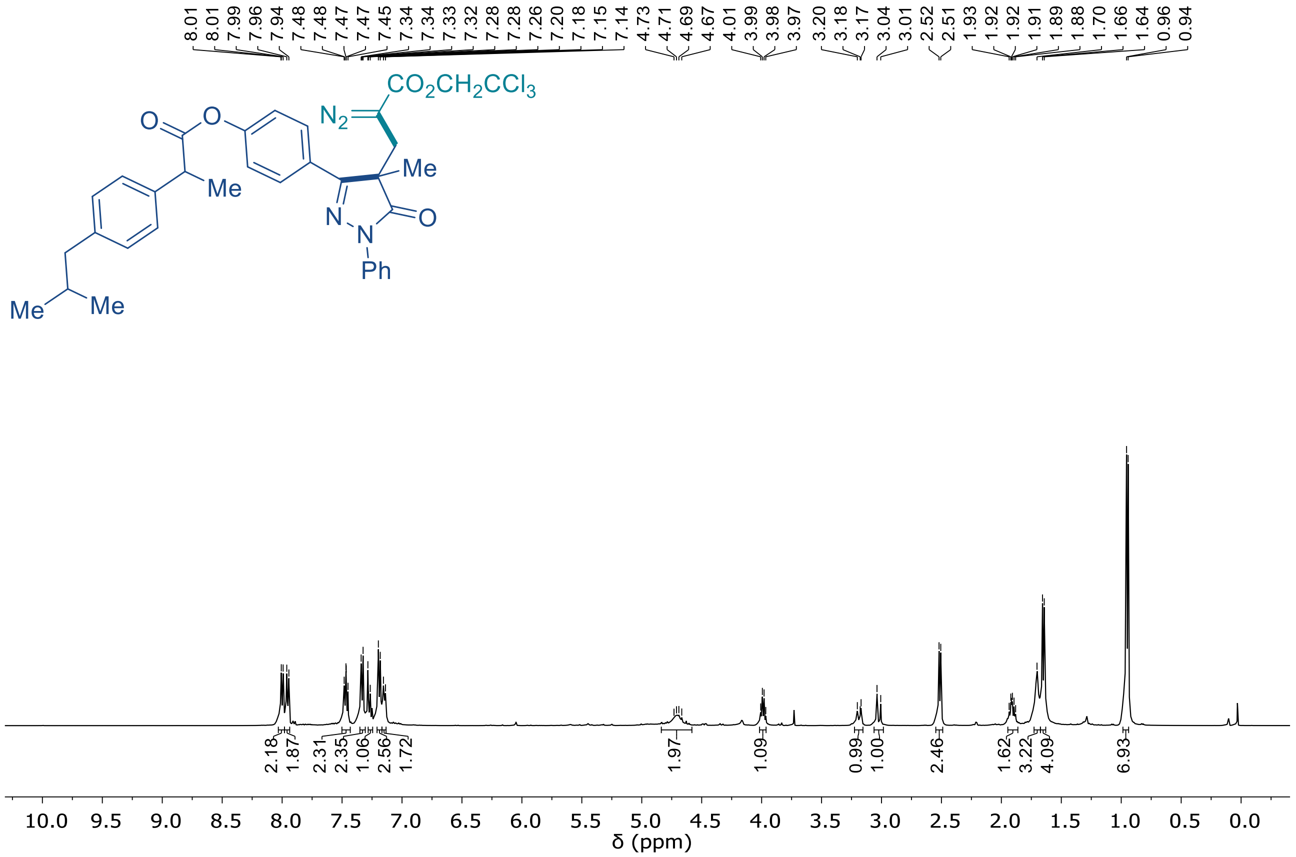

^{1}H NMR (500 MHz, $CDCl_3$) of compound **3y**

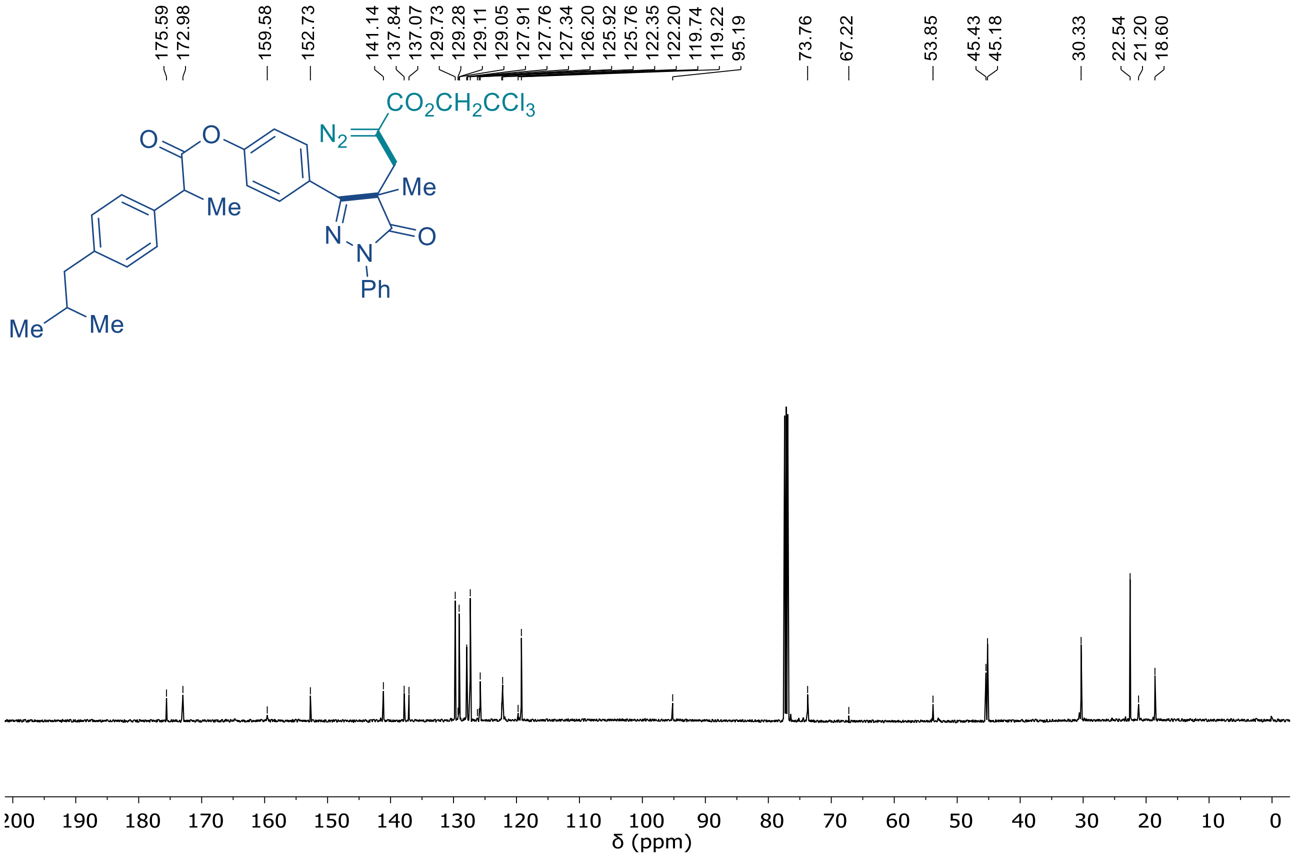

^{13}C NMR (126 MHz, $CDCl_3$) of compound **3y**

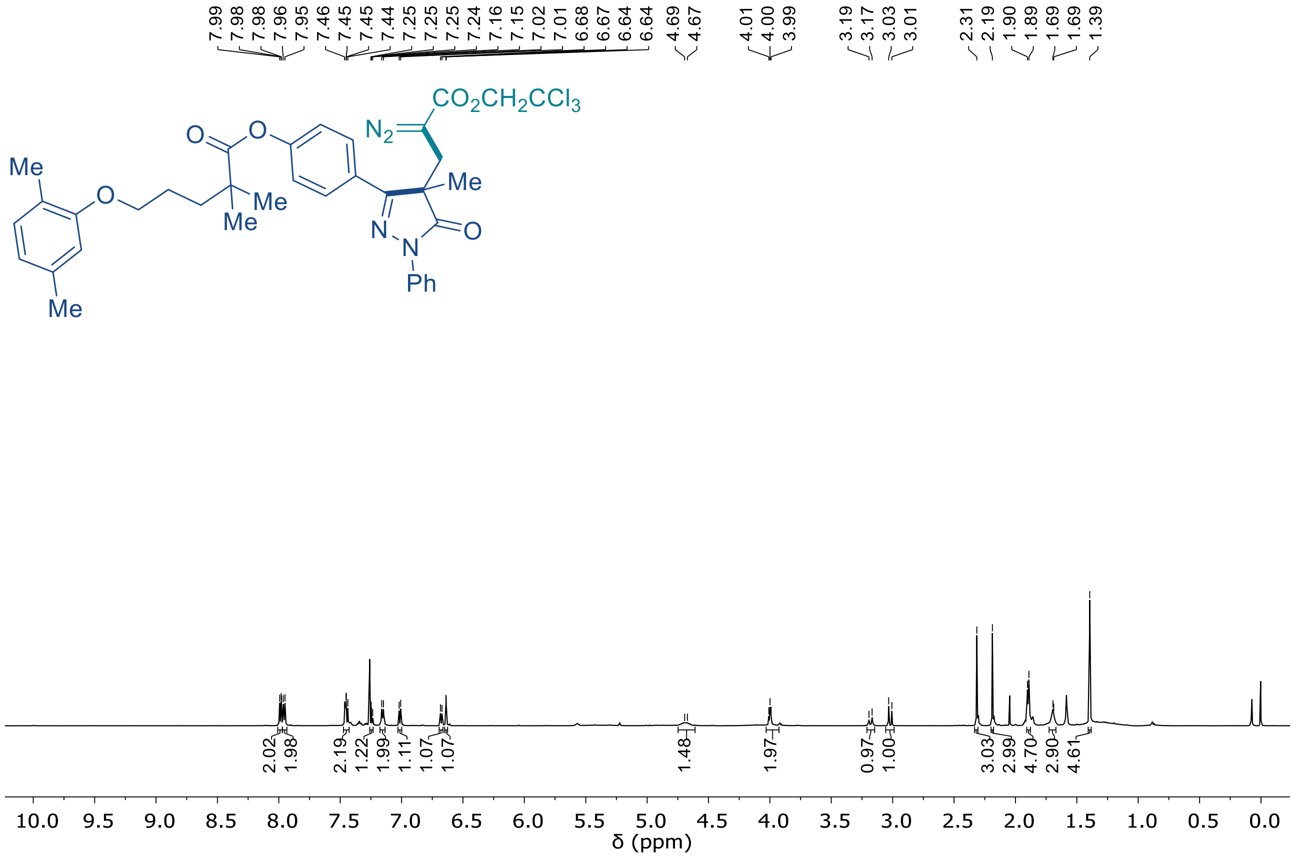

^{1}H NMR (500 MHz, $CDCl_3$) of compound **3z**

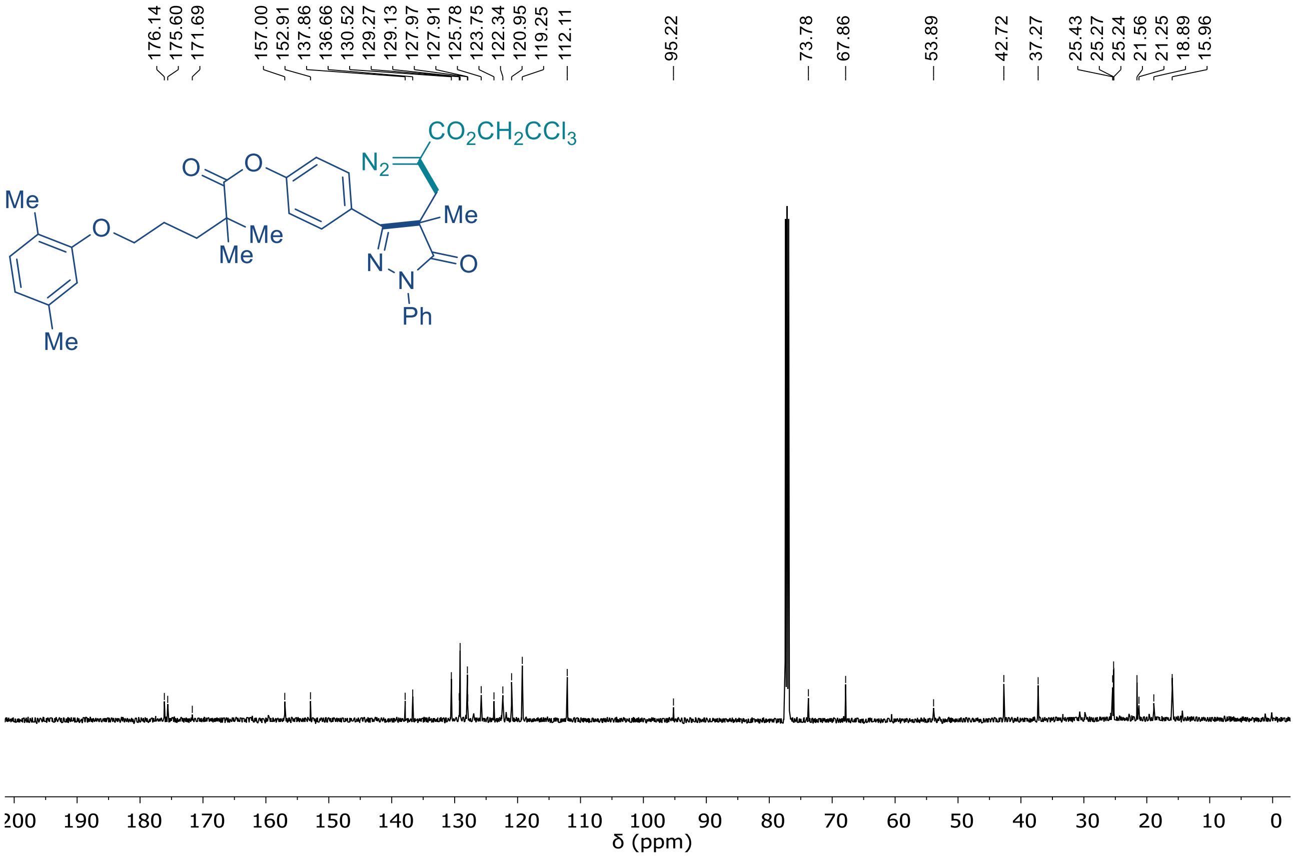

^{13}C NMR (126 MHz, CDCl$_3$) of compound **3z**

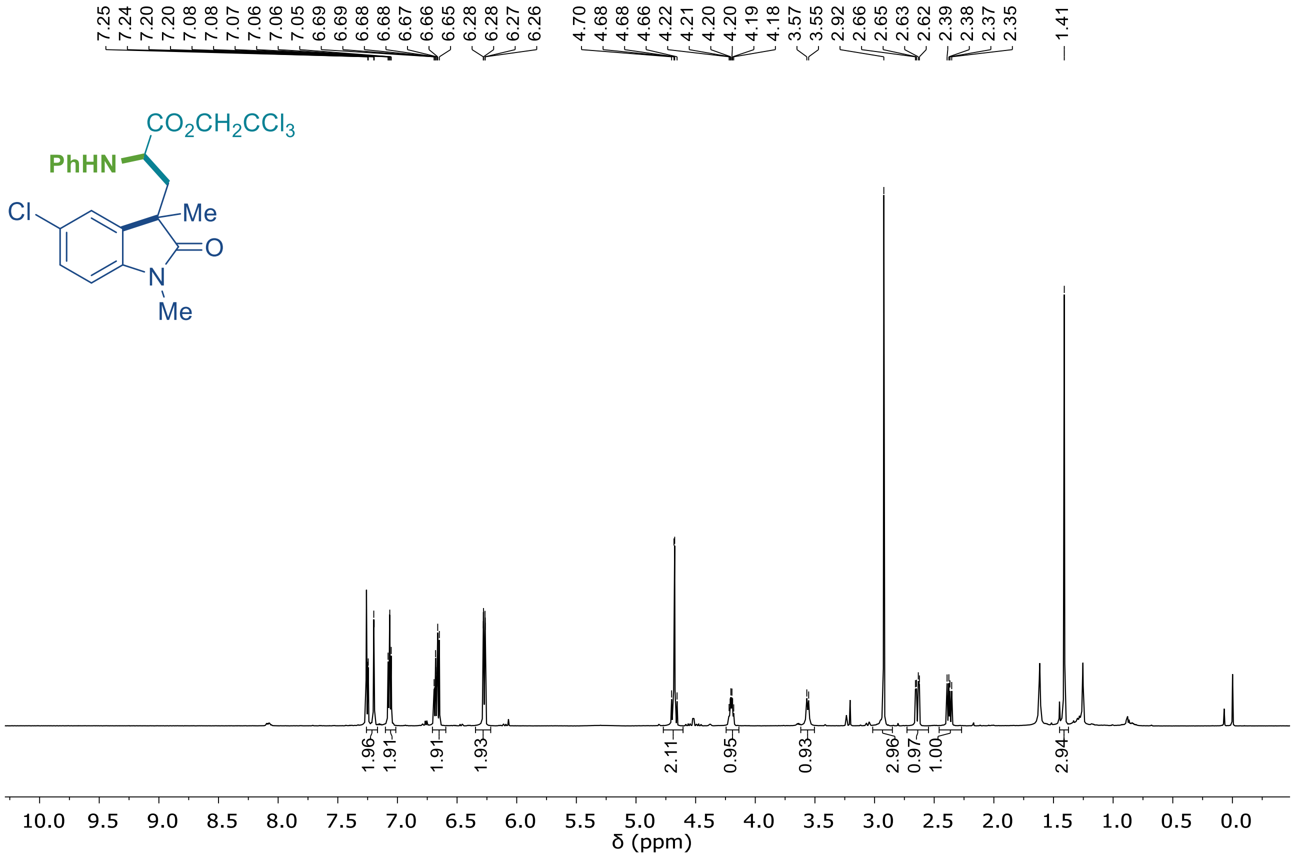

^{1}H NMR (600 MHz, CDCl$_3$) of compound **4**

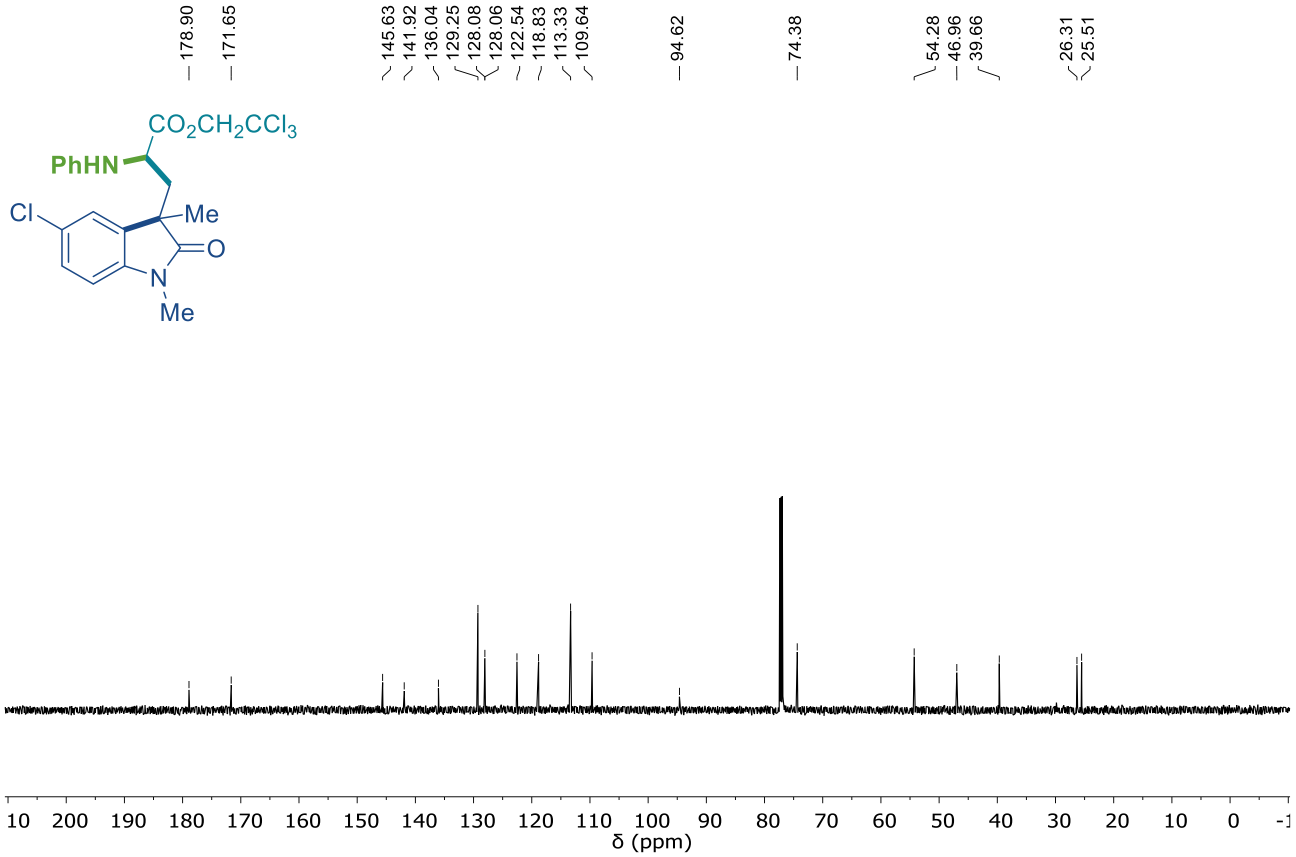

^{13}C NMR (151 MHz, $CDCl_3$) of compound **4**

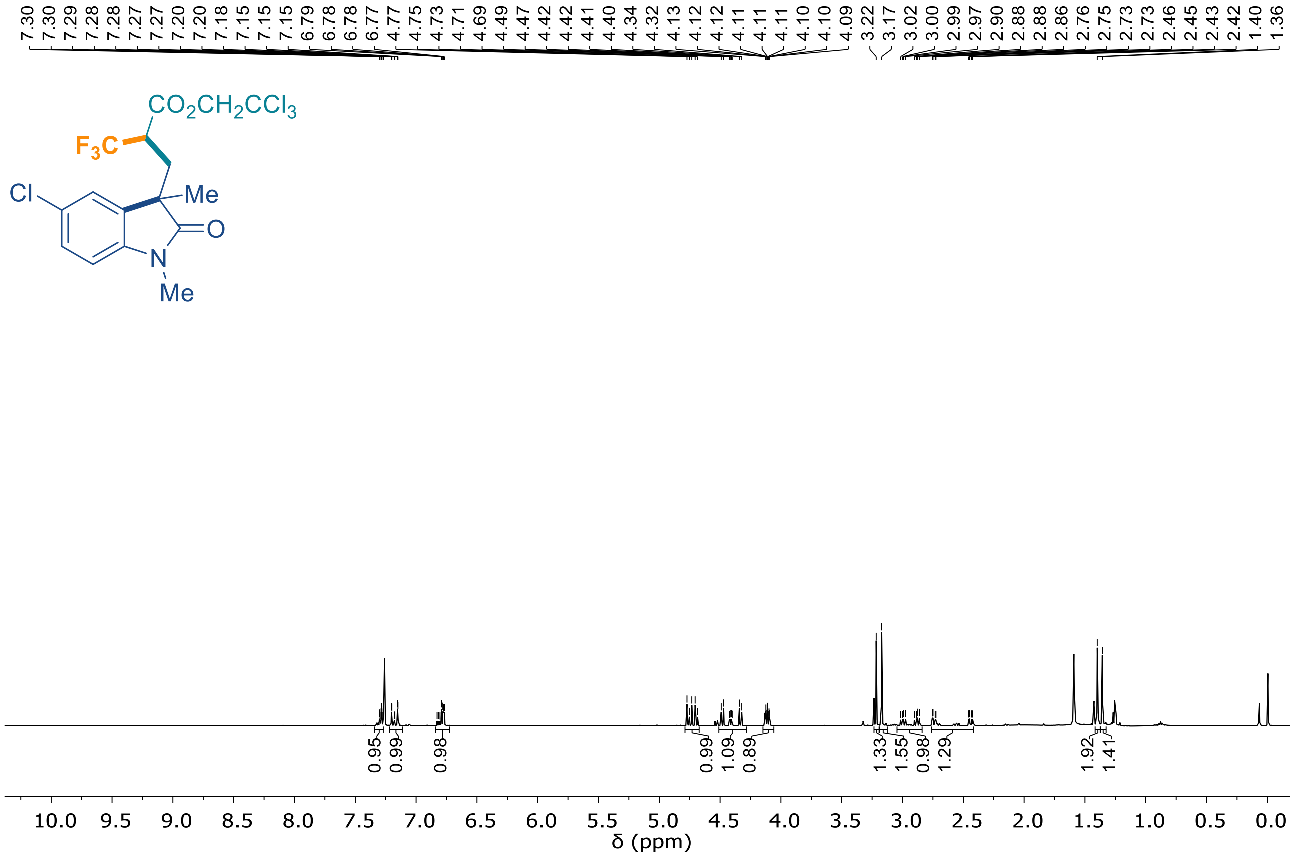

^{1}H NMR (600 MHz, $CDCl_3$) of compound **5**

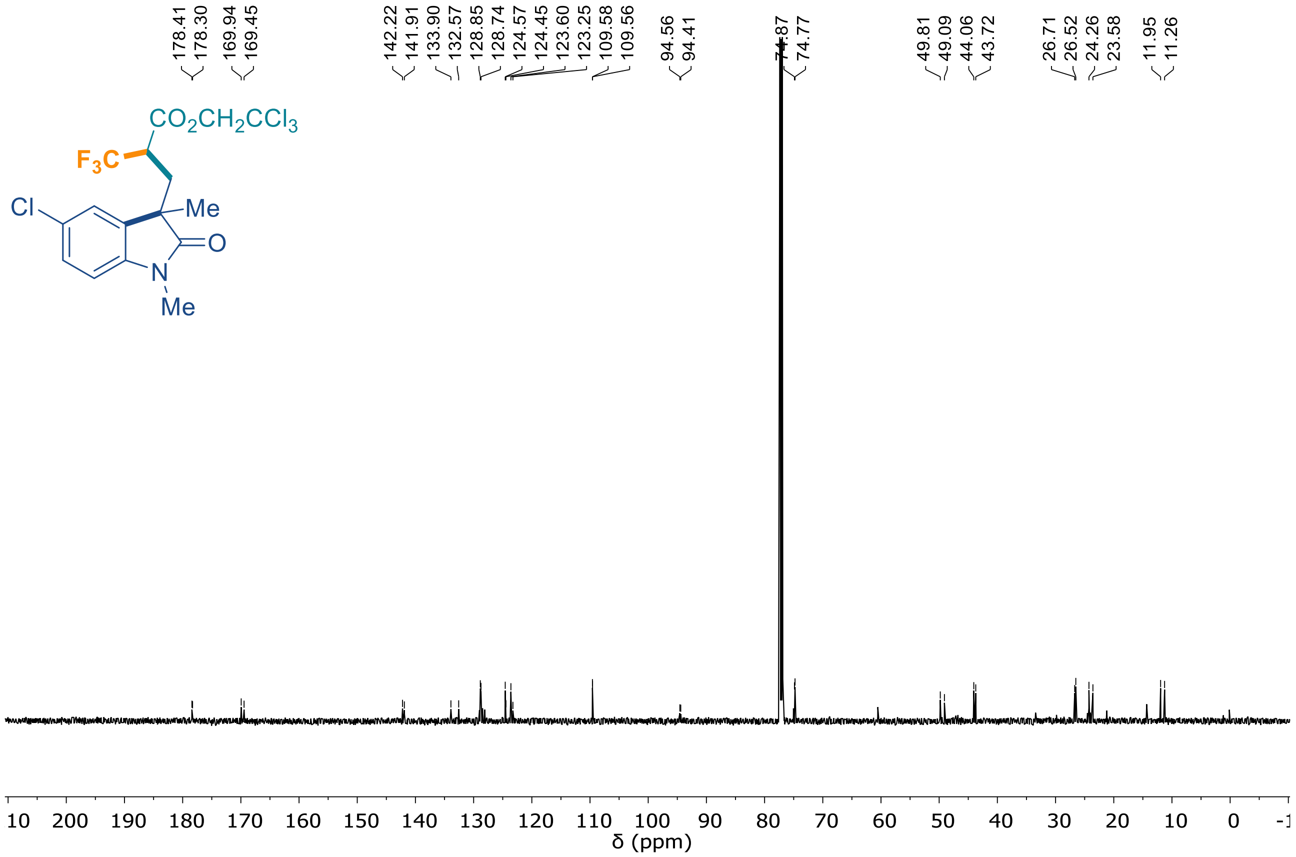

^{13}C NMR (151 MHz, $CDCl_3$) of compound **5**

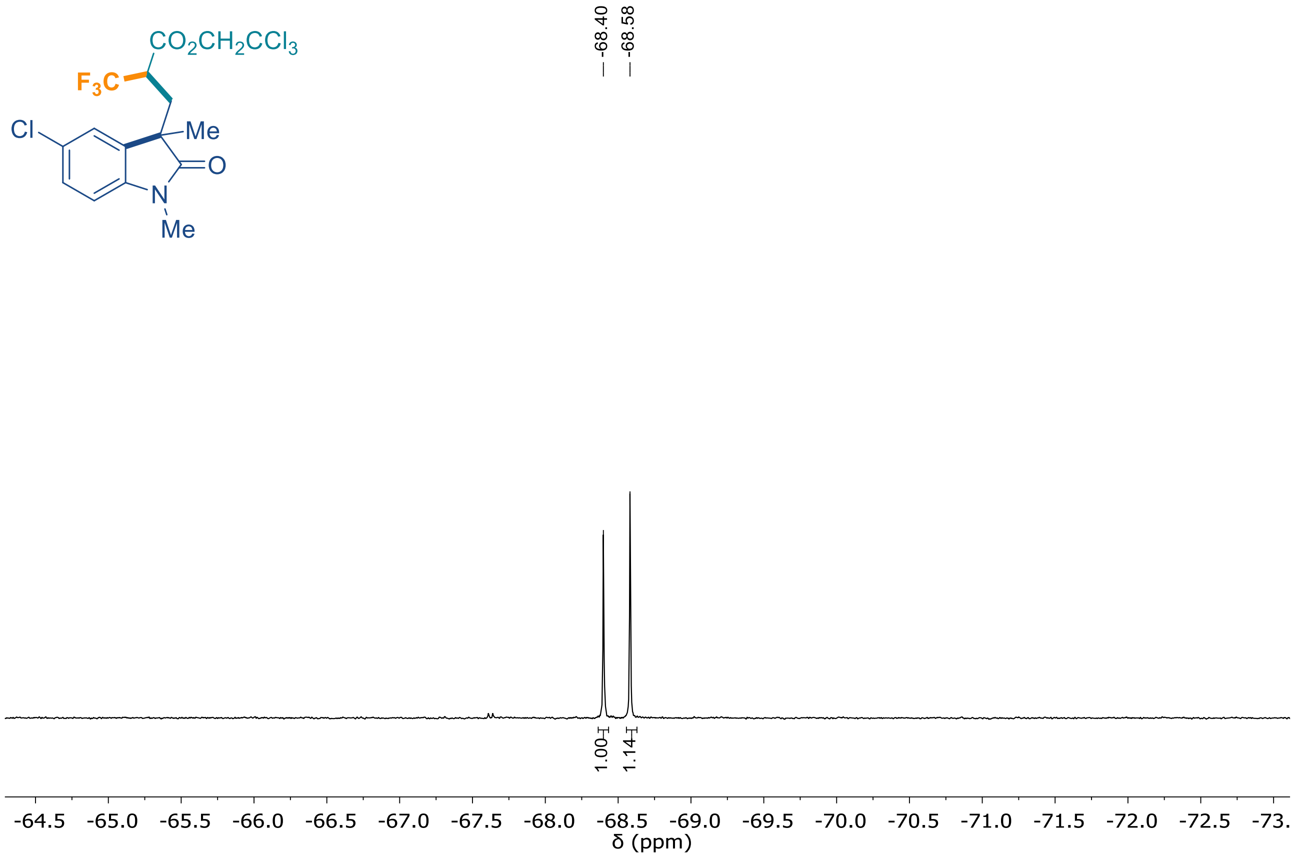

^{19}F NMR (282 MHz, $CDCl_3$) of compound **5**

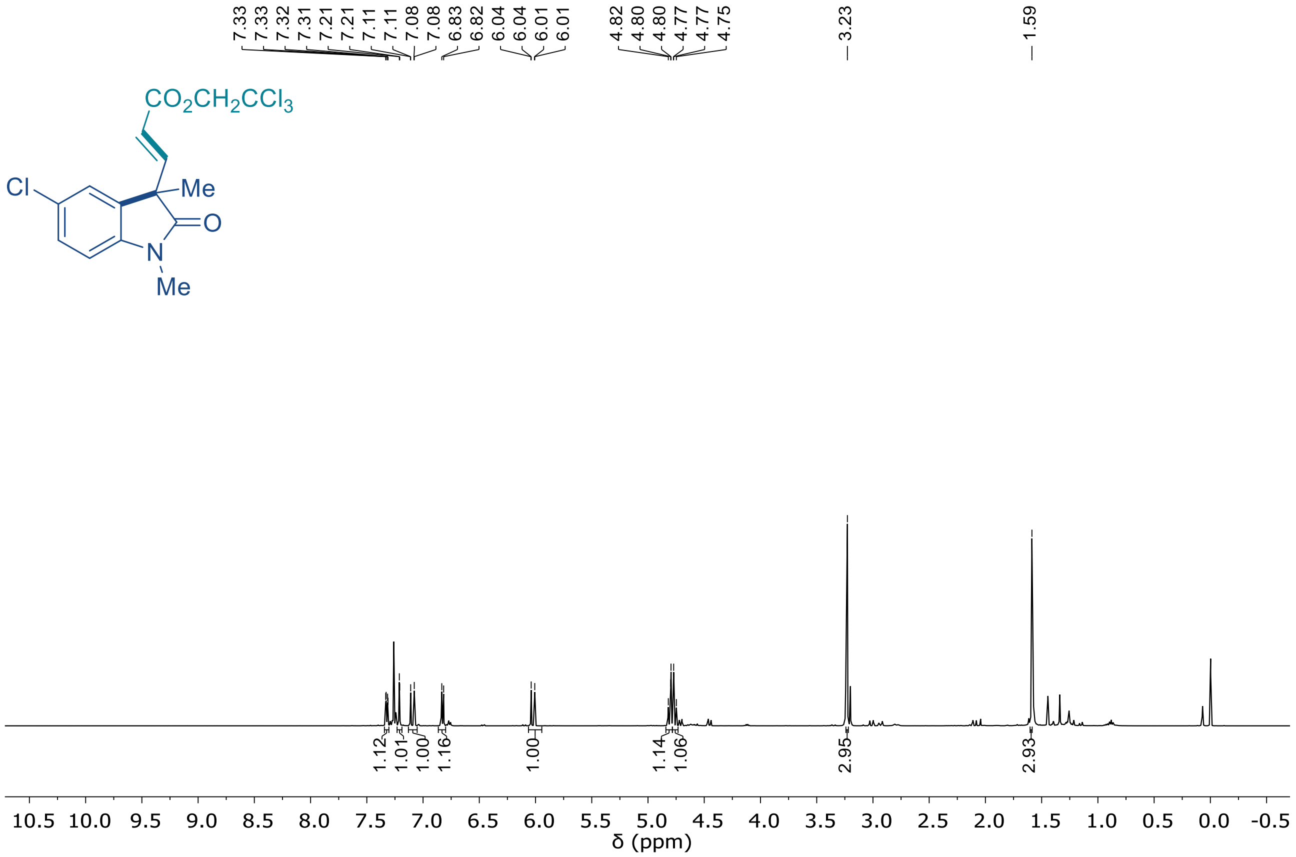

^{1}H NMR (600 MHz, $CDCl_3$) of compound **6-(*E*)**

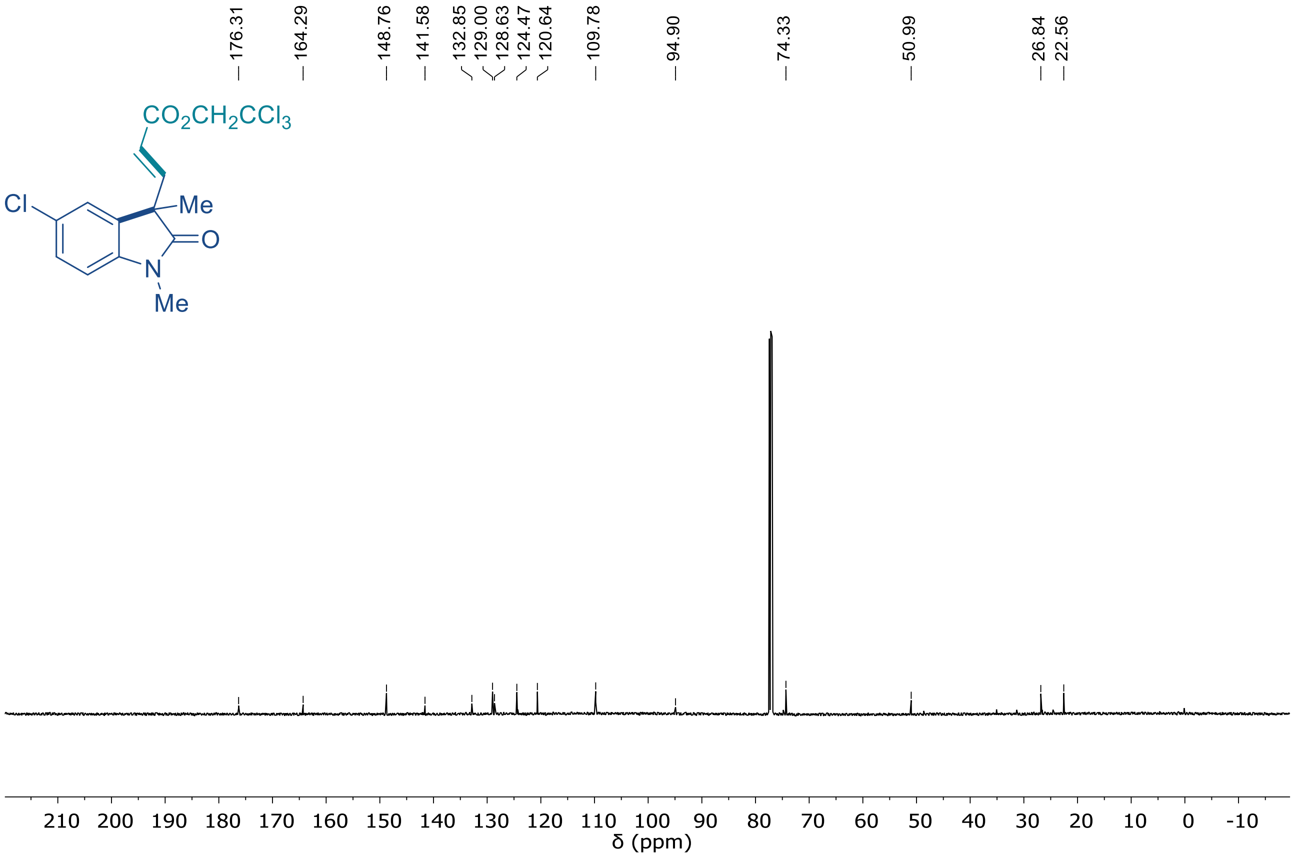

^{13}C NMR (126 MHz, $CDCl_3$) of compound **6-(*E*)**

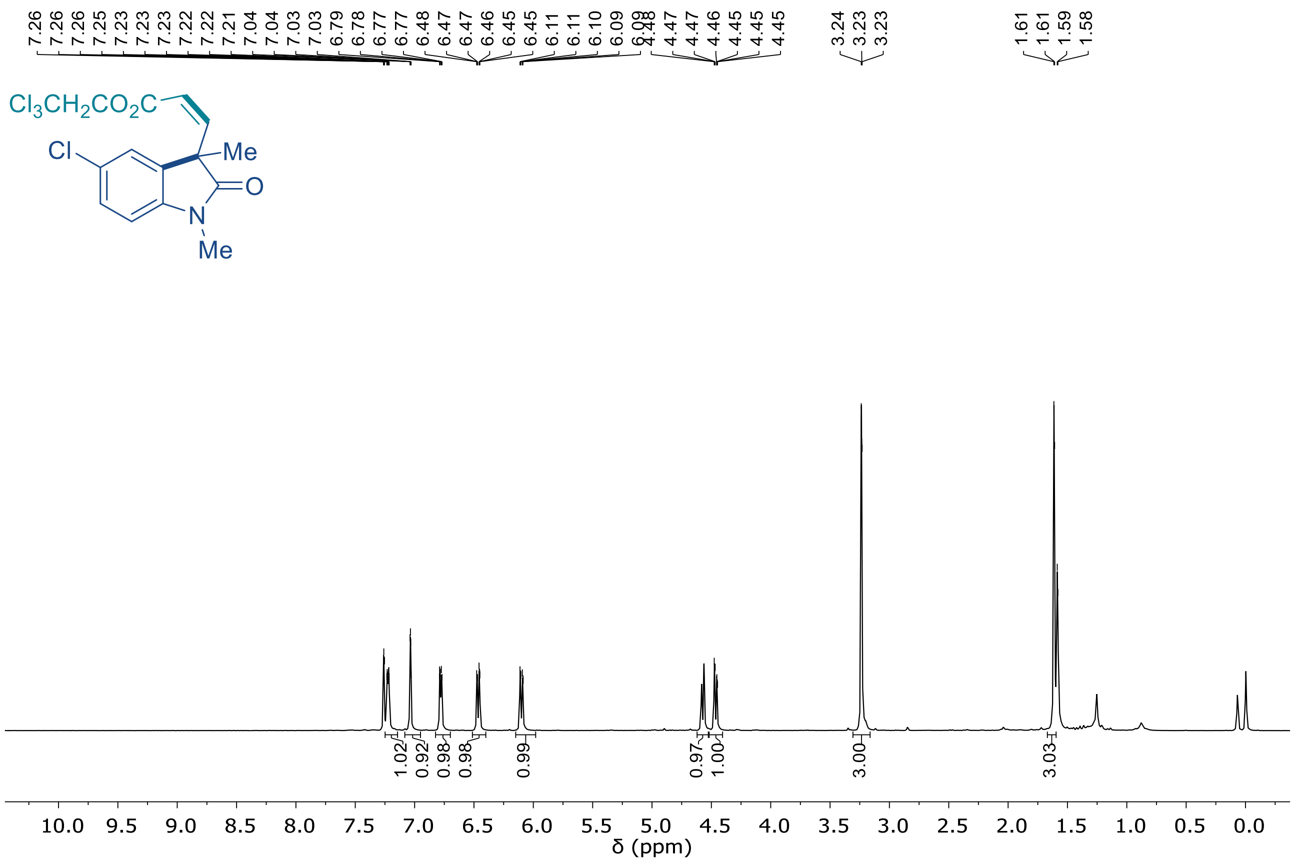

^{1}H NMR (600 MHz, $CDCl_3$) of compound **6-(*Z*)**

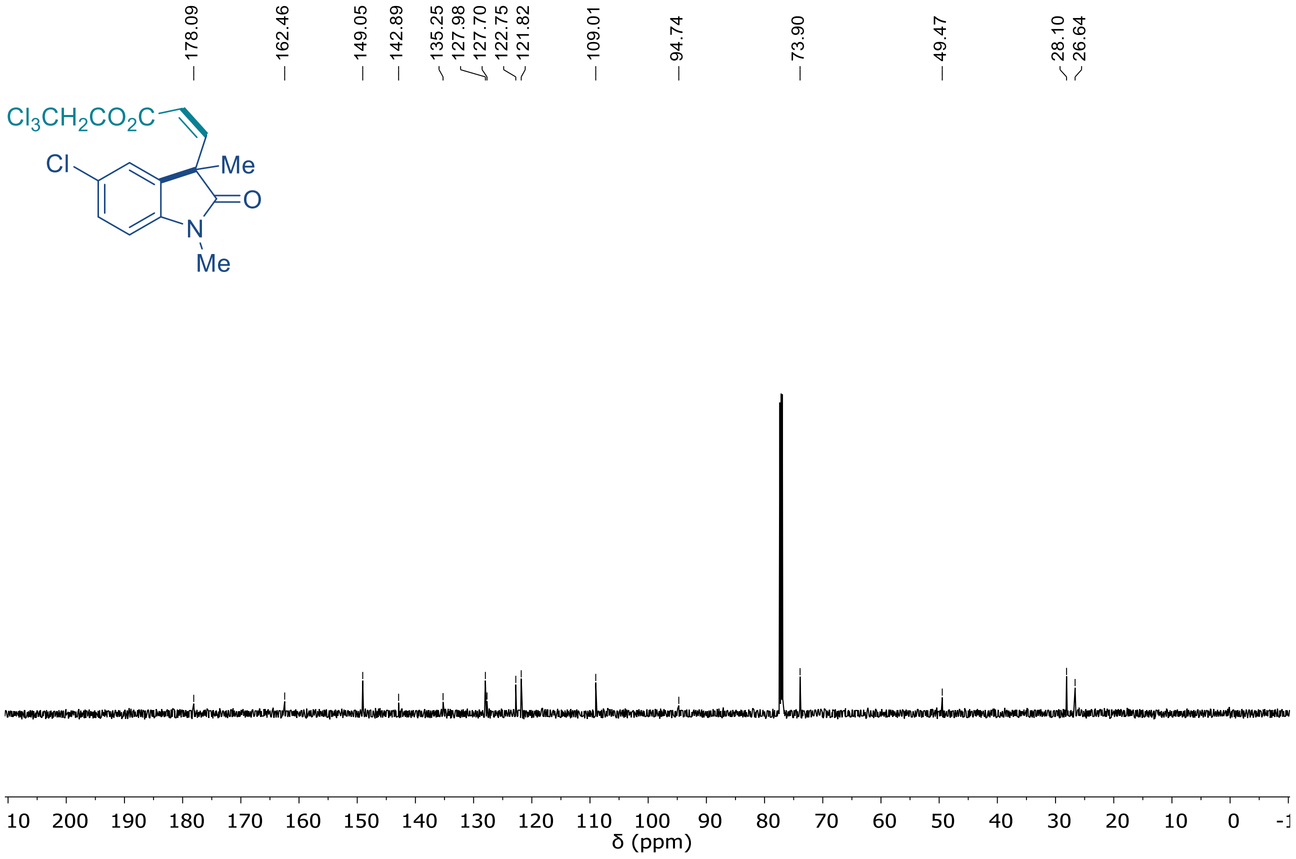

^{13}C NMR (151 MHz, $CDCl_3$) of compound **6-(*Z*)**

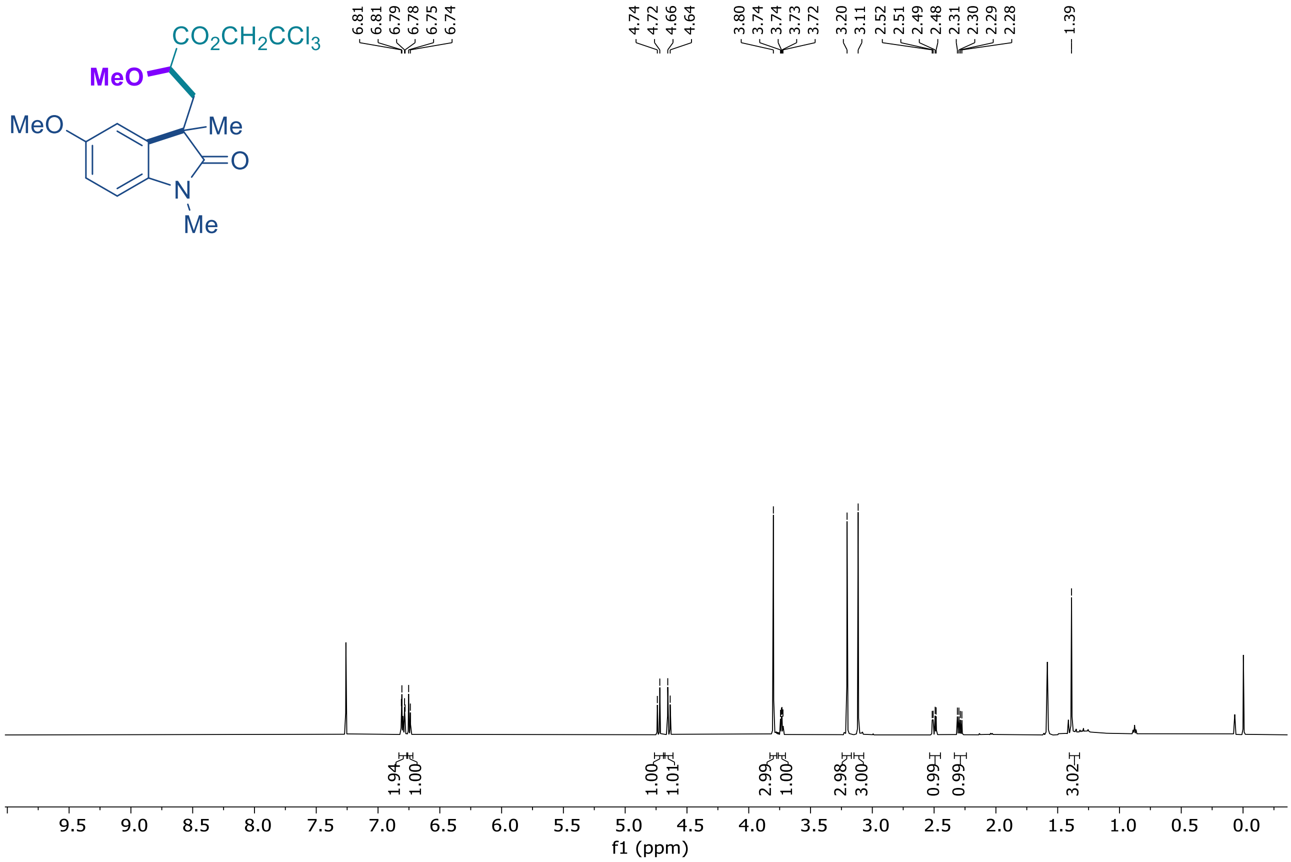

^{1}H NMR (600 MHz, $CDCl_3$) of compound **7**

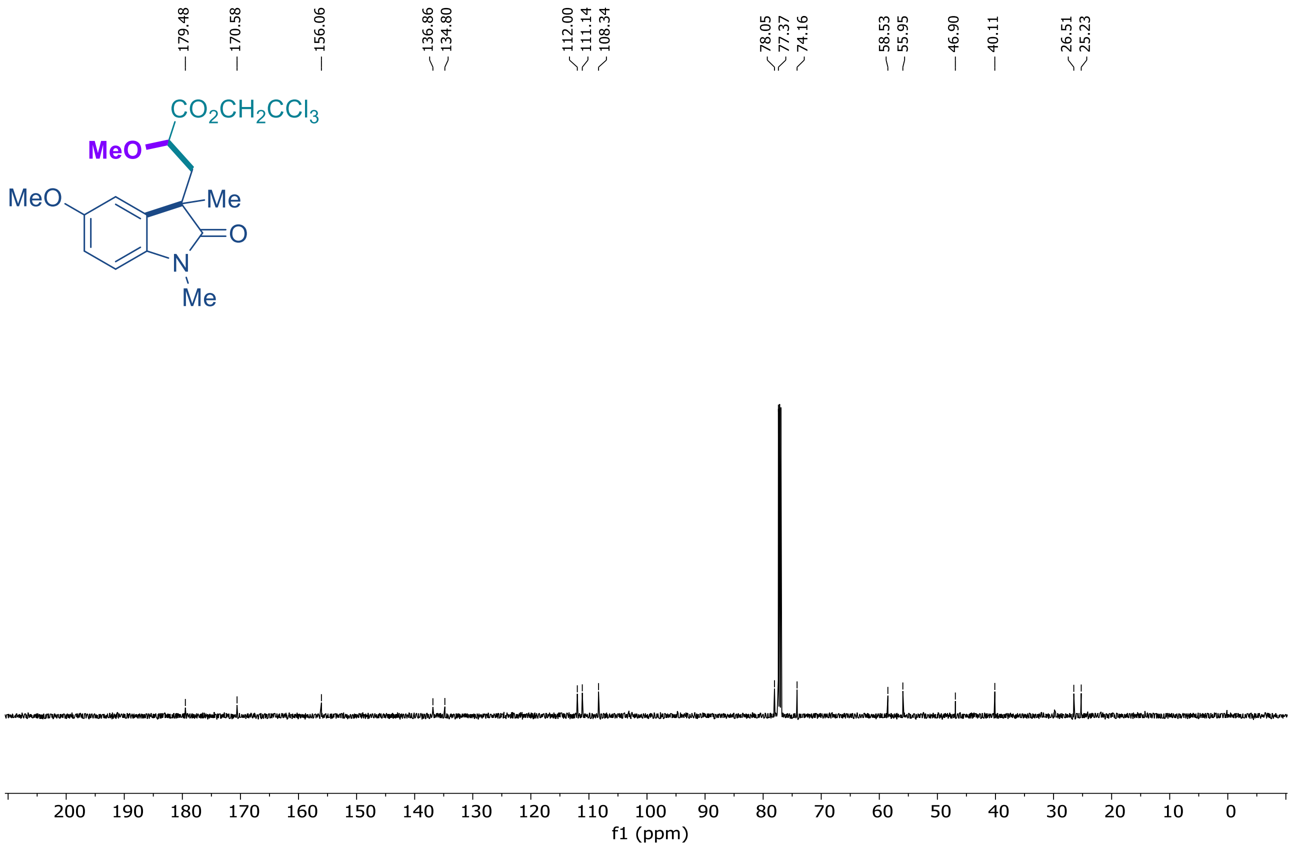

^{13}C NMR (151 MHz, $CDCl_3$) of compound **7**
